# Gamma oscillations provide a stable geometric scaffold for color representation in primate inferior temporal cortex

**DOI:** 10.1038/s42003-026-10652-8

**Published:** 2026-07-11

**Authors:** Chengpeng Li, Isao Hasegawa, Hisashi Tanigawa

**Affiliations:** 1 School of Brain Science and Brain Medicine, Zhejiang University, Hangzhou, China; 2 College of Biomedical Engineering and Instrument Science, Zhejiang University, Hangzhou, China; 3 MOE Frontier Science Center for Brain Science and Brain-Machine Integration, School of Brain Science and Brain Medicine, Zhejiang University, Hangzhou, China; 4 National Key Laboratory of Brain and Computer Intelligence, Zhejiang University, Hangzhou, China; 5 NHC and CAMS Key Laboratory of Medical Neurobiology, Zhejiang University, Hangzhou, China; 6 Department of Physiology, Niigata University School of Medicine, Niigata, Japan

**Keywords:** Extrastriate cortex, Colour vision

## Abstract

To bridge the gap between neural geometry and oscillatory dynamics, we analyzed gamma-band oscillations in macaque inferior temporal cortex using large-scale electrocorticography. We identified an “oscillatory manifold”—a stable, low-dimensional geometric structure embedded within oscillatory waveforms—that encodes color information. Within this manifold, trajectories for different colors were segregated via color-specific amplitude modulations of a shared oscillatory carrier. While the absolute spatial separation of these trajectories rapidly decayed following an initial stimulus-locked transient, their scale-invariant topological shape was conserved. Although stimulus shape was decodable from gamma-band power, it lacked the stable geometric structure observed for color. This contrast confirms that the oscillatory manifold is a specific representational format rather than a generic byproduct of neural activation. Consequently, gamma oscillations provide a dynamic “geometric scaffold” safeguarding perceptual fidelity against energy fluctuations. This framework unifies representational geometry with oscillatory dynamics, offering a feature-specific perspective on how the cortex stabilizes sensory information.

## Abstract (web summary)

Geometric analysis shows that amplitude modulations embedded within cortical gamma waveforms construct a stable, low-dimensional manifold that supports scale-invariant visual color representations in the primate inferior temporal cortex.

## Introduction

A central challenge in neuroscience is understanding how the brain generates stable perceptual representations from dynamic neural activity. While early work established feature tuning in individual neurons^1^, recent focus has shifted to the collective activity of neural populations. From this population-level perspective, two major conceptual frameworks have emerged in parallel. The neural manifold framework has provided a powerful geometric perspective, revealing how population firing rates (i.e., the magnitude of neural activity) evolve within a low-dimensional structure to represent the ‘what’ of a computation^2–4^. Concurrently, a long-standing hypothesis suggests that neural oscillations, typically studied through field potentials, serve as a fundamental dynamic mechanism for the ‘how’ of computation, such as information routing and binding via synchronization^5–7^. However, these two dominant frameworks—one focused on geometric structure (from rates) and the other on dynamic mechanism (from oscillations)—have remained conceptually disconnected, largely by operating on different signals. It remains unknown whether the geometric principles of manifold analysis, typically applied to firing rates, could also describe the collective dynamics of neural oscillations themselves. This gap exists largely because standard manifold techniques were developed for non-negative, time-averaged firing rates, whereas neural oscillations manifest as continuously fluctuating, zero-mean waveforms, whose population-level geometry cannot be captured by static spatial offsets. Bridging this gap, by defining a geometric framework for population-level oscillatory activity, is essential for a comprehensive understanding of neural computation.

The primate inferior temporal cortex (ITC) offers an ideal neural substrate to investigate this unification. As the apex of the ventral visual stream, ITC is the cortical stage where stable and invariant representations of complex object features, such as color and shape, are thought to be fully formed^8,9^. Computationally, the function of the ventral stream is often conceptualized as the “untangling” of object representations into a linearly separable format—a process that implies the formation of a well-structured neural manifold^10,11^. Mechanistically, ITC exhibits prominent gamma-band oscillations that are strongly modulated during visual processing^12^. However, how these oscillatory dynamics specifically within ITC give rise to the area’s hallmark function—the formation of stable, feature-specific representations—remains poorly understood. This convergence of a critical representational function and a key dynamic signature makes ITC the quintessential arena to test the hypothesis that the stable geometry of a sensory code is instantiated and shaped by the underlying oscillatory dynamics of the neural population.

To test this hypothesis, we recorded large-scale electrocorticography (ECoG) signals from the ITC of macaques viewing stimuli differing in color and shape. While our analysis confirmed that time-averaged gamma power carries feature information, we aimed to move beyond static measurements to investigate the geometry of fine-timescale temporal coordination^13^. Recognizing that induced gamma oscillations represent information dynamically rather than through static phase locking, we directly quantified the macroscopic state-space geometry of these amplitude-modulated (AM) waveforms, introducing the concept of an “oscillatory manifold.” This framework provides a direct bridge between representational geometry and underlying dynamics. We found that neural trajectories for different colors were segregated within the gamma band via distinct amplitude modulations of a shared oscillatory carrier. While the absolute spatial separation of these trajectories decayed rapidly following an initial stimulus-locked transient, their color-specific geometric structure remained invariant to the overall scale of the trajectory within the state space. Furthermore, we confirmed that this geometric stability is feature-specific; while stimulus shape was decodable from gamma-band power, it lacked a stable oscillatory geometric structure. By focusing primarily on the dynamics of isoluminant color representations, we characterized the spatiotemporal evolution of this framework. Consequently, network-wide gamma synchronization provides a specialized, “geometric scaffold” to stably transmit specific features in a noisy environment.

## Results

### Gamma-band power in ITC preferentially encodes stimulus color over shape

**Fig. 1:**
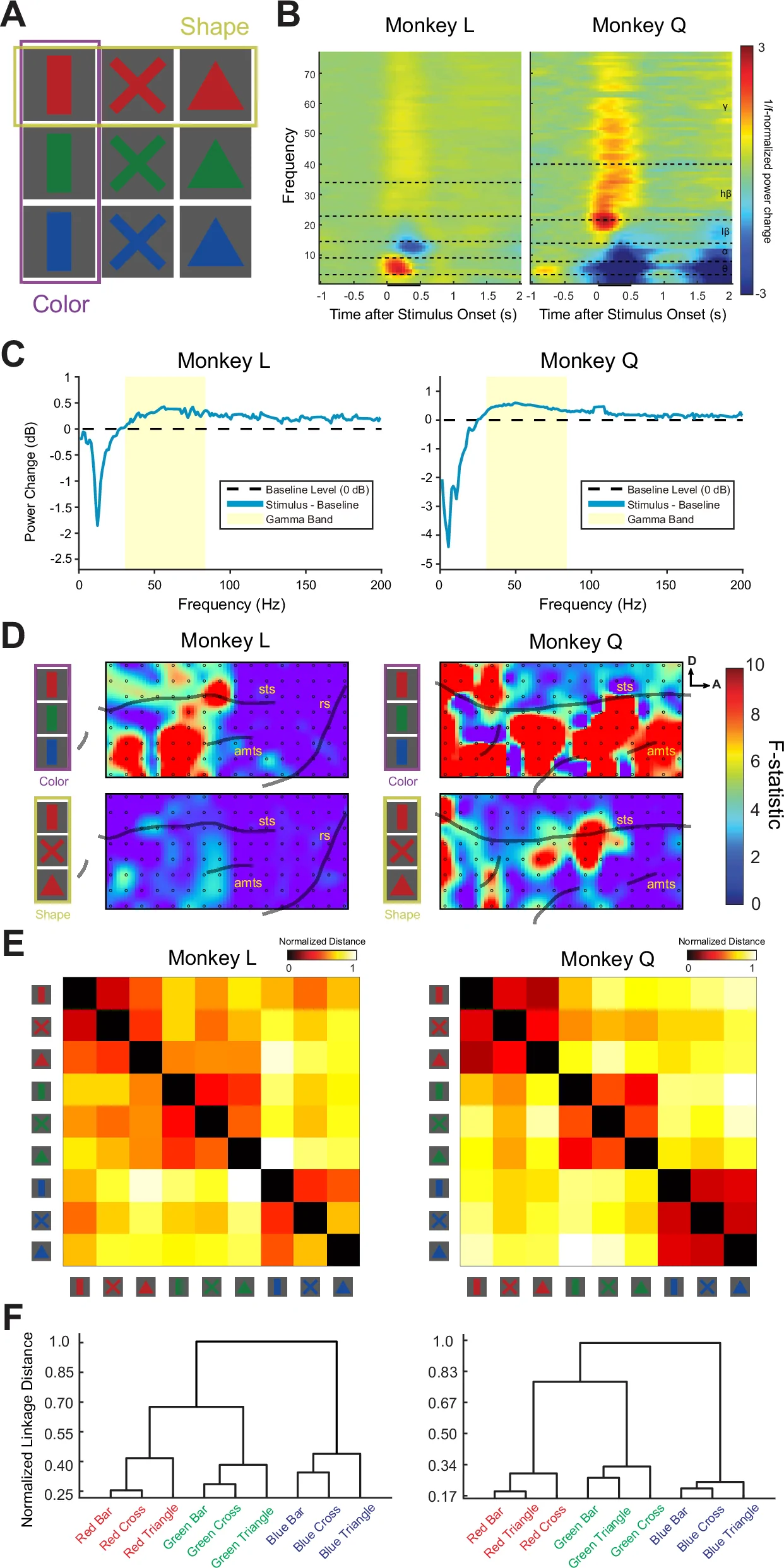
Gamma-band power in ITC preferentially encodes stimulus color information. **A** Visual stimuli. Nine stimuli, derived from a 3 × 3 factorial combination of isoluminant color (red, green, blue) and shape (bar, cross, triangle), were presented. **B** Time-frequency representations of stimulus-evoked power changes. Spectrograms show the difference in 1/f-normalized power from the pre-stimulus baseline averaged across all ECoG channels in Monkey L and Monkey Q. Stimulus onset is at *t* = 0. Frequency bands (dashed lines) are defined guided by a hierarchical clustering analysis. **C** Stimulus-evoked power change from baseline. The cyan line displays the power change (in dB) during the stimulus window (0.1–0.5 s) relative to the pre-stimulus baseline (–0.8 to –0.2 s). The functionally defined gamma band (yellow shaded region) exhibits a band-limited power elevation. **D** Statistical maps of feature selectivity. Color indicates the *F*-statistic from a one-way ANOVA on the mean gamma-band power (0–500 ms post-stimulus) for color and shape, respectively. Black dots indicate individual ECoG electrodes. Major sulci are overlaid: STS (superior temporal sulcus), AMTS (anterior middle temporal sulcus), and RS (rhinal sulcus). **E** Representational dissimilarity matrices (RDMs) for the nine stimulus conditions. Dissimilarity was quantified as the Euclidean distance between population response vectors (derived from the 128-channel 1/f-normalized gamma power) and subsequently max-normalized to a bounded range of [0, 1]. **F** Hierarchical clustering dendrograms illustrating the representational similarity structure of the nine stimuli. The trees were constructed by applying Ward’s linkage method to the normalized distance matrices in **E**.

To investigate how basic visual features are represented in the primate inferior temporal cortex (ITC), we recorded ECoG signals from two macaques (Monkey L and Monkey Q) performing a passive viewing task. The stimuli varied factorially across two features: color (red, green, blue) and shape (bar, cross, triangle) (Fig. 1A). Across the broad coverage of the lateral and ventral ITC (Supplementary Fig. 1A), stimulus presentation consistently evoked two prominent effects: a strong power increase in the gamma frequency range and a concurrent power suppression in the alpha band (Fig. 1B). Examination of the raw power spectral density (PSD) confirmed that this gamma response manifested as a band-limited power elevation over the typical 1/f background decay (Supplementary Fig. 1B). The specific frequency bands, including the gamma band ( ~ 40–80 Hz), were defined in a data-informed manner based on hierarchical clustering of temporal profiles (Supplementary Fig. 2). To clearly visualize the temporal dynamics of this stimulus-evoked response, we computed the power change in decibels (dB) from the pre-stimulus baseline (Fig. 1C). Furthermore, while the absolute power intensity fluctuated across specific color conditions, consistent power elevations within this same gamma band were observed across all individual stimulus conditions (Supplementary Fig. 3).

To test whether this prominent gamma response carried feature-specific information, we performed a one-way ANOVA on the mean gamma-band power at each electrode. This analysis revealed a clear and consistent asymmetry in the representation of color and shape. In both monkeys, large, contiguous regions of the ITC showed significant selectivity for stimulus color (Fig. 1D, effect of color; one-way ANOVA; *p* < 0.05, FDR corrected). In contrast, selectivity for stimulus shape was spatially more restricted, localized to isolated clusters of electrodes showing significant modulation (Fig. 1D, effect of shape; *p* < 0.05, FDR corrected).

To examine how this representational asymmetry was organized at the population level, we investigated the representational geometry using Representational Dissimilarity Matrices (RDMs). For both monkeys, the RDMs based on gamma-band power exhibited a clear 3 × 3 block-diagonal structure, demonstrating that stimuli of the same color evoked highly similar population activity patterns regardless of shape (Fig. 1E). Hierarchical clustering of these matrices confirmed that the primary branches of the representational hierarchy were organized exclusively by color, with shape identity forming smaller, secondary sub-clusters (Fig. 1F). Taken together, these convergent analyses demonstrate that gamma-band power in the primate ITC provides a widespread population representation dominated by stimulus color, while significant shape representations remain confined to sparse, localized electrode clusters at this macroscopic scale.

### Spatiotemporal stability of the color code contrasts with subject-specific shape representations

**Fig. 2:**
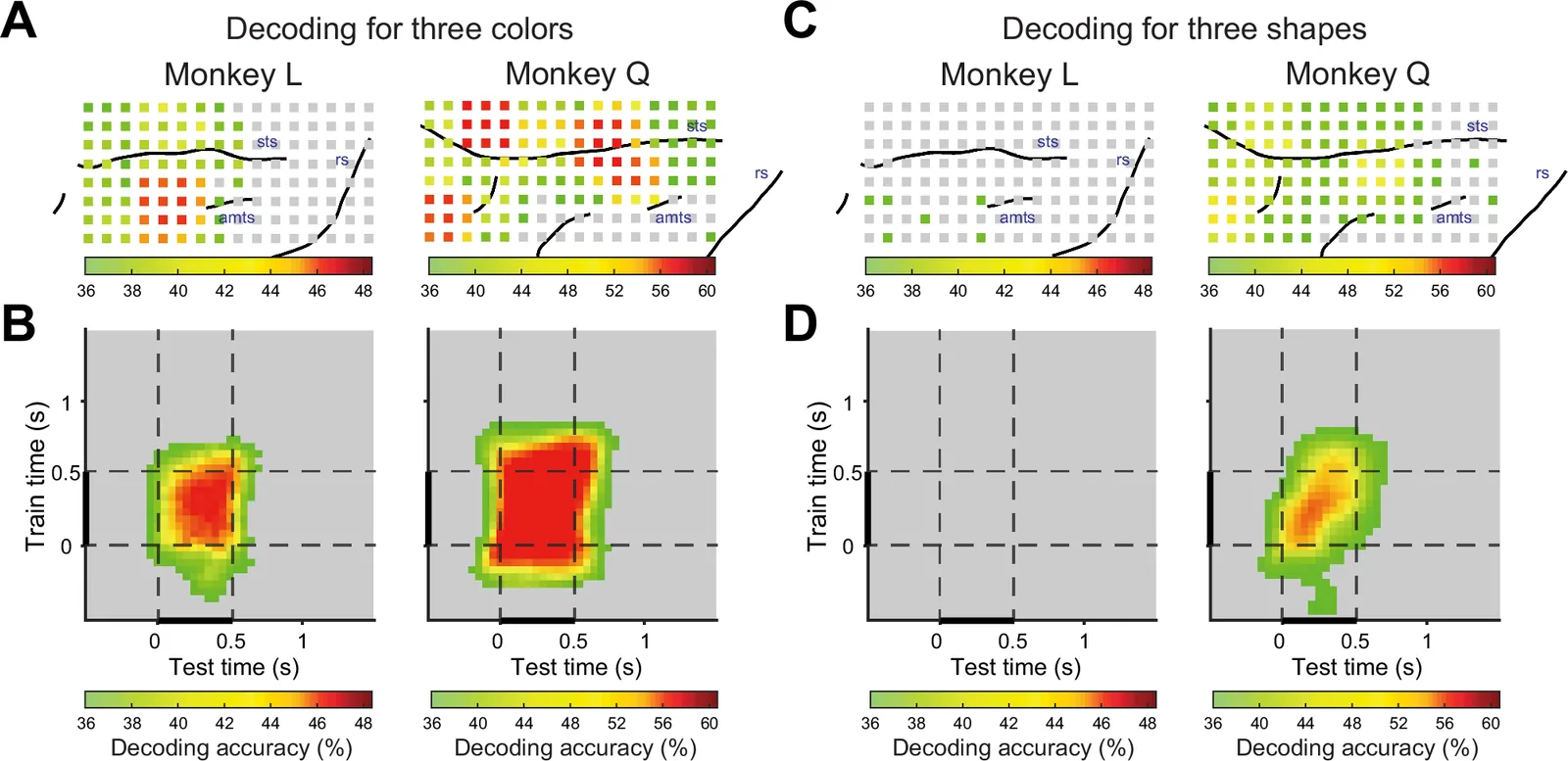
Spatiotemporal stability of the color code contrasts with subject-specific shape decoding. **A** Spatial maps of color decoding accuracy from gamma-band power (0–500 ms post-stimulus) derived from a searchlight analysis. Major sulci are overlaid as black lines: STS, AMTS, and RS. Colored regions indicate channels with significant decoding accuracy (permutation test; *p* < 0.05, FDR corrected), whereas non-significant channels are shown in gray. **B** Temporal generalization matrices for color decoding from all channels. A classifier was trained at each time point (y-axis) and tested at every other time point (x-axis). The stimulus presentation window (0–0.5 s) is indicated by black bars. Colored areas indicate significant cross-decoding accuracy (permutation test; *p* < 0.05, FDR corrected), and non-significant points are shown in gray. **C** Spatial maps of shape decoding accuracy, generated using the same analysis as in **A**. **D** Temporal generalization matrices for shape decoding, generated using the same analysis as in **C**.

Having established that gamma-band power carries strong information about color, we employed multivariate decoding to resolve its spatiotemporal structure. First, a searchlight analysis mapped the spatial distribution of color information, revealing distributed clusters of high decoding accuracy across the ITC of both monkeys (Fig. 2A; linear SVM, permutation test; *p* < 0.05, FDR corrected). To explicitly test the temporal stability of this representation, we performed a cross-temporal decoding analysis. The resulting generalization matrices showed sustained cross-temporal decoding accuracy throughout the stimulus presentation (Fig. 2B; permutation test; *p* < 0.05, FDR corrected). This stable cross-temporal performance demonstrates that the macroscopic neural code for color maintains a highly stable representational format over the stimulus duration. Together, these results indicate that gamma-band power provides a spatially distributed and temporally stable representation of color.

In contrast to the consistent representation for color, the macroscopic gamma-band code for shape exhibited distinct, subject-specific characteristics. In the spatial domain, Monkey Q exhibited a broader distribution of significant shape decoding, whereas this information was restricted to a few isolated channels in Monkey L (Fig. 2C). Cross-temporal analysis similarly reflected this individual difference, but also revealed a distinct temporal profile: while Monkey L exhibited minimal shape decoding, Monkey Q showed significant accuracy. Importantly, unlike the stable, square-like cross-temporal generalization observed for color (Fig. 2B), Monkey Q’s shape decoding was predominantly concentrated along the diagonal, suggesting a more dynamic and less stable neural representation (Fig. 2D). To further validate the reliability of this shape signal against potential color confounds, we performed a stratified analysis (decoding shape within fixed color conditions). This analysis corroborated the standard results, showing that significant shape decoding accuracy in Monkey Q persisted even under these strict controls (Supplementary Fig. 4). These findings demonstrate that gamma-band power in the ITC encodes shape information, particularly in specific subjects or subregions, albeit at a lower magnitude than color. However, while multivariate decoding establishes the presence of this feature-specific information, it does not reveal the underlying representational format. To understand how color and shape are structurally organized within the population state space, we next examined the geometric properties of these macroscopic neural codes.

### A canonical, color-dominated coding geometry characterizes the gamma-band power envelope

**Fig. 3:**
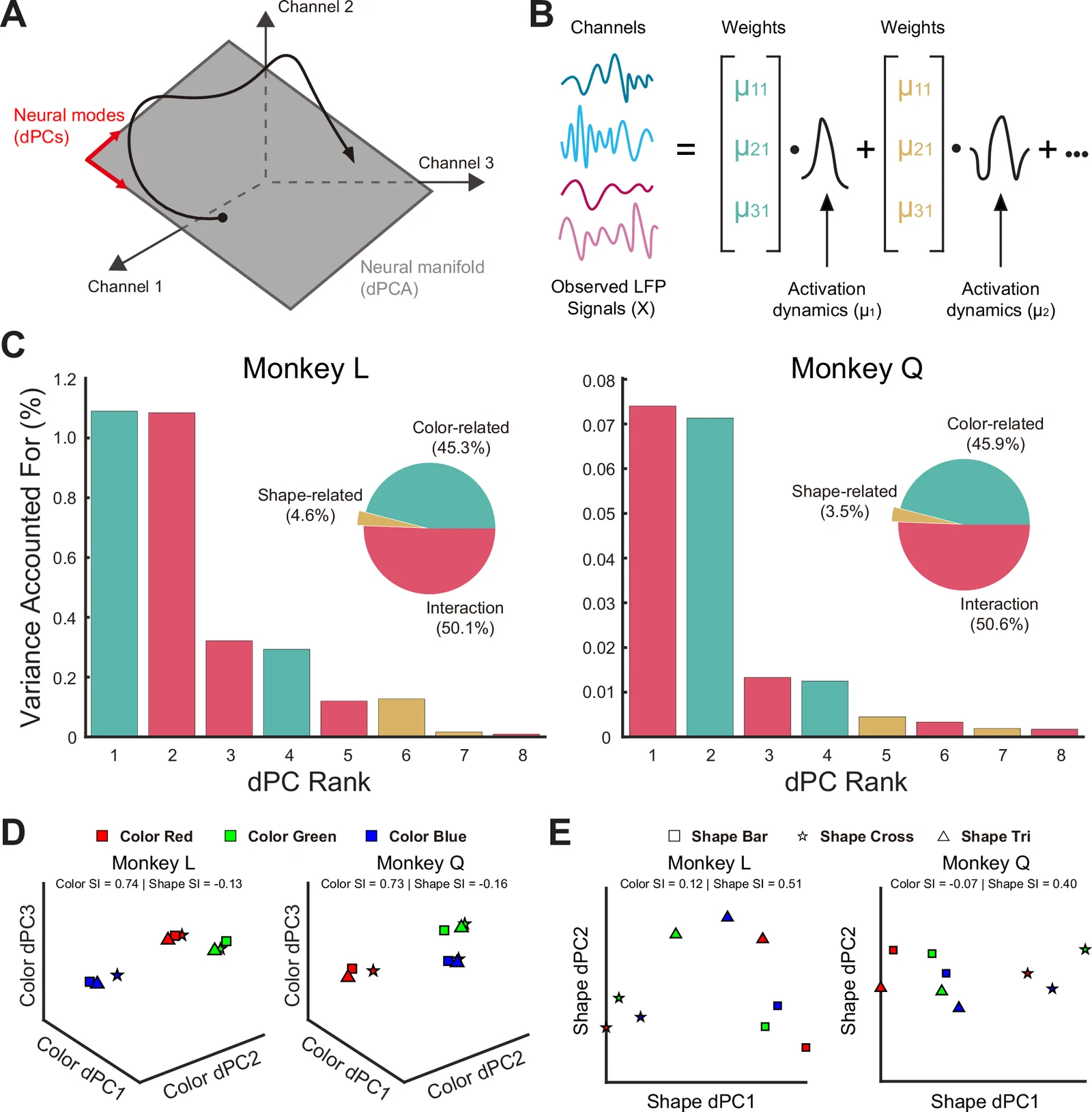
Global coding geometry of the gamma-band power envelope is dominated by color dynamics. **A** Conceptual schematic of the population state space. The time-varying activity from all 128 channels traces a trajectory (black line) through a high-dimensional state space. This activity is often constrained to a low-dimensional “neural manifold” (grey plane), which represents the intrinsic patterns of population co-modulation. **B** Conceptual schematic of dPCA. In this analysis, the observed gamma-band power envelope (left) is modeled as a linear sum of a smaller set of underlying components. The dPCA method finds these components and “demixes” them, separating their time courses (labeled “Activation dynamics (μ1, μ2…)”) based on which task parameter (e.g., ‘Color’, ‘Shape’, or ‘Time’) they relate to. **C** Quantification of task-relevant variance by dPCA for the two monkeys. Bar plots show the percentage of variance accounted for (VAF) by the top 8 demixed principal components (dPCs). Each bar is colored according to its marginalized identity (Color-related: teal; Shape-related: yellow; Interaction: red). Pie charts illustrate the relative percentage of the total task-modulated variance (i.e., isolating the condition-dependent signal by excluding the large condition-independent component) attributable to all Color, Shape, or Interaction components combined. **D** Projection of the time-averaged neural states (9 conditions) onto the dominant “Color-related” subspace identified by dPCA. Each point represents one condition. Marker shape indicates the stimulus shape (Square = Bar, Star = Cross, Triangle = Triangle), and marker color indicates the stimulus color (Red, Green, Blue). **E** Projection of the population activity onto the shape-marginalized subspace. Data were projected onto the top two dPCs marginalized exclusively for shape (which account for < 5% of total variance). Marker conventions are the same as in **D**.

To address whether the decodable information for color and shape emerges from a similar population-level structure, we characterized the representational geometry of these features in the neural state space. While our previous analyses confirmed that gamma-band power carries feature-specific information, they do not elucidate how this code is structured dynamically. For instance, is the color code a static, persistent signal (a ‘Color’ component), or is it a dynamic, time-varying pattern (a ‘Color × Time’ interaction)? To separate these possibilities, we applied demixed Principal Component Analysis (dPCA) to the gamma-band power envelope. This dimensionality reduction method separates population-wide activity into distinct components related to static color, static shape, time, or their dynamic interactions (Figs. 3A, 3B), allowing us to investigate the detailed organizational structure of the code.

This analysis revealed a canonical coding geometry consistent across both monkeys. Of the total task-modulated variance, the population activity was dominated by ‘Color-related’ ( ~ 45%) and ‘Color × Time Interaction’ ( ~ 50%) components (Fig. 3C). In contrast, the ‘Shape-related’ component accounted for a minimal fraction of the variance ( < 5%). To investigate the biological origin of the high variance in the Interaction component, we examined its temporal dynamics. As expected for stimulus-induced responses, the Interaction dPCs captured the non-stationary evolution of power envelopes (e.g., initial transients and subsequent adaptation). Despite these amplitude fluctuations, the inter-color geometric distance remained constant throughout the stimulus presentation, providing a stable geometric structure (Supplementary Fig. 5).

To functionally validate this structure, we projected the neural states onto the dominant dPCA subspaces and quantified their geometric clustering using a distance-based Separation Index. This confirmed that the high-variance Color subspace robustly organized the data by color (Fig. 3D). To rigorously address whether the lack of shape separation was merely an artifact of projecting into color-dominated axes, we performed a control projection exclusively onto the Shape-specific subspace. Notably, the dPCA algorithm identified only two shape-related components, which collectively accounted for < 5% of the total variance (Fig. 3E). Even within these optimized axes, the clustering of shape conditions was substantially weaker and inconsistent across subjects. We further quantified the robustness of these manifolds against a permutation-based chance level. Across both subjects, the Color manifold exhibited a highly deterministic geometric organization, significantly exceeding the chance level (permutation test; *p* < 0.001 for both monkeys). In contrast, the Shape manifold lacked comparable statistical robustness; its separation was only weakly significant in one subject and indistinguishable from chance in the other (Monkey L: *p* < 0.05; Monkey Q: n.s.).

This finding highlights a critical distinction between the presence of decodable information and its global structural organization. While Monkey Q exhibited robust shape decoding (Fig. 2), dPCA reveals that this information does not organize into a dominant, high-variance component at the macroscopic level. Because the power envelope primarily captures relatively slow, broad amplitude modulations, it remains possible that feature-specific geometries are encoded within the fine-timescale temporal coordination of the neural population. To test this hypothesis, we next extended our state-space analysis directly to the high-dimensional oscillatory waveforms.

### Amplitude modulation of gamma oscillations forms geometrically stable and reproducible color trajectories

**Fig. 4:**
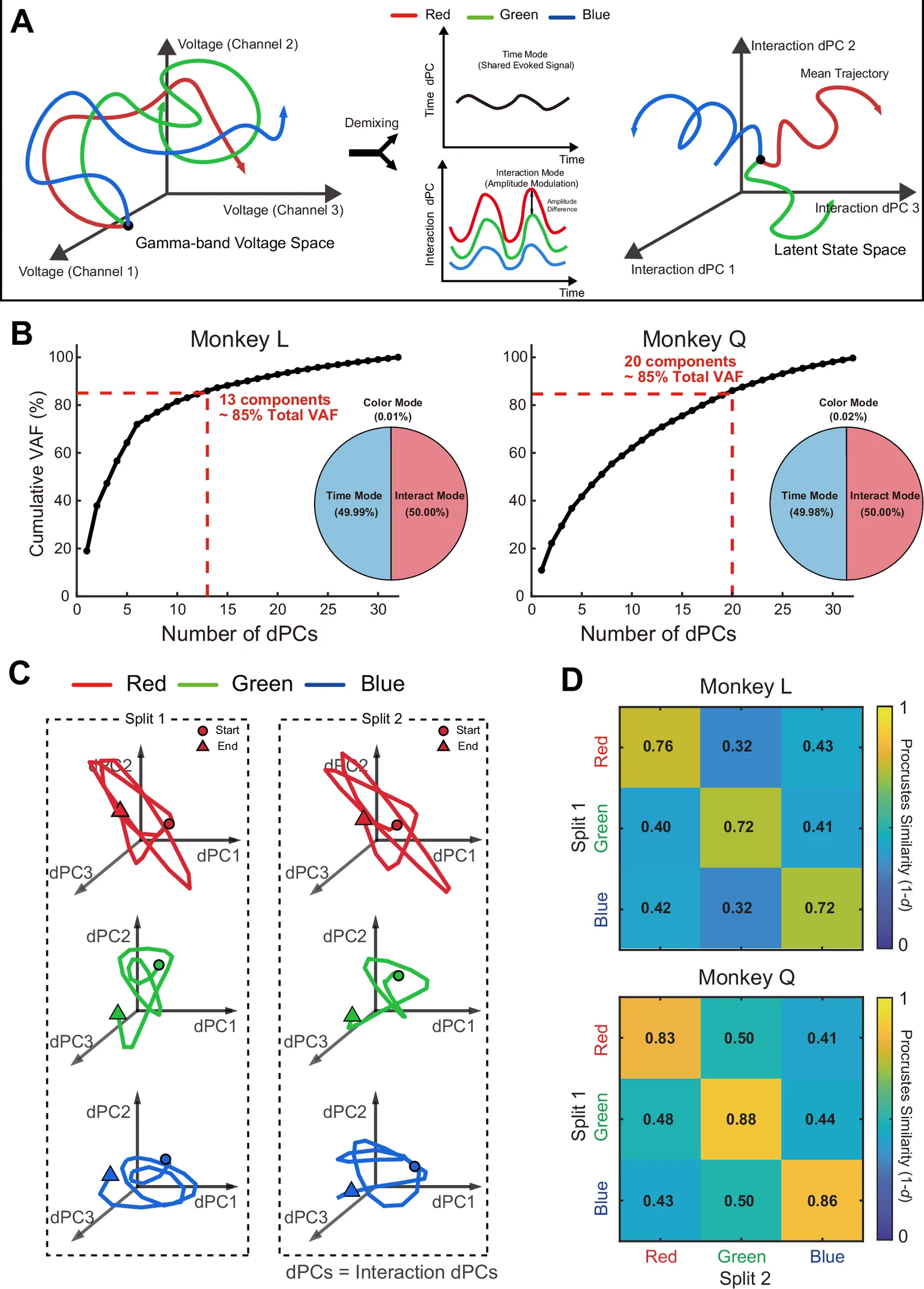
Amplitude modulation of gamma oscillations forms geometrically stable and reproducible color trajectories. **A** Methodological pipeline for the extraction of the oscillatory manifold. The diagram illustrates the sequential stages: gamma-band (35–80 Hz) oscillatory waveforms represented as overlapping trajectories within the multi-channel voltage space; decomposition of these signals by the dPCA algorithm into condition-independent temporal dynamics (Time Mode) and color-specific modulations (Interaction Mode); and the projection of the resulting condition-specific trajectories (Red, Green, Blue) onto the top three Interaction dPCs. **B** dPCA variance decomposition of gamma-band waveforms. Main plots show the cumulative variance explained (VAF) by the dPCs for Monkey L and Monkey Q. Insets (pie charts) detail the breakdown of task-related and condition-independent variance. Applying dPCA to waveforms reveals that static ‘Color’ components account for ~0% of the variance. Instead, the variance is predominantly divided between the condition-independent ‘Time’ component ( ~ 50%) and the condition-specific ‘Interaction’ component ( ~ 50%). **C** Cross-validated visualization of 3D oscillatory trajectories. Trial-averaged waveforms for Monkey L during the stimulus-evoked energy peak (a representative 100 ms window covering approximately 3–4 gamma cycles) are projected onto the top three Interaction dPCs. Independent data splits (Split 1 and Split 2) are plotted separately. Open circles and filled triangles denote the start and end of the trajectories, respectively. **D** Quantitative evaluation of geometric stability across the full stimulus duration (0–500 ms). Procrustes similarity matrices for both monkeys show the cross-validated Procrustes similarity (1 – *d*, where *d* is the Procrustes distance) between high-dimensional trajectories from the Train Set (Split 1) and the Test Set (Split 2). The within-condition trajectory similarity significantly exceeds the noise baseline (permutation test; *p* < 0.001).

To uncover the representational format hidden within the fine temporal structure, we shifted our analysis from the power envelope to the high-dimensional oscillatory waveforms. Because oscillatory waveforms physically fluctuate around zero, stimulus information cannot be represented as static offsets, but must be dynamically encoded through amplitude modulations (AM) of the underlying oscillatory carrier. To explicitly isolate this condition-specific amplitude scaling from the shared high-frequency carrier, we established a methodological pipeline applying dPCA directly to the continuous gamma-band signals (Fig. 4A). To establish the fundamental geometric framework of this oscillatory manifold, we first focused on the robust color representations. In contrast to the power envelope, applying dPCA to the raw waveforms revealed that static ‘Color’ components accounted for negligible variance ( ~ 0%). Instead, the population variance was evenly split between the condition-independent ‘Time’ component ( ~ 50%), capturing the shared stimulus-evoked oscillatory dynamics, and the dynamic ‘Interaction’ component ( ~ 50%), which captured almost all of the condition-specific, task-related information (Fig. 4B). This variance decomposition confirms that in the waveform domain, color information is entirely implemented as color-specific amplitude modulations riding on top of shared population patterns.

We next examined whether these low-dimensional state-space trajectories, derived directly from continuous oscillatory waveforms, could be driven by mathematical artifacts of the dimensionality reduction pipeline. Recent theoretical work cautions that the orthogonality constraints implicit in algorithms like PCA or dPCA can project band-pass filtered noise into artifactual, purely sinusoidal “phantom oscillations”^14,15^. To rule out this possibility, we isolated the instantaneous amplitude modulation (AM) envelopes from the dominant Interaction trajectory (dPC1) using the Hilbert transform and evaluated their power spectral density (PSD) (Supplementary Fig. 6). If the dynamic trajectories inherent in these demixed components were algorithmically imposed phantom oscillations, their amplitude envelopes would manifest as periodic interference beats, resulting in sharp, narrow-band harmonic spikes in the modulation spectrum. Instead, our empirical data yielded spectra devoid of such harmonic artifacts, exhibiting an aperiodic 1/f-like decay with low-frequency elevations ( < 20 Hz). This spectral signature establishes that the structuring of our oscillatory manifold reflects genuine, stimulus-evoked transient physiological dynamics rather than artificial algorithmic periodicity^16^.

Having thus validated the physiological authenticity of this dynamic AM representation, our critical question was whether these trajectories construct a reproducible, multidimensional geometric scaffold. To test the topological rigidity of these trajectories, we projected waveforms from independent data splits onto the top Interaction dPCs. Visually, the trajectories revealed a conservation of geometric shape (e.g., forming consistent loops) across independent trials (Fig. 4C). We quantified this using Procrustes analysis across the full stimulus duration (0–500 ms). Because Procrustes is mathematically scale-invariant, it allowed us to extract the topological shape of these trajectories independent of absolute amplitude fluctuations. This analysis confirmed that trajectories of the same color shared highly reproducible multidimensional shapes (Fig. 4D, diagonal elements of the similarity matrices). Permutation testing against time-scrambled surrogate data confirmed that this geometric stability significantly exceeded chance, demonstrating that AM trajectories maintain a deterministic topological order rather than reflecting random noise fluctuations (permutation test; *p* < 0.001). Furthermore, this robust topological conservation remained significantly above chance across both the early stimulus-evoked transient and the subsequent sustained phase (Supplementary Fig. 7), indicating a structurally rigid manifold over time.

Finally, to ensure this stable geometric structure is specific to the gamma band and not a broadband artifact, we applied the identical Procrustes analysis across other major frequency bands (theta to high-beta) as well as narrower low- and high-gamma sub-bands (Supplementary Fig. 8). While a slight diagonal structure was visually apparent in the high-beta band, clear geometric stability was qualitatively restricted to the low- and high-gamma ranges. Taken together, these findings demonstrate that color is implemented as a stable, amplitude-modulated geometric code specifically within high-frequency oscillatory dynamics.

### Absolute geometric separation of color manifolds exhibits stimulus-locked dynamic expansion

**Fig. 5:**
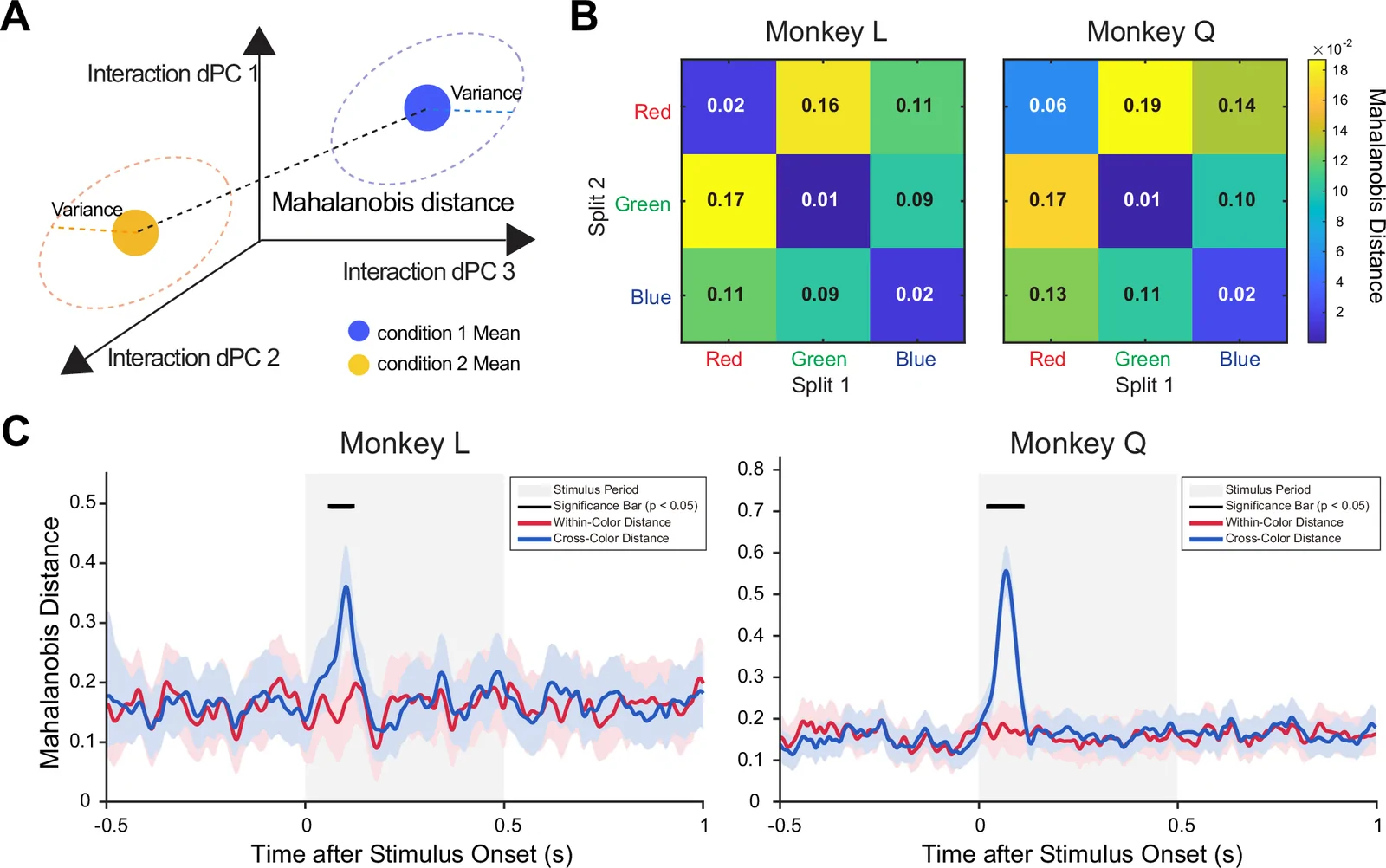
Absolute geometric separation of color manifolds is driven by a highly transient, stimulus-locked dynamic expansion. **A** Schematic illustration of the Mahalanobis distance metric. Unlike simple Euclidean distance, the Mahalanobis distance accounts for the high-dimensional covariance structure (“noise cloud,” represented by dashed ellipses) of the neural population. This provides a unit-less measure of the spatial separability between trajectory centroids using the covariance structure estimated across the full stimulus window (0–500 ms). **B** Static geometric separation of color representations. Pairwise Mahalanobis distance matrices averaged across the full stimulus window (0–500 ms) for Monkey L and Monkey Q, respectively. Rows and columns represent trajectory averages from independent data splits. **C** Time-resolved dynamic manifold separation. Curves show the instantaneous trajectory distance (Gaussian smoothed) between different colors (“Cross-Color,” blue) and within the same color (“Within-Color,” red) over time. Shaded areas represent empirical 95% confidence intervals derived from the 2.5th and 97.5th percentiles of the bootstrap distribution, and the black horizontal bar at the top indicates time points where the Cross-Color distance is significantly greater than the Within-Color distance (cluster-based permutation test; *p* < 0.05).

While our prior Procrustes analysis established that neural trajectories for a given color are highly reproducible, its inherent scale-invariance means it primarily captures topological shape similarity rather than the absolute physical distinctness of representations relative to neural noise. To strictly quantify the separability of this code, we calculated the pairwise Mahalanobis distances between color conditions using the waveforms projected onto the Interaction dPCs. This metric normalizes the state-space distance by the covariance structure estimated across the full stimulus window (0–500 ms) (Fig. 5A). Averaging the trajectories across the full stimulus window (0–500 ms) revealed a robust and conserved static geometric structure in both subjects (Fig. 5B): the distances between identical color conditions (diagonal elements of the distance matrices) were consistently low, while the distances between different colors (off-diagonal) were uniformly and substantially enlarged. This confirms that, on average, color-specific AM trajectories occupy distinct, non-overlapping geometric “territories” on the oscillatory manifold.

We next examined the fine-timescale temporal evolution of this separation. To capture the true spatial distance between oscillating manifolds while circumventing the temporal smearing inherent to long spectral windows, we computed the instantaneous trajectory distance (subsequently Gaussian smoothed). A clear temporal dynamic emerged: while both within- and cross-color measures were at baseline levels prior to stimulus onset, the cross-color distance exhibited a sharp, stimulus-locked elevation rapidly following stimulus presentation (*t* = 0 s). This geometric expansion was transient. In both subjects, the absolute separation peaked and was statistically significant during the early evoked phase ( ~ 0–150 ms), before regressing toward baseline levels as the trial progressed (cluster-based permutation test; *p* < 0.05). Concurrently, the within-color distance remained suppressed and stable throughout (Fig. 5C). This transient separation contrasts with the sustained geometric rigidity observed in our Procrustes analysis (Fig. 4 and Supplementary Fig. 7). As the initial gamma amplitude decays, absolute state-space distances shrink, but the scale-invariant topological ‘shape’ of the trajectories is conserved. Thus, the manifold’s geometric structure persists despite dynamic fluctuations in signal amplitude.

### Shape representations lack a stable geometric structure in the oscillatory manifold

**Fig. 6:**
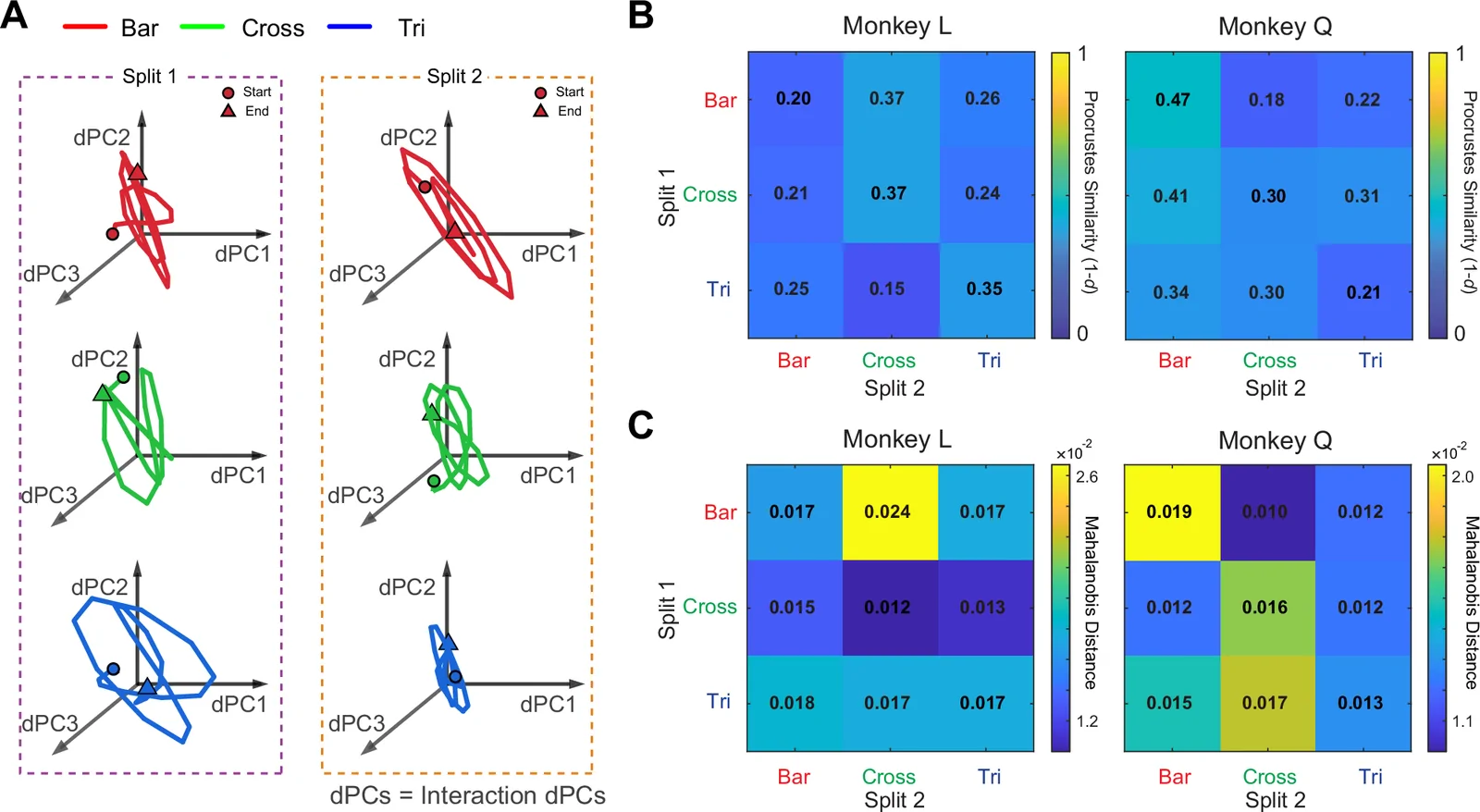
Absence of a stable geometric structure for shape representations in the oscillatory manifold. **A** State-space projections of shape-specific population activity (a representative 100 ms window covering approximately 3–4 gamma cycles) for Monkey Q. Trajectories for three shape conditions (Bar, Cross, Triangle) are projected onto the top three Interaction dPCs. Columns show trajectories derived from the training data (Split 1) and test data (Split 2). Same conventions as Fig. 4C. **B** Cross-validated Procrustes similarity matrices for Monkey L and Monkey Q. The matrices quantify the geometric similarity between shape trajectories in Split 1 and Split 2. Same conventions as Fig. 4D. **C** Matrix of pairwise Mahalanobis distances between shape centroids averaged over the stimulus period (0–500 ms). Values indicate pairwise distances in the oscillatory state space. Same conventions as Fig. 5B.

Our results thus far indicate that color information is encoded via a stable, geometrically organized trajectory within the gamma oscillatory manifold. To determine whether this macroscopic geometric stability generalizes to other features, we examined the representation of stimulus shape. To ensure a direct comparison with our color manifold analysis, we projected the shape-specific population activity onto the top Interaction dPCs (Fig. 6A). In contrast to the distinct manifolds observed for color (Fig. 4C), the state-space projections for the three shape conditions did not exhibit obvious spatial segregation.

To formally quantify this lack of geometric structure, we first computed the cross-validated Procrustes similarity matrices. Unlike the prominent diagonal structure observed for color, these matrices (Fig. 6B) appeared uniform. The within-shape trajectory similarities (diagonal elements) were not reproducible across independent data splits. We next evaluated the absolute spatial separation between conditions using pairwise Mahalanobis distance matrices (Fig. 6C). Crucially, the distances between different shape centroids were virtually indistinguishable from those for the same shape condition. This minimal distance, coupled with a lack of relative spatial segregation, indicates that the shape representations failed to form distinct topological clusters in the state space, remaining effectively indistinguishable from intrinsic neural noise.

Notably, our earlier decoding analysis demonstrated that shape information is present and decodable in the gamma-band power, particularly in Monkey Q (Fig. 2D). The absence of a stable shape manifold in these identical signals establishes a functional dissociation. This dissociation rules out the “signal-to-noise ratio” (SNR) confound, indicating that the lack of a shape manifold is not merely due to an absence of shape-related gamma energy, but rather reflects a distinct macroscopic coding format for shape compared to color in the ITC.

## Discussion

A fundamental challenge in systems neuroscience has been to reconcile two dominant but conceptually disconnected frameworks: the “neural manifold” perspective, which describes the geometric structure of population activity representing the content (“what”) of computation^2,3^, and the “neural oscillation” perspective, which proposes dynamic mechanisms for the transmission (“how”) of information^5^. In this study, we bridged this gap by analyzing the geometry of oscillatory waveforms—rather than conventional spectral power—using large-scale electrocorticography (ECoG) signals from the primate inferior temporal cortex (ITC). This approach identified an “oscillatory manifold,” a stable, low-dimensional geometric structure embedded within gamma-band oscillations. We found that this manifold reliably encodes color information through amplitude-modulated, geometrically organized trajectories that emerge dynamically upon stimulus presentation. Although stimulus shape was decodable from overall gamma-band power, it lacked the stable oscillatory geometric structure observed for color. In the context of our experimental design, this contrast serves as a methodological control, confirming that the oscillatory manifold is a specific representational format rather than a generic byproduct of neural activation. These findings point to a dynamic coding mechanism that leverages the geometry of neural oscillations to instantiate stable perceptual representations, suggesting that the oscillatory manifold is not a ubiquitous coding format, but a specialized geometric scaffold selectively recruited based on feature topology and functional architecture.

Our identification of the oscillatory manifold represents a conceptual departure from traditional approaches in population neuroscience. Conventional analyses typically separate neural signals into a ‘rate’ or ‘amplitude’ code (estimated via spike counts or power envelopes)^3,4^ and a ‘temporal’ code (characterized by phase synchronization)^17,18^, effectively discarding the rich geometric structure embedded in the continuous waveform itself. By applying dimensionality reduction directly to the population-level ECoG waveforms, we exposed a high-dimensional state space where sensory information is encoded via dynamic trajectories. Critically, this approach allowed us to reveal a fundamental dissociation invisible to conventional spectral analysis: high decodability from static power does not necessarily imply the existence of a stable geometric structure. This aligns with recent theoretical proposals that the brain utilizes the full richness of continuous dynamics for computation^2^, but extends this concept by demonstrating that such dynamics are strictly organized into stable, reproducible manifolds even at the mesoscopic level of local field potentials^13^.

This waveform-based analysis provides mechanistic insight into how sensory information is structurally encoded within high-frequency networks. Our findings (Fig. 4) reveal that the cortex implements this code via a dynamic interaction between a condition-independent carrier oscillation and feature-specific amplitude modulations. This suggests that the ITC leverages gamma oscillations not merely as a generic temporal clock for binding-by-synchrony^6^, but as a multidimensional carrier wave for population vectors—conceptually akin to AM radio communication^19^. In this framework, sensory content is structured into distinct, topologically rigid geometric paths independent of precise spike-timing relative to the oscillatory cycle.

The concept of the oscillatory manifold roots our findings within the broader theoretical context of neural population dynamics. Foundational work, such as the seminal Wilson-Cowan model^20^, established that macroscopic excitatory and inhibitory interactions can be formulated as dynamic trajectories within a phase space. Recent frameworks have extended this state-space perspective from spiking activity^21^ to macroscopic field potentials^22–24^. By utilizing amplitude modulations rather than raw voltage, our approach bridges these domains. It maps the underlying oscillatory population dynamics into a stable geometric format, circumventing the negative values inherent to raw voltage fluctuations. This specific representational format reveals the dual nature of the manifold: it allows the network to maintain a scale-invariant topological shape for stable sensory coding, even as the absolute energy-dependent spatial separation fluctuates over time. Furthermore, analyzing this geometry across different mathematical representations addresses a critical theoretical question regarding dimensionality. The apparent dimensionality of a neural manifold is bounded by the signal representation and the variance partitioned^25^. Estimating the dimensionality of continuous oscillatory waveforms inherently requires a larger basis set to capture fast-fluctuating phase covariance compared to the lower-dimensional dynamics of macroscopic power envelopes. Despite this difference in mathematical basis, the topological segregation of color information remained conserved across both analyses. This consistency aligns with the “strong principle” of neural dimension reduction^25^, where the low-dimensional manifold is not merely a convenient mathematical tool for data visualization, but rather reflects the actual latent variables utilized by neural circuits to operate and compute.

Importantly, the biological specificity of this manifold structure confirms that it is a genuine physiological mechanism rather than a generic property of band-pass filtered signals. Indeed, our control analyses demonstrated that this stable geometry is uniquely confined to the gamma band ( ~ 40–80 Hz) and is absent in lower frequencies (Supplementary Fig. 8). This specificity aligns perfectly with the prominent role of gamma oscillations in local cortical processing^5,26^, while distinguishing our findings from the lower-frequency dynamics typically associated with long-range feedback^27,28^. Thus, the AM-based oscillatory manifold operates as a biologically grounded and highly specialized channel for establishing stable representations within the ventral visual stream.

The highly transient nature of the absolute spatial separation (Fig. 5C) presents a clear functional contrast to the sustained geometric rigidity observed in our Procrustes analysis (Fig. 4). This transient geometric expansion closely aligns with the well-established dynamics of cortical visual processing, which is typically characterized by a powerful initial stimulus-locked transient followed by a sustained, adapted state. The initial surge in Mahalanobis distance likely reflects this strong feedforward sweep of sensory input energy^9,28,29^. However, following this initial activation, the absolute signal amplitude rapidly decays, consistent with cortical adaptation processes^30–32^. This rapid decay in absolute signal energy poses a fundamental computational challenge for maintaining stable perceptual representations. If population codes rely strictly on absolute scalar magnitudes, a global reduction in network activity (e.g., during sensory adaptation) inevitably forces the population state vector toward the origin. Under such low-activity conditions, single-trial neural trajectories could become highly variable, and the representation risks “spectral collapse,” where its primary geometric structure becomes difficult to distinguish from the noise floor^3,33^. Consequently, relying solely on absolute activity levels during sustained, adapted states could compromise the fidelity of the population code.

Our results demonstrate that the cortex resolves this “collapse to the origin” challenge by leveraging the inherent mathematical properties of the oscillatory manifold. While the absolute state-space distance shrinks back toward the noise floor as the initial signal amplitude decays, the underlying multidimensional ‘shape’ of the trajectories is faithfully conserved^34,35^. This topological persistence is possible because the AM code distributes information across the relative amplitude modulation ratios among distributed channels, rather than relying on absolute scalar magnitudes. This scale-invariant mechanism is conceptually linked to divisive normalization, a canonical computation that stabilizes network dynamics by factoring out global fluctuations in signal scale^36^. Indeed, theoretical frameworks have proposed that divisive normalization and neuronal oscillations can complement each other in a unified architecture, preventing signal degradation and ensuring stable neuronal stimulus representations over time^37^. Our findings demonstrate that the gamma oscillation functions as a “dynamic stabilizer” against intrinsic energy fluctuations and sensory adaptation. By anchoring the representation in relative amplitude patterns, it provides a robust topological framework that safeguards perceptual fidelity.

A critical question arising from our results is why a stable geometric scaffold was observed for color but not for shape (Fig. 6). This functional dissociation refutes the potential confound that the oscillatory manifold is merely a mathematical byproduct of high signal-to-noise ratio. Even when projected onto the exact same type of dynamic subspace used for color (i.e., the Interaction dPCs capturing amplitude modulation), the continuous trajectories lacked the reproducible, topological rigidity observed for color. Thus, the emergence of an oscillatory manifold requires more than just decodable information; it represents a specialized geometric architecture.

The most direct explanation for the absence of a distinct shape manifold in our dataset relates to the inherent properties of the visual stimuli. It is important to note that our stimulus set relied on shapes defined by isoluminant color contrast. Previous psychophysical and physiological studies have established that the visual system processes isoluminant shapes less efficiently than luminance-defined contours^38,39^. Therefore, the lack of topological stability for shape (Fig. 6) should be interpreted primarily as a consequence of these specific stimulus conditions, effectively serving as an internal methodological control.

Beyond these stimulus constraints, the most parsimonious anatomical explanation for this dissociation lies in the distinct anatomical scales of functional organization in the ITC. Color-selective neurons are spatially clustered into large, millimeter-scale domains known as “color globs”^40,41^. These macroscopic domains are sufficiently extensive to generate coherent field potentials detectable by our mesoscopic ECoG electrodes. In contrast, shape selectivity is organized into a distributed mosaic of much finer, sub-millimeter columns^8,42^. These columns form a complex network where neural populations with distinct feature selectivities are spatially intermingled. Consequently, the spatially averaged ECoG signal likely cancels out the heterogeneous oscillatory dynamics arising from this highly distributed, fine-grained architecture, masking the localized geometric organization that may exist at the micro-scale.

While our study establishes a geometric framework for population-level oscillatory dynamics, several limitations warrant consideration. First, the mesoscopic resolution of ECoG arrays limits the observation of single-neuron spiking and highly localized micro-columnar computations^43^. High-density microelectrode arrays are required to bridge these macroscopic manifolds with micro-scale cellular dynamics. Second, our reliance on a passive fixation task inherently limits our ability to equate the stability of the neural representation with the continuity of subjective perception. Recent theoretical and empirical frameworks propose that attention and sensory processing are not continuous, but operate via rhythmic sampling—dynamically alternating between “sampling” and “shifting” phases coordinated by slower frequency rhythms such as the theta and alpha bands^44,45^. Therefore, while the oscillatory manifold maintains a topologically rigid structure despite the time-varying nature of its trajectories, it is highly plausible that these representations are subjected to intermittent, rhythmic sampling by downstream cortical networks. Active discrimination paradigms will be necessary to explicitly test how these high-frequency geometric scaffolds are dynamically accessed by top-down attentional rhythms. Third, although our factorial design isolated visual features, the intrinsic psychophysical discriminability of color and shape categories was not equated. Lacking behavioral read-outs of perceptual thresholds, we cannot determine whether lower shape decodability stems from fundamental neural representation differences or the relative difficulty of implicit discrimination. However, our use of the shape condition as a methodological control rests on representational geometry rather than absolute decoding accuracy. As observed in our data (Fig. 2D and Fig. 6), even when shape information is effectively decodable from static gamma power, it lacks the topologically stable trajectory inherent to the color manifold. This confirms that the oscillatory manifold is a specialized geometric format, not merely a byproduct of high signal-to-noise ratio. Fourth, the extent to which these specific oscillatory manifolds generalize to the high-dimensional continuous space of naturalistic objects remains to be determined. Finally, our current evidence linking this geometric scaffold to perceptual stability remains correlational. Establishing causality will require targeted manipulations, such as intracortical microstimulation^46^ or closed-loop perturbation of gamma oscillations^47^, to directly test whether traversing these precise geometric trajectories is strictly necessary for stable conscious perception.

Beyond the coding of specific features, the identification of this scale-invariant geometric scaffold offers critical insights into how the visual system achieves perceptual stability in ecological settings. In natural vision, retinal input is inherently unstable, continuously perturbed by saccades, blinks, and fluctuations in lighting conditions^48,49^. By absorbing this local noise and input variance through its relative amplitude topology, the oscillatory manifold can transform highly volatile sensory signals into enduring cognitive codes, preserving our subjective experience of object features.

In conclusion, our findings suggest that gamma oscillations provide more than just a temporal clock for binding; they construct a stable geometric scaffold for representation. By confining high-dimensional population dynamics to low-dimensional manifolds^3,50^, the cortex ensures that sensory information remains robust against noise and readily accessible to downstream readout^10,51,52^. This “oscillatory manifold” framework thus successfully bridges the gap between neural geometry and oscillatory dynamics, offering a unified perspective on how the brain stabilizes dynamic sensory inputs into coherent cognitive codes.

## Methods

### Subjects and housing

The study was conducted with two adult Japanese monkeys (*Macaca fuscata*), identified as Monkey L (female) and Monkey Q (male), with ages ranging from 4 to 7 years and weights between 5.6 and 7.1 kg. Both animals were sourced from the National BioResource Project (NBRP) “Japanese Monkeys,” a program under the Ministry of Education, Culture, Sports, Science and Technology (MEXT) of Japan. Animals were housed in standard primate cages within an air-conditioned room, maintained on a 14:10-hour light-dark schedule. Environmental enrichment was provided to ensure their well-being, and they received a diet of primate food supplemented with fresh fruits and vegetables. All experimental procedures were performed in accordance with a protocol approved by the Institutional Animal Care and Use Committee of Niigata University (Permission number: 27-184-1). We have complied with all relevant ethical regulations for animal use. The care and use of the animals adhered to the Japanese Act on Welfare and Management of Animals, the MEXT’s Fundamental Guidelines for Proper Conduct of Animal Experiment and Related Activities in Academic Research Institutions, and the National Institutes of Health (NIH) Guide for the Care and Use of Laboratory Animals. The two monkeys used in this study were the same individuals as those reported in our previous publications^53,54^. A sample size of two animals is standard for non-human primate electrophysiology studies, balancing the need to demonstrate reproducibility across subjects with ethical mandates for the reduction of animal use.

### Surgical procedures

Each monkey underwent two separate aseptic surgical procedures: an initial surgery for the implantation of a headpost, followed by a second surgery for the placement of the ECoG array. For both procedures, a consistent protocol for anesthesia and physiological monitoring was used. Anesthesia was induced with an intramuscular injection of ketamine (50 mg/kg) and medetomidine (0.03 mg/kg). Following induction, the animal was intubated and connected to an artificial respirator (A.D.S.1000, Engler Engineering Corp.). An intravenous line was established for the administration of lactated Ringer’s solution and a prophylactic antibiotic, ceftriaxone (100 mg/kg). Throughout the surgery, the animal’s body was kept in a stable position using a vacuum fixing bed (Vacuform, B.u.W. Schmidt GmbH), and core body temperature was maintained at 37°C with an electric heating mat. Vital signs, including oxygen saturation, heart rate, and end-tidal CO2, were continuously monitored (Surgi Vet, Smiths Medical PM Inc.) to precisely regulate the anesthetic depth. For the headpost implantation, the monkey’s head was secured in a stereotactic frame (Narishige). After a local injection of lidocaine, the skin and muscle were retracted to expose the skull. A titanium headpost (Gray Matter Research) was then affixed to the skull using bone screws and dental resin. For the ECoG array implantation, the head was stabilized using a three-point fastening device (Integra Co.) fitted with a custom attachment for macaques. The surgical site was exposed, and a craniotomy and durotomy were performed under an operating microscope (Ophthalmo-Stativ S22, Carl Zeiss Inc.) equipped with a CMOS color camera (TS-CA-130MIII, MeCan Imaging Inc.). After the ECoG array was placed, the dura mater, bone flap, and skin were carefully re-sutured in layers, and any exposed skull was covered with dental resin to conclude the procedure. Post-operative care for both surgeries included a three-day course of the analgesic ketoprofen and a one-week course of antibiotics. These surgical, anesthetic, and post-operative protocols were performed in accordance with the established methodologies validated in our prior study^55^.

### Behavioral task and visual stimuli

All experiments were conducted while the monkeys, seated and head-stabilized in a primate chair, performed a passive viewing task. The visual stimuli consisted of nine unique exemplars derived from a 3 × 3 factorial combination of shape (bar, cross, triangle) and color (red, green, blue), as depicted in Fig. 1A. Stimuli were presented centrally on a dark gray background, subtending approximately 3.7° of visual angle. Each trial was initiated by the monkey holding down a lever, which triggered the appearance of a central fixation point (0.1° square). A trial consisted of the following temporal sequence: a 1.5 s pre-stimulus baseline period, a 0.5 s stimulus presentation, and a 2.0 s post-stimulus delay period. The nine stimuli were presented in a pseudorandom order across trials. Throughout this entire sequence, the animal was required to maintain its gaze within a ± 1.5° window around the fixation point. A break in fixation resulted in the immediate termination of the trial. Upon successful completion of the full sequence, the fixation point dimmed, signaling the monkey to release the lever to receive a liquid reward.

### ECoG data acquisition and electrode localization

To record surface potentials across extensive regions of the inferior temporal cortex (ITC), we designed a custom-made 128-channel (16 × 8 grid) ECoG array covering an area of 37.5 × 17.5 mm^2^. The electrode contacts had 0.5 × 0.5 mm square gold surfaces, exposed from a flexible 20 µm-thick Parylene-C film. The center-to-center distance between adjacent electrodes was 2.5 mm. Electrode impedances were typically 20–50 kΩ at 1 kHz. The ECoG array was aseptically implanted subdurally onto the cortex. The array was implanted over the right hemisphere in Monkey L and the left hemisphere in Monkey Q. A gold reference electrode was also positioned in the subdural space near the ECoG array, with its recording surface facing the dura. Lead wires from the ECoG array and reference electrode were tunneled to and connected with microconnectors (Omnetics, MN) housed within a custom titanium chamber, which was anchored to the skull with dental resin. ECoG signals were acquired using a differential amplifier (PBX, Plexon, TX), band-pass filtered (Butterworth, 0.7–300 Hz), digitized, and stored on a hard-disk drive at a 1 kHz sampling rate.

At the conclusion of all recording experiments, electrode positions were histologically confirmed. Monkeys were administered an overdose of sodium pentobarbital and were transcardially perfused with 4% paraformaldehyde in 0.1 M phosphate buffer (pH 7.4). The brain was extracted with the ECoG array left in situ, encapsulated within the dura mater. Photographs were taken of the cortical surface before and after dura removal. These images were used to precisely align the electrode grid’s position relative to anatomical landmarks, such as the superior temporal sulcus and anterior middle temporal sulcus, confirming coverage of the inferior temporal cortex as shown in Fig. 1B.

### Signal preprocessing

Given the within-subject design, experimenters were not blinded to the conditions during data collection. However, stimulus presentation, reward delivery, and eye-tracking were fully automated. All subsequent preprocessing and statistical analyses were performed using automated scripts to eliminate experimenter bias. All offline data processing and analysis were conducted in MATLAB (MathWorks, MA) using custom scripts, supplemented with functions from the FieldTrip Toolbox^56^. The dataset for each monkey was compiled from three recording sessions, yielding a total of 1,836 successful trials for Monkey L and 2,106 successful trials for Monkey Q. This provided a minimum of 200 trials for each of the nine unique stimulus conditions. The continuous ECoG data were first segmented into epochs from -4.5 s to +5.0 s relative to stimulus onset. Each epoch was then band-pass filtered (0.7–250 Hz) and downsampled to 500 Hz for subsequent analysis. To remove non-neural artifacts such as eye movements and muscle noise, we applied an independent component analysis (ICA), and the artifactual components were projected out of the data^54^. Following artifact removal, the signals were re-referenced using a common average referencing (CAR) scheme; the average signal across all 128 channels was computed at each time point and subtracted from each individual channel’s signal.

### Power-based representational analyses

#### Time-frequency analysis

To analyze the oscillatory content of the ECoG signals, we performed a time-frequency decomposition on the preprocessed data. We calculated the power spectrum for each trial (1–80 Hz) using a complex Morlet wavelet transform (implemented in FieldTrip). To optimize the trade-off between temporal and spectral resolution across different frequencies, the width of the wavelets was set to 7 cycles. This frequency-dependent windowing approach inherently provides higher temporal resolution at higher frequencies (e.g., within the gamma band). The power was estimated in 50 ms steps. To account for the 1/f power-law characteristic, the raw power values were normalized by multiplying them by their corresponding frequency (1/f-normalization). These normalized power values served as the basis for all subsequent power-based analyses (e.g., ANOVA, RDM, and decoding; Figs. 1 and 2), except the dPCA of the power envelope (Fig. 3). For visualization purposes, these values were baseline-corrected relative to the pre-stimulus period (–0.8 to –0.2 s). Specifically, for the time-frequency spectrograms (Fig. 1B and Supplementary Fig. 3), the mean baseline power was subtracted, whereas for the frequency spectrum (Fig. 1C), the power change was calculated in decibels (dB).

#### Frequency band definition

Instead of employing conventional, fixed frequency bands, we defined functionally relevant frequency ranges guided by a hierarchical clustering analysis^57^. Briefly, we first calculated the time-frequency representation (TFR) of the LFP power for each channel. The power profiles across frequencies were then used to compute a dissimilarity matrix based on correlation distance (1 - *r*). We applied agglomerative hierarchical clustering using the average linkage method to this matrix to identify clusters of frequencies that exhibited similar temporal dynamics (see Supplementary Fig. 2). Based on the resulting dendrogram, we determined the precise cutoff boundaries between frequency bands for each subject, ensuring that the defined bands reflected the intrinsic physiological structure of the data rather than arbitrary cutoffs. Once these boundaries were objectively identified, the resulting bands were labeled (e.g., ‘Theta’, ‘Alpha’, ‘Gamma’) based on their spectral overlap with conventional neurophysiological frequency definitions. This procedure was performed independently for each animal. The resulting frequency bands used for subsequent power-based analyses were: 1–4 Hz, 5–9 Hz, 10–14 Hz, 15–22 Hz, 23–32 Hz, and 33–80 Hz for Monkey L; and 1–2 Hz, 3–7 Hz, 8–13 Hz, 14–21 Hz, 22–39 Hz, and 40–80 Hz for Monkey Q.

#### ANOVA for feature selectivity

To generate the feature selectivity maps (Fig. 1D), we performed statistical analyses on the 1/f-normalized power within a specific frequency band. First, for each trial, we computed the mean power for the target frequency band by averaging the power values across all time bins that fell within the stimulus presentation window (0–500 ms post-stimulus). Using this single mean power value (per trial) as the input, we performed two separate one-way ANOVAs at each of the 128 electrodes, using custom MATLAB scripts utilizing the ft_freqstatistics function (FieldTrip Toolbox). The first ANOVA tested for the effect of ‘color’ (collapsing across shapes), and the second tested for the effect of ‘shape’ (collapsing across colors). This procedure yielded an *F*-statistic for both ‘color’ and ‘shape’ at each electrode. The resulting *F*-values were then visualized as topographical maps using the ‘ft_topoplotTFR’ function.

#### Representational similarity analysis and hierarchical clustering of stimulus representations

To investigate the geometric structure of the neural representations, we performed representational similarity analysis (RSA). The analysis was applied to the 1/f-normalized power within a specific frequency band of interest averaged over the stimulus presentation window (0–500 ms). First, we defined the population response vector (neural centroid) for each of the nine stimulus conditions (3 colors × 3 shapes) using the activity across all 128 ECoG channels. To construct the Representational Dissimilarity Matrix (RDM) (Fig. 1E), we computed the Euclidean distance between every possible pair of these nine condition-specific centroids. This resulted in a 9 × 9 matrix where each entry represents the dissimilarity between two specific stimulus representations. To quantify and visualize the structure embedded in the RDM, we subsequently performed hierarchical clustering (Fig. 1F). We applied Ward’s linkage method to the distance matrix to generate a dendrogram, illustrating the hierarchical organization of the nine conditions based on their neural similarity.

#### Searchlight decoding analysis

To map the spatial distribution of feature-specific information across the ITC (Fig. 2A, C), we performed a searchlight decoding analysis^58^. This analysis was applied to the 1/f-normalized power within a specific frequency band of interest. Following the procedures described in our previous study^53^, we defined a “searchlight” for each electrode as a local 3×3 grid consisting of the electrode itself and its eight nearest neighbors (approximate radius of 3.6 mm). For each trial, the power values from these 9 channels, averaged over the stimulus presentation window (0–500 ms), were concatenated to form a single feature vector. We then trained a linear support vector machine (SVM) classifier (LIBLINEAR)^59^ to decode the stimulus condition (e.g., 3 colors or 3 shapes). To ensure unbiased classification, the number of trials for each class was balanced by random subsampling. We evaluated classifier performance using a 10-fold cross-validation procedure, and the average decoding accuracy was assigned to the central electrode of the searchlight. This process was iterated across all electrode locations to generate a topographical map of decoding accuracy.

#### Cross-temporal decoding analysis

To investigate the temporal dynamics and stability of the neural representations (Fig. 2B, D), we performed a cross-temporal decoding analysis^60^, following the procedures described in Tanigawa et al.^53^ This analysis was applied to the 1/f-normalized power within a specific frequency band of interest. At each time point (*t*_train_), we trained a linear SVM classifier^59^ to decode the stimulus condition (e.g., color or shape) using the population activity vector (power across all 128 channels). To ensure unbiased classification, the number of trials for each class was balanced by random subsampling. We employed a 10-fold cross-validation procedure: a classifier trained at time t_train_ was tested on data from every time point (*t*_test_) in the held-out test fold. This process was iterated to generate a temporal generalization matrix (TGM), where each cell (*i, j*) represents the decoding accuracy of a classifier trained at time *i* and tested at time *j*.

### Demixed principal component analysis (dPCA) of power envelope

To characterize the global representational geometry of the population code across the distinct feature dimensions (Fig. 3), we applied dPCA^61^ to the gamma-band power signals.

Preprocessing was performed to isolate the instantaneous power of the gamma oscillation. First, the raw ECoG signal for each trial was band-pass filtered (4th-order Butterworth filter, ~40–80 Hz). We then computed the instantaneous power of the filtered signal using the squared magnitude of the Hilbert transform.

Unlike the spectral analyses in Figs. 1 and 2, the input for this analysis was not 1/f-normalized, as comparisons were restricted to a single frequency band. Instead, to account for baseline variability and standardize the signal scale across trials, the power envelope was z-scored on a per-trial basis. Specifically, for each trial and channel, the activity was normalized by subtracting the mean and dividing by the standard deviation of the pre-stimulus baseline period (–800 to –200 ms). The normalized data were then organized into a high-dimensional tensor of dimensions *N* (128 channels) × *S* (3 shapes) × *C* (3 colors) × *T* (time points within the time-of-interest window). To ensure a balanced design for dPCA, trials were subsampled to match the minimum number of trials across the 9 conditions.

We performed standard dPCA to decompose the population variance into latent components dependent on specific task parameters. Specifically, the neural activity was demixed into marginalizations corresponding to ‘Color’, ‘Shape’, and the ‘Color-Shape Interaction’. To quantify the contribution of each component to the total signal (Fig. 3C), we calculated the Variance Accounted For (VAF) using the single-trial dataset. We projected the centered full-factorial data onto the demixing axes derived from the full dataset and computed the percentage of the total signal variance (sum of squared deviations) captured by each specific component. Importantly, this total variance explicitly encompasses all single-trial variability and condition-independent temporal dynamics inherent to the un-averaged ECoG signal.

To visualize the geometry of the representations (Fig. 3D, E), we projected the time-averaged neural states (i.e., the centroid of the population activity for each of the 9 conditions averaged over the 0–500 ms stimulus window) onto the dominant dPCA subspaces. To strictly quantify the clustering structure within these subspaces, we defined a distance-based Separation Index (SI) calculated on the pairwise Euclidean distances between the condition centroids. For a given feature (e.g., Color), the index was defined as:

$${Separation}\,{Index}=\frac{{\mu }_{{inter}}\,-\,{\mu }_{{intra}}}{{\mu }_{{inter}}+\,{\mu }_{{intra}}}$$

Where *µ*_*intra*_ is the mean distance between centroids of the same category (e.g., Red Bar vs. Red Cross) and *µ*_*inter*_ is the mean distance between centroids of different categories (e.g., Red Bar vs. Green Bar). This index ranges from -1 to 1, where positive values indicate that the neural geometry is organized according to the tested feature (i.e., conditions sharing the same feature are clustered together).

### Waveform-based dynamic manifold analyses

#### dPCA of oscillatory waveforms

To investigate the physical implementation of the neural code (i.e., Phase Code versus AM Code; Fig. 4), we applied dPCA directly to the band-pass filtered continuous signals. Raw ECoG signals were first band-pass filtered to isolate the gamma-band oscillation (see “Data preprocessing” for filter details). The filtered signal, preserving phase information, was z-scored based on global baseline statistics. Specifically, for each channel, we computed a single mean and standard deviation from the pooled pre-stimulus baseline periods of all trials and applied this normalization to the entire dataset. This “global z-score” preserves the relative amplitude differences across trials and conditions, which is critical for testing amplitude modulation hypotheses. Data were organized into a three-dimensional tensor (*N* channels × *C* conditions × *T* time points). We utilized standard dPCA to decompose the neural activity *X (c, t)* (where *c* is the condition, e.g., color, and *t* is time) into three non-overlapping additive components:

$$X(c,t)\approx {X}_{{Time}}(t)+{X}_{{Color}}(c)+{X}_{{Interaction}}(c,t)$$

The Time Component (*X*_*Time*_(*t*)) captures task-related dynamics shared across all conditions (e.g., common evoked potentials), acting as a condition-invariant carrier. The remaining variance tests two distinct physical coding hypotheses: (1) Static DC/Phase Shift (Hypothesis 1): Information is encoded via a time-invariant shift (e.g., *Signal* ≈ sin(*t*) + ∆*c*). This static variance is captured by the Color Component (*X*_*Color*_(*c*)). (2) Dynamic Amplitude Coding (Hypothesis 2): Information is encoded via multiplicative modulation of the carrier amplitude (e.g., *Signal* ≈ *A*(*c*) × sin(*t*)). Since this variance depends on both condition and time, it is captured by the Interaction Component (*X*_*Interaction*_(*c*, *t*)). The relative variance explained (VAF) by these components was calculated to quantify the contribution of each coding scheme.

#### Manifold trajectory visualization and stability analysis

To identify the low-dimensional geometric structure of the representation and rigorously test its stability (Fig. 4C), we implemented a strict cross-validation scheme. For each condition (e.g., color), trials were randomly partitioned into two non-overlapping, balanced subsets (Split 1 and Split 2). We then used dPCA in a two-step manner. First, dPCA was applied exclusively to the training data (Split 1) to learn a set of demixing axes that optimally isolated the variance related to the feature of interest (e.g., color). These axes, defining the feature-specific subspace, were then held fixed. In the second step, these fixed axes were used to project the trial-averaged data from both the original Split 1 and the entirely held-out Split 2, yielding two sets of low-dimensional neural trajectories within the same, consistently defined subspace.

To quantify the geometric similarity of these trajectories (Fig. 4D), we employed Procrustes analysis^62^. This method compares the intrinsic shape of two trajectories (treated as point clouds in N-dimensional space) while being insensitive to trivial rotational or reflectional differences. For each condition, the trajectory from Split 1 was optimally aligned to the corresponding trajectory from Split 2. The resulting Procrustes distance, *d*, was converted to a similarity score (1 - *d*). A high similarity score indicates that the geometric shape of the neural representation is highly stable and generalizable across independent sets of trials.

To test whether this topological conservation was maintained across different temporal dynamics (Supplementary Fig. 7), we applied the exact same Procrustes similarity framework to specific segmented temporal windows (the early transient phase: 0–200 ms, and the sustained phase: 300–500 ms). Furthermore, to evaluate the frequency specificity of the geometric stability (Supplementary Fig. 8), we applied this identical dPCA and cross-validated Procrustes analysis pipeline to the lower frequency bands defined in the “Frequency band definition” section. Additionally, the broad gamma band was further subdivided into low-gamma (40–55 Hz) and high-gamma (65–80 Hz) sub-bands for this specific analysis.

#### Time-resolved geometric separation analysis

To quantify the temporal evolution of the manifold geometry and measure the separability of representations over time (Fig. 5), we performed a time-resolved, cross-validated trajectory distance analysis. Unlike the static analysis which focused on the averaged stimulus window, this analysis utilized the full time-course of the neural activity. Consistent with the waveform dPCA, the band-pass filtered signals were z-scored based on global baseline statistics to preserve relative amplitude information. These normalized time-series were then projected onto the fixed feature-specific subspaces (i.e., the specific Interaction dPCs for Color or Shape) identified by the waveform dPCA. Crucially, to explicitly capture the dynamic amplitude modulations (AM) within these topological spaces, we extracted the amplitude envelope of these projected trajectories using the Hilbert transform. Separability was measured using the Mahalanobis distance, which normalizes the geometric distance by the intrinsic variability (“noise”) of the neural population. The robust noise covariance matrix was computed exclusively from the projected amplitude envelopes during the stimulus presentation period (0–500 ms). This ensures that the distance metric accurately reflects the intrinsic neural variability within the stimulus-driven state. We calculated two key quantities at each time point: (1) Within-condition distance: defined as the average Mahalanobis distance between centroids of the same condition across the two independent splits (e.g., Red-Split1 vs. Red-Split2). (2) Cross-condition distance: defined as the average distance between centroids of different conditions across the splits (e.g., Red-Split1 vs. Green-Split2). The direct point-by-point comparison between these two measures served to evaluate our representational separation.

### Statistics and Reproducibility

Experimental reproducibility was evaluated independently for the two subjects (*n* = 2 animals). No data were pooled across subjects. For within-subject statistical analyses, individual completed single trials (*n* = 1,836 for Monkey L; *n* = 2,106 for Monkey Q) or bootstrap/permutation iterations (*n* = 1,000) were defined and utilized as replicates.

#### Statistical analysis for feature selectivity (ANOVA)

To assess the significance of feature selectivity (Fig. 1D), we applied a False Discovery Rate (FDR) correction^63^ to the p-values obtained from the one-way ANOVAs performed at each of the 128 electrodes. The significance threshold was set at *p* < 0.05 (FDR corrected) to correct for multiple comparisons across channels.

#### Statistical analysis for decoding

The statistical significance of decoding performance was assessed as follows:

Searchlight Decoding (Figs. 2A, 2C): A null distribution of accuracy was generated at each electrode location via a permutation test (1,000 shuffles of labels). The resulting p-values were corrected for multiple comparisons across all electrode locations using the FDR procedure (*p* < 0.05, FDR corrected).

Cross-Temporal Decoding (Figs. 2B, 2D): A null distribution of accuracy was generated for each time-point pair in the temporal generalization matrix via a permutation test (1,000 shuffles of labels). The resulting p-values were corrected for multiple comparisons across all tested time points using the FDR procedure (*p* < 0.05, FDR corrected).

#### Statistical analysis for subspace separation index

To evaluate the statistical significance of the geometric clustering in the dPCA subspaces (Fig. 3E), the observed Separation Index was compared against a chance-level null distribution. This distribution was generated by randomly shuffling the stimulus condition labels (1,000 iterations) before calculating the pairwise distances and the resulting Separation Index. Significance was defined as an empirical Separation Index exceeding the 95th percentile of the null distribution (*p* < 0.05).

#### Statistical analysis for geometric separation

To determine the statistical significance of the representational separation over time, we employed a bootstrapping procedure across the time series. Specifically, we generated 1,000 bootstrap samples by randomly resampling trials with replacement within each condition. For each iteration, we computed the instantaneous Mahalanobis distance for both within- and cross-color pairs using the dPCA subspace established in the waveform-based dynamic manifold analysis. To estimate the 95% confidence intervals for these distance metrics, we employed the percentile bootstrap method by extracting the 2.5th and 97.5th percentiles from the 1,000 resampled iterations. Statistical significance of the distance difference (cross-color versus within-color) at each time point was initially assessed using uncorrected p-values derived from this bootstrap distribution. To correct for multiple comparisons across the continuous time series, we subsequently applied a non-parametric, cluster-based permutation test (1,000 permutations)^64^. Significant clusters were identified by comparing the observed cluster mass to a null distribution generated by randomly flipping the signs of the bootstrap differences, a procedure that preserves the temporal autocorrelation structure of the data (cluster-corrected *p* < 0.05).

#### Statistical analysis for procrustes geometric stability

To rigorously assess the statistical significance of the topological shape similarity (Fig. 4D and Supplementary Figs. 7 and 8), we compared the observed Procrustes similarity scores against a null distribution representing chance-level geometric alignment. This null distribution was generated using a time-scrambling surrogate approach. For each of the 1,000 permutation iterations, the time points of the high-dimensional neural trajectories in the test set (Split 2) were randomly shuffled before performing the Procrustes alignment with the training set (Split 1). This procedure preserved the overall variance and state-space volume of the data while destroying any deterministic, time-locked topological structure. The empirical similarity was considered significant if it exceeded the 99.9th percentile of this time-scrambled null distribution (*p* < 0.001).

### Reporting Summary

Further information on research design is available in the Nature Portfolio Reporting Summary linked to this article.

## Supplementary information

Transparent Peer Review file

Supplementary Information (Supplementary Figs. 1–8)

Description of Additional Supplementary Files

Supplementary Data 1

Nature Portfolio Reporting Summary

## Data Availability

The numerical source data underlying all graphs and charts in the main and Supplementary Figs. are provided as Supplementary Data 1. The neural datasets required to reproduce the main geometric and decoding analyses are deposited in the Zenodo repository^65,66^. The full raw dataset ( ~ 33 GB) is available from the corresponding author upon reasonable request.

## Code availability

All original custom code generated for data preprocessing, spectral analysis, and dynamic manifold geometry in this study has been deposited in the Zenodo repository^65,66^. These repositories contain the custom analysis pipelines (utilizing MATLAB, FieldTrip Toolbox, and LIBLINEAR) along with documentation specifying the exact software versions and processing parameters used to analyze the dataset.

## References

[1] Hubel, D. H. & Wiesel, T. N. Receptive fields, binocular interaction and functional architecture in the cat’s visual cortex. * J. Physiol.* **160**, 106–154 (1962). 10.1113/jphysiol.1962.sp006837 PMC1359523 14449617

[2] Vyas, S., Golub, M. D., Sussillo, D. & Shenoy, K. V. Computation through neural population dynamics. *Annu. Rev. Neurosci.* **43**, 249–275 (2020). 10.1146/annurev-neuro-092619-094115 PMC7402639 32640928

[3] Gallego, J. A., Perich, M. G., Miller, L. E. & Solla, S. A. Neural manifolds for the control of movement. *Neuron* **94**, 978–984 (2017). 10.1016/j.neuron.2017.05.025 PMC6122849 28595054

[4] Cunningham, J. P. & Yu, B. M. Dimensionality reduction for large-scale neural recordings. *Nat. Neurosci.* **17**, 1500–1509 (2014). 10.1038/nn.3776 PMC4433019 25151264

[5] Fries, P. Rhythms for cognition: communication through coherence. *Neuron* **88**, 220–235 (2015). 10.1016/j.neuron.2015.09.034 PMC4605134 26447583

[6] Singer, W. & Gray, C. M. Visual feature integration and the temporal correlation hypothesis. *Annu. Rev. Neurosci.* **18**, 555–586 (1995). 10.1146/annurev.ne.18.030195.003011 7605074

[7] Wang, X.-J. Neurophysiological and computational principles of cortical rhythms in cognition. *Physiol. Rev.* **90**, 1195–1268 (2010). 10.1152/physrev.00035.2008 PMC2923921 20664082

[8] Tanaka, K. Inferotemporal cortex and object vision. *Annu. Rev. Neurosci.* **19**, 109–139 (1996). 10.1146/annurev.ne.19.030196.000545 8833438

[9] DiCarlo, J. J., Zoccolan, D. & Rust, N. C. How does the brain solve visual object recognition? *Neuron* **73**, 415–434 (2012). 10.1016/j.neuron.2012.01.010 PMC3306444 22325196

[10] DiCarlo, J. J. & Cox, D. D. Untangling invariant object recognition. *Trends Cogn. Sci.* **11**, 333–341 (2007). 10.1016/j.tics.2007.06.010 17631409

[11] Kriegeskorte, N. & Wei, X.-X. Neural tuning and representational geometry. *Nat. Rev. Neurosci.* **22**, 703–718 (2021). 10.1038/s41583-021-00502-3 34522043

[12] De Baene, W. & Vogels, R. Effects of adaptation on the stimulus selectivity of macaque inferior temporal spiking activity and local field potentials. *Cereb. Cortex* **20**, 2145–2165 (2010). 10.1093/cercor/bhp277 20038542

[13] Buzsáki, G., Anastassiou, C. A. & Koch, C. The origin of extracellular fields and currents — EEG, ECoG, LFP and spikes. *Nat. Rev. Neurosci.* **13**, 407–420 (2012). 10.1038/nrn3241 PMC4907333 22595786

[14] Shinn, M. Phantom oscillations in principal component analysis. *Proc. Natl. Acad. Sci.* **120**, e2311420120 (2023). 10.1073/pnas.2311420120 PMC10691246 37988465

[15] Kuzmina, E., Kriukov, D. & Lebedev, M. Neuronal travelling waves explain rotational dynamics in experimental datasets and modelling. *Sci. Rep.* **14**, 3566 (2024). 10.1038/s41598-024-53907-2 PMC10861525 38347042

[16] van Bree, S., Levenstein, D., Krause, M. R., Voytek, B. & Gao, R. Processes and measurements: a framework for understanding neural oscillations in field potentials. *Trends Cogn. Sci.* **29**, 448–466 (2025). 10.1016/j.tics.2024.12.003 39753446

[17] Lachaux, J., Rodriguez, E., Martinerie, J. & Varela, F. J. Measuring phase synchrony in brain signals. *Hum. Brain Mapp.* **8**, 194–208 (1999). 10.1002/(SICI)1097-0193(1999)8:4<194::AID-HBM4>3.0.CO;2-C PMC6873296 10619414

[18] Engel, A. K., Fries, P. & Singer, W. Dynamic predictions: Oscillations and synchrony in top–down processing. *Nat. Rev. Neurosci.* **2**, 704–716 (2001). 10.1038/35094565 11584308

[19] Akam, T. & Kullmann, D. M. Oscillations and filtering networks support flexible routing of information. *Neuron* **67**, 308–320 (2010). 10.1016/j.neuron.2010.06.019 PMC3125699 20670837

[20] Wilson, H. R. & Cowan, J. D. Excitatory and inhibitory interactions in localized populations of model neurons. *Biophys. J.* **12**, 1–24 (1972). 10.1016/S0006-3495(72)86068-5 PMC1484078 4332108

[21] Churchland, M. M. et al. Neural population dynamics during reaching. *Nature* **487**, 51–56 (2012). 10.1038/nature11129 PMC3393826 22722855

[22] Gallego-Carracedo, C., Perich, M. G., Chowdhury, R. H., Miller, L. E. & Gallego, J. Á Local field potentials reflect cortical population dynamics in a region-specific and frequency-dependent manner. *eLife* **11**, e73155 (2022). 10.7554/eLife.73155 PMC9470163 35968845

[23] Moghaddam, A. S., Mooziri, M., Raoufy, M. R. & Mirshekar, M. A. Olfactory bulb-medial prefrontal cortex circuit slow oscillations encode working memory representations. *bioRxiv* 2025.10.30.685257 10.1101/2025.10.30.685257 (2025).

[24] Pollina, L. et al. Decoupling simultaneous motor imagination and execution via orthogonal ECoG neural representations. *Nat. Commun.* **17**, 4662 (2026). 10.1038/s41467-026-71234-0 PMC13201640 41917049

[25] Humphries, M. D. Strong and weak principles of neural dimension reduction. *arXiv* **5**, (2020).

[26] Tallon-Baudry, C. & Bertrand, O. Oscillatory gamma activity in humans and its role in object representation. *Trends Cogn. Sci.* **3**, 151–162 (1999). 10.1016/s1364-6613(99)01299-1 10322469

[27] von Stein, A. & Sarnthein, J. Different frequencies for different scales of cortical integration: from local gamma to long range alpha/theta synchronization. *Int J. Psychophysiol. Off. J. Int Organ. Psychophysiol.* **38**, 301–313 (2000). 10.1016/s0167-8760(00)00172-0 11102669

[28] Bastos, A. M. et al. Visual areas exert feedforward and feedback influences through distinct frequency channels. *Neuron* **85**, 390–401 (2015). 10.1016/j.neuron.2014.12.018 25556836

[29] Lamme, V. A. F. & Roelfsema, P. R. The distinct modes of vision offered by feedforward and recurrent processing. *Trends Neurosci.* **23**, 571–579 (2000). 10.1016/s0166-2236(00)01657-x 11074267

[30] Kohn, A. Visual adaptation: physiology, mechanisms, and functional benefits. *J. Neurophysiol.* **97**, 3155–3164 (2007). 10.1152/jn.00086.2007 17344377

[31] Müller, J. R., Metha, A. B., Krauskopf, J. & Lennie, P. Information conveyed by onset transients in responses of striate cortical neurons. *J. Neurosci.* **21**, 6978–6990 (2001). 10.1523/JNEUROSCI.21-17-06978.2001 PMC6763098 11517285

[32] Ray, S. & Maunsell, J. H. R. Differences in gamma frequencies across visual cortex restrict their possible use in computation. *Neuron* **67**, 885–896 (2010). 10.1016/j.neuron.2010.08.004 PMC3001273 20826318

[33] Jazayeri, M. & Afraz, A. Navigating the neural space in search of the neural code. *Neuron* **93**, 1003–1014 (2017). 10.1016/j.neuron.2017.02.019 28279349

[34] Chung, S. & Abbott, L. F. Neural population geometry: An approach for understanding biological and artificial neural networks. *Curr. Opin. Neurobiol.* **70**, 137–144 (2021). 10.1016/j.conb.2021.10.010 PMC10695674 34801787

[35] Kriegeskorte, N. & Kievit, R. A. Representational geometry: integrating cognition, computation, and the brain. *Trends Cogn. Sci.* **17**, 401–412 (2013). 10.1016/j.tics.2013.06.007 PMC3730178 23876494

[36] Carandini, M. & Heeger, D. J. Normalization as a canonical neural computation. *Nat. Rev. Neurosci.* **13**, 51–62 (2012). 10.1038/nrn3136 PMC3273486 22108672

[37] Montijn, J. S., Klink, P. C. & van Wezel, R. J. A. Divisive normalization and neuronal oscillations in a single hierarchical framework of selective visual attention. *Front Neural Circuit* **6**, 22 (2012). 10.3389/fncir.2012.00022 PMC3343306 22586372

[38] Mullen, K. T. & Beaudot, W. H. A. Comparison of color and luminance vision on a global shape discrimination task. *Vis. Res.* **42**, 565–575 (2002). 10.1016/s0042-6989(01)00305-4 11853774

[39] Beaudot, W. H. A. & Mullen, K. T. Orientation selectivity in luminance and color vision assessed using 2-d band-pass filtered spatial noise. *Vis. Res.* **45**, 687–696 (2005). 10.1016/j.visres.2004.09.023 15639495

[40] Conway, B. R. & Tsao, D. Y. Color-tuned neurons are spatially clustered according to color preference within alert macaque posterior inferior temporal cortex. *Proc. Natl. Acad. Sci.* **106**, 18034–18039 (2009). 10.1073/pnas.0810943106 PMC2764907 19805195

[41] Lafer-Sousa, R. & Conway, B. R. Parallel, multi-stage processing of colors, faces and shapes in macaque inferior temporal cortex. *Nat. Neurosci.* **16**, 1870–1878 (2013). 10.1038/nn.3555 PMC3957328 24141314

[42] Fujita, I., Tanaka, K., Ito, M. & Cheng, K. Columns for visual features of objects in monkey inferotemporal cortex. *Nature* **360**, 343–346 (1992). 10.1038/360343a0 1448150

[43] Dubey, A. & Ray, S. Cortical electrocorticogram (ECoG) is a local signal. *J. Neurosci.* **39**, 4299–4311 (2019). 10.1523/JNEUROSCI.2917-18.2019 PMC6538865 30914446

[44] Fiebelkorn, I. C., Pinsk, M. A. & Kastner, S. A dynamic interplay within the frontoparietal network underlies rhythmic spatial attention. *Neuron* **99**, 842–853.e8 (2018). 10.1016/j.neuron.2018.07.038 PMC6474777 30138590

[45] Fiebelkorn, I. C. & Kastner, S. A rhythmic theory of attention. *Trends Cogn. Sci.* **23**, 87–101 (2019). 10.1016/j.tics.2018.11.009 PMC6343831 30591373

[46] Afraz, S.-R., Kiani, R. & Esteky, H. Microstimulation of inferotemporal cortex influences face categorization. *Nature* **442**, 692–695 (2006). 10.1038/nature04982 16878143

[47] Kar, K., Duijnhouwer, J. & Krekelberg, B. Transcranial alternating current stimulation attenuates neuronal adaptation. *J. Neurosci.* **37**, 2325–2335 (2017). 10.1523/JNEUROSCI.2266-16.2016 PMC5354346 28137971

[48] Mazor, O. & Laurent, G. Transient dynamics versus fixed points in odor representations by locust antennal lobe projection neurons. *Neuron* **48**, 661–673 (2005). 10.1016/j.neuron.2005.09.032 16301181

[49] Rabinovich, M. I., Huerta, R., Varona, P. & Afraimovich, V. S. Transient cognitive dynamics, metastability, and decision making. *PLoS Comput. Biol.* **4**, e1000072 (2008). 10.1371/journal.pcbi.1000072 PMC2358972 18452000

[50] Sadtler, P. T. et al. Neural constraints on learning. *Nature* **512**, 423–426 (2014). 10.1038/nature13665 PMC4393644 25164754

[51] Majaj, N. J., Hong, H., Solomon, E. A. & DiCarlo, J. J. Simple learned weighted sums of inferior temporal neuronal firing rates accurately predict human core object recognition performance. *J. Neurosci.* **35**, 13402–13418 (2015). 10.1523/JNEUROSCI.5181-14.2015 PMC4588611 26424887

[52] Hung, C. P., Kreiman, G., Poggio, T. & DiCarlo, J. J. Fast readout of object identity from macaque inferior temporal cortex. *Science* **310**, 863–866 (2005). 10.1126/science.1117593 16272124

[53] Tanigawa, H. et al. Decoding distributed oscillatory signals driven by memory and perception in the prefrontal cortex. *Cell Rep.* **39**, 110676 (2022). 10.1016/j.celrep.2022.110676 35417680

[54] Zhou, T. et al. Mapping information flow between the inferotemporal and prefrontal cortices via neural oscillations in memory retrieval and maintenance. *Cell Rep.* **42**, 113169 (2023). 10.1016/j.celrep.2023.113169 37740917

[55] Matsuo, T. et al. Intrasulcal electrocorticography in macaque monkeys with minimally invasive neurosurgical protocols. *Front. Syst. Neurosci.* **5**, 34 (2011). 10.3389/fnsys.2011.00034 PMC3103840 21647392

[56] Oostenveld, R., Fries, P., Maris, E. & Schoffelen, J.-M. FieldTrip: open source software for advanced analysis of MEG, EEG, and invasive electrophysiological data. *Comput. Intel. Neurosc*. **2011**, 156869 (2011). 10.1155/2011/156869 PMC3021840 21253357

[57] Li, C., Hasegawa, I. & Tanigawa, H. Protocol for assisting frequency band definition and decoding neural dynamics using hierarchical clustering and multivariate pattern analysis. *STAR Protoc.* **6**, 103870 (2025). 10.1016/j.xpro.2025.103870 PMC12171811 40465456

[58] Kriegeskorte, N., Goebel, R. & Bandettini, P. Information-based functional brain mapping. *Proc. Natl. Acad. Sci. USA*. **103**, 3863–3868 (2006). 10.1073/pnas.0600244103 PMC1383651 16537458

[59] Fan, R.-E., Chang, K.-W., Hsieh, C.-J., Wang, X.-R. & Lin, C.-J. LIBLINEAR: A library for large linear classification. *J. Mach. Learn. Res.* **9**, 1871–1874 (2008).

[60] King, J.-R. & Dehaene, S. Characterizing the dynamics of mental representations: the temporal generalization method. *Trends Cogn. Sci.* **18**, 203–210 (2014). 10.1016/j.tics.2014.01.002 PMC5635958 24593982

[61] Kobak, D. et al. Demixed principal component analysis of neural population data. *eLife* **5**, e10989 (2016). 10.7554/eLife.10989 PMC4887222 27067378

[62] Gower, J. C. Generalized procrustes analysis. *Psychometrika* **40**, 33–51 (1975).

[63] Benjamini, Y. & Hochberg, Y. Controlling the false discovery rate: a practical and powerful approach to multiple testing. *J. R. Stat. Soc. Ser. B Methodol.* **57**, 289–300 (1995).

[64] Maris, E. & Oostenveld, R. Nonparametric statistical testing of EEG- and MEG-data. *J. Neurosci. Meth* **164**, 177–190 (2007). 10.1016/j.jneumeth.2007.03.024 17517438

[65] Li, C., Hasegawa, I. & Tanigawa, H. Code&Data for: Gamma oscillations provide a stable geometric scaffold for color representation in primate inferior temporal cortex [Data set]. *Zenodo*10.5281/zenodo.20566247 (2026). PMC13737192 42436348

[66] Li, C., Hasegawa, I. & Tanigawa, H. Data and Codes from: Protocol for assisting frequency band definition and decoding neural dynamics using hierarchical clustering and multivariate pattern analysis [Data set]. *Zenodo*10.5281/zenodo.15203774 (2026). PMC12171811 40465456

