# Supplementary material for: Gamma oscillations provide a stable geometric scaffold for color representation in primate inferior temporal cortex
Source: Commun Biol. 2026 Jul 11;9:1455. doi: 10.1038/s42003-026-10652-8 (PMC13737192; doi:10.1038/s42003-026-10652-8)
Supplement: Supplementary file 1 [file 42003_2026_10652_MOESM1_ESM.pdf]

nature portfolio

Peer Review File

Gamma oscillations provide a stable geometric scaffold for color representation in primate inferior temporal cortex

Corresponding Author: Dr Hisashi Tanigawa

This file contains all reviewer reports in order by version, followed by all author rebuttals in order by version.

Version 0:

Reviewer comments:

Reviewer #1

(Remarks to the Author)
The question posed is how does the brain maintain a stable representation in the face of dynamic neural activity. The authors conclude that a dynamic trajectory of gamma-band amplitude across the inferotemporal cortex (independent of gamma phase) stably encodes the color of a centrally presented visual stimulus. The analysis seems reasonable and rigorous. For the most part, the conclusions are justified by the data, although in some cases the authors argue for an embrace of the null hypothesis that is probably not justified (such as that shape is not stably encoded in the same way as color, or that color is not encoded by mechanisms involving baseline shifts), but these are minor points that are not strongly emphasized by the authors and can probably just be rephased with no harm to the main conclusions. The two main limitations to my mind are (1) the passive nature of the task and (2) comparison of two different types of feature dimensions. The limitation of the passive nature of the task is that it somewhat undermines the “stable representation” assumption. For example, what if the monkey did not stably perceive the color (or shape) of the stimulus across all phases of a gamma cycle, but rather rapidly alternated between “good” and “bad” phases of perception? Without some behavioral read-out we can’t be sure. This type of rhythmic computation has recently become influential for slower frequency bands associated with attention and working memory (alpha and theta bands in particular, with the work of Ian Fiebelkorn being particularly influential in this area) and one could imagine a similar rhythmic process might also characterize low-level sensory perceptual processes that have been linked to gamma-band responses. In other words, although the authors present a compelling case that we can stably decode the color from the gamma band activity over time, we can’t be sure that the *monkey* was also doing so. The limitation of comparing two different feature dimensions is that it is difficult to tell whether differences in e.g. decoding shape versus decoding color from neural activity are due to differences in neural representation thereof or rather differences in intrinsic “confusability” of the features (more on this below). However, these are somewhat “side issues” to the main conclusions of the authors, and as such I don’t think they’re very critical but should probably just be discussed in greater depth.

Figure 1d was a little disorienting. I wasn’t sure what the different points in the map represented, but supposed that they might be different ECoG electrodes. The “d” and “a” axes labels help somewhat, but without another illustration showing where the arrays were located in the context of the whole brain, the spatial maps are hard to interpret. Figure 2a helps a bit because it shows sulci as landmarks, but even there I think it would be helpful to have a figure that shows where the ECoG arrays are located relative to the whole lateral surface of the cortex in order to better understand which sulci are where (including labels on the sulci would also help).

Also in Figure 1d there are plot points that are blue for Monkey Q, but the color scale doesn’t include any blue, so these are hard to interpret. The blue seems to be between the electrode points; are these blue values artifactual due perhaps to spline interpolation? It would also be helpful to include more values on the color scale besides just the extremes (0 and 20 are the only values shown currently) in order to better interpret intermediate values.

One challenge in comparing the neural decoding accuracy for color to that for shape is that it's hard to know if the different categories being decoded are equivalently distinct. For example, if the three shapes were just three rectangles with slightly different aspect ratios while the three colors were red, green and blue, it shouldn’t be surprising that neural decoding for color performs better. Conversely, if the three colors were three slightly different shades of red, while the three shapes were a star, a circle, and a dog, then perhaps the decoding performance for shape might outperform color. That is, is the difference in decoding performance an intrinsic property of the brain area being studied, or a reflection of the relative difficulty of the two

decoding “tasks”? It’s a tricky problem to solve because ultimately color and shape aren’t directly comparable. Picking categories with similar psychophysical discriminability could be one way of approaching this, although that’s certainly no magic bullet either and the current study doesn’t seem to have those data anyway. Perhaps there’s a way to address this concern with the literature or some other type of “grounding” within the current dataset. At the very least the limitation should be discussed. It seems like the section around line 370 would be a natural place to mention this. The main conclusions of the report are rightly about the representational geometry of color itself, and strong conclusions aren’t made by the authors about the comparison between the different feature dimensions, so this is only a minor point. The comparison of the stability of representational geometry for the two feature dimensions within animal Q where decoding performance was more “matched” was a compelling inclusion (line 272).

The remark about gamma oscillations being zero-mean and thus baseline shifts can’t be carrying the code (around line 308) may be a bit off the mark. Because we’re just filtering around the gamma band we’re not sensitive to other physiological phenomena that would manifest as baseline shifts. That is, some signals are not observed but we should not conclude those aren’t contributing. There’s enough of a story to tell in these data about the contributions of the signals that *were* observed, and the discussion would be improved to stay focused on those.

Figure 1b: can the authors indicate somehow which specific channels in the ECoG array are the “representative” channels shown?

The values for the distance metrics in figure 1e&f are hard to interpret. The color scales in figure 1e and the vertical axes in figure 1f are not labeled with units. From the methods I think the units would be something like mV*Hz (measured in a 127-dimensional space), which are also hard to interpret. Some normalization would be an improvement (such as proportion of the average distance of some null distribution or similar).

Around line 72, it’s not clear at that point what “scale-invariant” means. Spatial scale? Temporal scale? Scale in some high-dimensional state space? Etc. Could be revised to be clearer.

Around line 74, I was a little confused about whether “shape” referred to stimulus shape or shape of a neural manifold. Could be revised to be clearer.

Reviewer #2

(Remarks to the Author)
The authors record (128 channel ECoG) from the surface of the inferior temporal cortex of macaques and analyse the activity evoked by nine stimuli (3 shapes x 3 colours). Importantly, the colours were isoluminant to each other and also seemed to have been approximately isoluminant to the grey background. This is a bit unfortunate, because shapes defined by isoluminant colour contrast are not perceived and processed particularly well (e.g., Mullen & Beaudot, 2002, Vis Res; Beaudot & Mullen, 2005, Vis Res).

The results are analysed in several sophisticated and interesting ways. At the simplest level, trial-averaged gamma-power of many channels is found to distinguish stimulus colour and, to a much lesser extent, stimulus shape (Fig 1).

This finding is corroborated by a searchlight analysis -- which decodes colour/shape from 9 neighbouring channels -- and by a temporal generalisation analysis in which 128-channel decoders were trained on one 50 ms window and tested on another. The results consistently show far better representation of colour than of shape (Fig 2).

The more innovative analyses are based on demixed PCA, which divides (demixes) the high-dimensional signal (channels x time points x shapes x colours) such as to identify subspaces that are informative about 'colour', 'shape', or their 'interaction'. When applied to trial-averaged signals, this analysis confirms that more response variance distinguishes colour than shape (Fig. 3).

When applied to the time-varying signal, this analysis distinguishes between colour-dependent variance that is additive (i.e., time-independent) and that is multiplicative (i.e., colour- and time-dependent). The key finding is that multiplicative modulation explained far more variance (50%) than additive modulation (<1%). In the space of demixed PCs, particular stimulus colours elicited particular trajectories of activity (Fig. 4). The stability of these spatiotemporal trajectories was confirmed by cross-validated Procrustes similarity. In short, particular colours elicit stable and recognisable patterns of activity.

The separation (Mahalonobis distance) between trajectories increases and peaks shortly after stimulus presentation (i.e., during the first 200 ms), before returning to baseline (Fig. 5).

In contrast, stimulus shape is not encoded by stable trajectories (Fig. 6).

From my point of view, the really attractive aspects of this work are the colour-specific spatio-temporal trajectories across the inferotemporal cortex and the advanced computational methods used to extract and visualise them. Other recent work on the spatiotemporal structure of information-encoding responses in the inferior temporal cortex has not reached a comparable depth of analysis (e.g., Kakaei, Braun, 2024, Imaging Neurosci; Nigam, Schwiedrzik, 2024, Nat Comm; Nigam, Schwiedrzik, 2025, bioRxiv).

Accordingly, I would encourage the authors to focus their presentation on this aspect, rather than on the weak encoding of shape (which is owed to the stimulus set). Perhaps the authors might consider providing some more didactic visualisations of demixing PCA? I would also be fascinated to see a larger version of Fig. 4b, with trajectories depicted not as curves but as three-dimensional sausages, such as to indicate the variability from trial to trial.

This work reaches an important conclusion -- consistent spatiotemporal responses -- with highly convincing methods and deserves wide recognition. The computational methods and results could be made more accessible to general readers.

Reviewer #3

(Remarks to the Author)
1. Brief summary of the manuscript.
This is a primate experiment carried out in higher-order inferior temporal visual areas where the authors apply a geometric data analysis to determine whether features of an oscillatory code provide a scaffold for visual information processing. The authors used amplitude modulations or raw voltage as oscillatory features, and they decoded shape and color information during a passive visual task. The authors find a positive answer during the stimulus-induced periods, where gamma amplitude modulations are robustly measurable for color processing but not for shape.

2. Overall impression of the work.
I find this paper compelling, timely, and highly relevant. The authors extend the manifold approach to oscillations, which is far less common relative to spiking data. In an effort toward transparency, the authors show the results of each monkey separately throughout the paper, which I applaud. The entire data analysis is very rigorous, combining basic signal processing with advanced geometric spatio-temporal analysis.

3. Specific comments, with recommendations for addressing each comment.
- Classic and recent literature regarding low dimensionality and neuronal oscillations.
Regarding the oscillatory manifold, previous attempts by other authors should be acknowledged. Here I list a few such examples within the recent literature, such as Churchland et al. (2012), Gallego-Carracedo et al. (2022), Moghaddam et al. (2025), and Polina et al. (2026). The authors should link their results with foundational work in the field of neuronal oscillations, such as the Wilson-Cowan (WC) 1972 seminal paper. The WC model links high-dimensional populations of excitatory and inhibitory neurons that can be represented in a 2-dimensional phase space. Although the authors use amplitude modulations to avoid the negative part, I think this seminal paper should be discussed because it indeed undertook such an endeavor in shaping the field.

- Role of dPCA and phantom oscillations.
There is recent work showing that oscillations can emerge after PCA decompositions, due to the orthogonality constraints implicit in dimensionality reduction and spectral analysis (sines, cosines, harmonics; see Shinn, 2023). Indeed, amplitude modulations can show peaks in the power spectrum (Nowak et al., 1992; Penttonen et al., 2003), and such amplitude modulations are, many times, coupled to the phase of slower oscillations (Tort et al., 2010; Canolty et al., 2016). It has been shown that jPCA (Churchland et al., 2012) can indeed confound traveling waves with low-dimensional rotational dynamics (Kuzmina et al., 2024). Can the authors determine whether dPCA suffers from similar limitations given the constraints described in Shinn (2023)? Note that gamma amplitude modulations could be periodic or not, and a power spectrum density of the gamma amplitude modulations would be very informative. Both positive and negative findings would be valuable for the community, given ongoing debates about whether neuronal oscillations are epiphenomenal (van Bree et al., 2025).

- Low vs. high dimensionality reduction.
Figure 4B indicates that 13 or 20 dPCs are necessary to account for 85% of variance, but the dPCA analysis finds mainly 2 (Figure 3C). How many principal components are needed to find a low-dimensional manifold? I am not aware of an absolute threshold, and the only paper that discusses a similar issue is Humphries (2021). I encourage the authors to discuss this and to situate their work within the weak vs. strong principles of dimensionality reduction (Humphries, 2021), unless the authors find a better solution.

References.
Canolty, R. T., & Knight, R. T. (2010). The functional role of cross-frequency coupling. Trends in cognitive sciences, 14(11), 506-515.
Churchland, M. M., Cunningham, J. P., Kaufman, M. T., Foster, J. D., Nuyujukian, P., Ryu, S. I., & Shenoy, K. V. (2012). Neural population dynamics during reaching. Nature, 487(7405), 51-56.
Freeman, W. J. (1992). Tutorial on neurobiology: from single neurons to brain chaos. International journal of bifurcation and chaos, 2(03), 451-482.
Gallego-Carracedo, C., Perich, M. G., Chowdhury, R. H., Miller, L. E., & Gallego, J. Á. (2022). Local field potentials reflect cortical population dynamics in a region-specific and frequency-dependent manner. Elife, 11, e73155.
Humphries, M. D. (2021). Strong and weak principles of neural dimension reduction. Neurons, Behavior, Data Analysis, and Theory, 5(2). doi:10.51628/001c.24619
Kuzmina, E., Kriukov, D., & Lebedev, M. (2024). Neuronal travelling waves explain rotational dynamics in experimental datasets and modelling. Scientific Reports, 14(1), 3566.
Moghaddam, A. S., Mooziri, M., Raoufy, M. R., & Mirshekar, M. A. (2025). Olfactory bulb-medial prefrontal cortex circuit slow oscillations encode working memory representations. bioRxiv, 2025-10.
Novak, P., Lepicovska, V., & Dostalek, C. (1992). Periodic amplitude modulation of EEG. Neuroscience letters, 136(2), 213-

215.
Penttonen, M., & Buzsáki, G. (2003). Natural logarithmic relationship between brain oscillators. Thalamus & Related Systems, 2(2), 145-152.
Pollina, L., Struber, L., de Seta, V., Russo, E., Karakas, S., Chabardes, S., ... & Micera, S. (2026). Decoupling simultaneous motor imagination and execution via orthogonal ECoG neural representations. Nature Communications.
Shinn, M. (2023). Phantom oscillations in principal component analysis. Proceedings of the National Academy of Sciences, 120(48), e2311420120.
Tort, A. B., Komorowski, R., Eichenbaum, H., & Kopell, N. (2010). Measuring phase-amplitude coupling between neuronal oscillations of different frequencies. Journal of neurophysiology, 104(2), 1195-1210.
van Bree, S., Levenstein, D., Krause, M. R., Voytek, B., & Gao, R. (2025). Processes and measurements: a framework for understanding neural oscillations in field potentials. Trends in cognitive sciences, 29(5), 448-466.
Wilson, H. R., & Cowan, J. D. (1972). Excitatory and inhibitory interactions in localized populations of model neurons. Biophysical journal, 12(1), 1-24.

Version 1:

Reviewer comments:

Reviewer #1

(Remarks to the Author)
My concerns have been addressed. Minor improvement to the revision: the font size for the value labels overlaid on the 3x3 similarity matrices in the figures could be made larger for readability

Reviewer #2

(Remarks to the Author)
The authors have adequately addressed all of my comments. They have added an explanatory figure panel (Fig. 4A) to illustrate their demixing PCA framework. The discussion now emphasizes oscillatory encoding of color information, rather than the weak encoding of shape information. They relate their findings to neural population dynamics and discuss the spatial resolution of their data. I do not see a need for further revisions.

Reviewer #3

(Remarks to the Author)
I thank the authors for their thorough and detailed point-by-point replies. They addressed all of the concerns raised. Congratulations on the great work.

Open Access This Peer Review File is licensed under a Creative Commons Attribution 4.0 International License, which permits use, sharing, adaptation, distribution and reproduction in any medium or format, as long as you give appropriate

credit to the original author(s) and the source, provide a link to the Creative Commons license, and indicate if changes were made.
In cases where reviewers are anonymous, credit should be given to 'Anonymous Referee' and the source.
The images or other third party material in this Peer Review File are included in the article's Creative Commons license, unless indicated otherwise in a credit line to the material. If material is not included in the article's Creative Commons license and your intended use is not permitted by statutory regulation or exceeds the permitted use, you will need to obtain permission directly from the copyright holder.
To view a copy of this license, visit https://creativecommons.org/licenses/by/4.0/

Reviewer #1 (Remarks to the Author):

The question posed is how does the brain maintain a stable representation in the face of dynamic neural activity. The authors conclude that a dynamic trajectory of gamma-band amplitude across the inferotemporal cortex (independent of gamma phase) stably encodes the color of a centrally presented visual stimulus. The analysis seems reasonable and rigorous. For the most part, the conclusions are justified by the data, although in some cases the authors argue for an embrace of the null hypothesis that is probably not justified (such as that shape is not stably encoded in the same way as color, or that color is not encoded by mechanisms involving baseline shifts), but these are minor points that are not strongly emphasized by the authors and can probably just be rephased with no harm to the main conclusions. The two main limitations to my mind are (1) the passive nature of the task and (2) comparison of two different types of feature dimensions.

[1-1]

The limitation of the passive nature of the task is that it somewhat undermines the “stable representation” assumption. For example, what if the monkey did not stably perceive the color (or shape) of the stimulus across all phases of a gamma cycle, but rather rapidly alternated between “good” and “bad” phases of perception? Without some behavioral read-out we can’t be sure. This type of rhythmic computation has recently become influential for slower frequency bands associated with attention and working memory (alpha and theta bands in particular, with the work of Ian Fiebelkorn being particularly influential in this area) and one could imagine a similar rhythmic process might also characterize low-level sensory perceptual processes that have been linked to gamma-band responses. In other words, although the authors present a compelling case that we can stably decode the color from the gamma band activity over time, we can’t be sure that the *monkey* was also doing so.

Reply:

We thank the reviewer for this highly insightful theoretical point. We completely agree. Because the present study employed a passive fixation task without behavioral read-outs, we cannot definitively claim that the monkey continuously or stably perceived the stimulus across all phases of the gamma cycle.

Our primary conclusion is strictly restricted to the neural representation level: the gamma-band activity in the ITC establishes a geometric representational scaffold (the oscillatory manifold) from which downstream areas could reliably decode information. However, as the reviewer appropriately points out, subjective perception is likely subjected to rhythmic modulation. Based on the prominent framework proposed by Fiebelkorn and Kastner, sensory representations may be rhythmically sampled, dynamically alternating between "sampling" and "shifting" phases coordinated by slower frequency bands such as theta or alpha.

Therefore, we agree that the stable geometric representations we observed in the gamma band are likely sampled intermittently by downstream cognitive networks rather than being perceived as a continuous stream. To clarify our claims, prevent overinterpretation of "representational stability," and incorporate this theoretical perspective, we have substantially expanded the limitations section in the Discussion. In doing so, we explicitly separated the topological persistence of the neural

representation from the continuity of subjective perception, citing the relevant works by Fiebelkorn and colleagues.

Changes to the manuscript:

We have revised the Discussion section (Pages 10–11, Lines 414–423) to read as follows:

Original text:

“Second, our reliance on a passive fixation task precludes direct observation of cognitive modulation. Active discrimination paradigms will be necessary to explicitly test our hypothesis that these dynamic structures function as an attention-dependent workspace.”

Revised text:

"Second, our reliance on a passive fixation task inherently limits our ability to equate the stability of the neural representation with the continuity of subjective perception. Recent theoretical and empirical frameworks propose that attention and sensory processing are not continuous, but operate via rhythmic sampling—dynamically alternating between "sampling" and "shifting" phases coordinated by slower frequency rhythms such as the theta and alpha bands[44,45]. Therefore, while the oscillatory manifold maintains a topologically rigid structure despite the time-varying nature of its trajectories, it is highly plausible that these representations are subjected to intermittent, rhythmic sampling by downstream cortical networks. Active discrimination paradigms will be necessary to explicitly test how these high-frequency geometric scaffolds are dynamically accessed by top-down attentional rhythms. "

44. Fiebelkorn, I. C., Pinsk, M. A. & Kastner, S. A dynamic interplay within the frontoparietal network underlies rhythmic spatial attention. Neuron 99, 842-853.e8 (2018).

45. Fiebelkorn, I. C. & Kastner, S. A rhythmic theory of attention. Trends Cogn. Sci. 23, 87–101 (2019).

[1-2]

The limitation of comparing two different feature dimensions is that it is difficult to tell whether differences in e.g. decoding shape versus decoding color from neural activity are due to differences in neural representation thereof or rather differences in intrinsic “confusability” of the features (more on this below). However, these are somewhat “side issues” to the main conclusions of the authors, and as such I don’t think they’re very critical but should probably just be discussed in greater depth.

Reply:

We completely agree with this insightful point. As the reviewer notes and further elaborates on this limitation in comment [1-5] below, we have consolidated our detailed response and the corresponding manuscript revisions there. Please refer to our combined response to comment [1-5].

[1-3]

Figure 1d was a little disorienting. I wasn't sure what the different points in the map represented, but supposed that they might be different ECoG electrodes. The "d" and "a" axes labels help somewhat, but without another illustration showing where the arrays were located in the context of the whole brain, the spatial maps are hard to interpret. Figure 2a helps a bit because it shows sulci as landmarks, but even there I think it would be helpful to have a figure that shows where the ECoG arrays are located relative to the whole lateral surface of the cortex in order to better understand which sulci are where (including labels on the sulci would also help).

Reply:

We agree that providing macro-scale cortical context and explicit anatomical landmarks clarifies the functional spatial maps. To address this, we have updated Figure 1D and Figure 2A by superimposing explicit sulcal landmarks (lines). Furthermore, we have updated Supplementary Figure 1A, which illustrates the structural localization of the ECoG arrays relative to the whole lateral and ventral surfaces of the cortex, to include identical sulcal labels for direct cross-reference.

Changes to the manuscript:

We have updated Figure 1D, Figure 2A, and Supplementary Figure 1A to include explicit sulcal lines and labels (STS, AMTS, and RS). The corresponding figure legends in the Main Text and Supplementary Information have been revised to reflect these visual updates.

The Figure 1 legend (Page 26, Lines 950–951):

"(D) … Black dots indicate individual ECoG electrodes. Major sulci are overlaid: STS (superior temporal sulcus), AMTS (anterior middle temporal sulcus), and RS (rhinal sulcus)."

The Figure 2 legend (Page 27, Line 966):

"(A) … Major sulci are overlaid as black lines: STS, AMTS, and RS. …"

The Supplementary Figure 1 legend:

"(A) … Major sulci are labeled: STS (superior temporal sulcus), AMTS (anterior middle temporal sulcus), and RS (rhinal sulcus). …"

[1-4]

Also in Figure 1d there are plot points that are blue for Monkey Q, but the color scale doesn't include any blue, so these are hard to interpret. The blue seems to be between the electrode points; are these blue values artifactual due perhaps to spline interpolation? It would also be helpful to include more values on the color scale besides just the extremes (0 and 20 are the only values shown currently) in order to better interpret intermediate values.

Reply:

We have corrected the color axis to [0, 10] and incorporated intermediate tick marks into the colorbars to clarify the intermediate values, as requested.

Changes to the manuscript:

Figure 1D has been updated to reflect the accurate [0, 10] scale with explicit intermediate tick marks.

[1-5]

One challenge in comparing the neural decoding accuracy for color to that for shape is that it's hard to know if the different categories being decoded are equivalently distinct. For example, if the three shapes were just three rectangles with slightly different aspect ratios while the three colors were red, green and blue, it shouldn't be surprising that neural decoding for color performs better. Conversely, if the three colors were three slightly different shades of red, while the three shapes were a star, a circle, and a dog, then perhaps the decoding performance for shape might outperform color. That is, is the difference in decoding performance an intrinsic property of the brain area being studied, or a reflection of the relative difficulty of the two decoding "tasks"? It's a tricky problem to solve because ultimately color and shape aren't directly comparable. Picking categories with similar psychophysical discriminability could be one way of approaching this, although that's certainly no magic bullet either and the current study doesn't seem to have those data anyway. Perhaps there's a way to address this concern with the literature or some other type of "grounding" within the current dataset. At the very least the limitation should be discussed. It seems like the section around line 370 would be a natural place to mention this. The main conclusions of the report are rightly about the representational geometry of color itself, and strong conclusions aren't made by the authors about the comparison between the different feature dimensions, so this is only a minor point. The comparison of the stability of representational geometry for the two feature dimensions within animal Q where decoding performance was more "matched" was a compelling inclusion (line 272).

Reply:

We completely agree that the intrinsic psychophysical "confusability" or discriminability of the color and shape categories was not explicitly matched in our passive viewing paradigm. Consequently, as the reviewer correctly notes, comparing the absolute decoding performance between these two feature dimensions is inherently limited.

However, as the reviewer also insightfully acknowledged, our primary conclusion regarding the dissociation between color and shape relies on the representational geometry, rather than absolute decoding accuracy. The critical finding is the structural contrast observed in Monkey Q: even when shape information was highly decodable from the gamma-band power envelope (indicating sufficient signal availability), it failed to form a topologically stable trajectory in the oscillatory waveform domain. This dissociation demonstrates that the emergence of the oscillatory manifold requires a specialized geometric architecture, rather than merely reflecting differences in signal-to-noise ratio or feature discriminability.

To explicitly address this constraint and prevent any overinterpretation, we have added a dedicated paragraph to the Limitations section in the Discussion, clarifying that color and shape cannot be directly compared regarding absolute neural decodability due to the lack of psychophysical matching.

Changes to the manuscript:

We have added the following text to the Limitations section in the Discussion (Page 11, Lines 423–431):

"Third, although our factorial design isolated visual features, the intrinsic psychophysical discriminability of color and shape categories was not equated. Lacking behavioral read-outs of perceptual thresholds, we cannot determine whether lower shape decodability stems from fundamental neural representation differences or the relative difficulty of implicit discrimination. However, our use of the shape condition as a methodological control rests on representational geometry rather than absolute decoding accuracy. As observed in our data (Fig. 2D and Fig. 6), even when shape information is effectively decodable from static gamma power, it lacks the topologically stable trajectory inherent to the color manifold. This confirms that the oscillatory manifold is a specialized geometric format, not merely a byproduct of high signal-to-noise ratio."

[1-6]

The remark about gamma oscillations being zero-mean and thus baseline shifts can't be carrying the code (around line 308) may be a bit off the mark. Because we're just filtering around the gamma band we're not sensitive to other physiological phenomena that would manifest as baseline shifts. That is, some signals are not observed but we should not conclude those aren't contributing. There's enough of a story to tell in these data about the contributions of the signals that *were* observed, and the discussion would be improved to stay focused on those.

Reply:

We fully agree that our previous statement was an overreach. Because our analysis relies on band-pass filtering, which inherently centers the signal and removes low-frequency fluctuations, we cannot rule out the potential contributions of other physiological phenomena that might manifest as baseline shifts. Following the reviewer's constructive advice, we have removed this speculative remark. This deletion improves the Discussion by keeping it strictly focused on the observed dynamics—the amplitude modulation of gamma oscillations—without making unsupported claims about unobserved signals.

Changes to the manuscript:

We have revised the Discussion (Page 8, Lines 321–328) by deleting the speculative sentence regarding baseline shifts and zero-mean oscillations. The revised paragraph now maintains a strict focus on the observed amplitude modulation code, reading as follows:

Original text:

"This waveform-based analysis provides mechanistic insight into how sensory information is structurally encoded within high-frequency networks. Given the zero-mean nature of gamma oscillations, neural trajectories cannot rely on static baseline shifts to segregate distinct representations. Instead, our findings (Fig. 4) reveal that the cortex implements this code via a dynamic interaction between a condition-independent carrier oscillation and feature-specific amplitude modulations. This suggests that the ITC leverages gamma oscillations not merely as a generic temporal clock for binding-by-synchrony[6], but as a multidimensional carrier wave for

population vectors—conceptually akin to AM radio communication[16]. In this framework, sensory content is structured into distinct, topologically rigid geometric paths independent of precise spike-timing relative to the oscillatory cycle."

Revised text:

"This waveform-based analysis provides mechanistic insight into how sensory information is structurally encoded within high-frequency networks. Our findings (Fig. 4) reveal that the cortex implements this code via a dynamic interaction between a condition-independent carrier oscillation and feature-specific amplitude modulations. This suggests that the ITC leverages gamma oscillations not merely as a generic temporal clock for binding-by-synchrony6, but as a multidimensional carrier wave for population vectors—conceptually akin to AM radio communication[19]. In this framework, sensory content is structured into distinct, topologically rigid geometric paths independent of precise spike-timing relative to the oscillatory cycle."

[1-7]

Figure 1b: can the authors indicate somehow which specific channels in the ECoG array are the "representative" channels shown?

Reply:

The term "representative" in the original legend was misleading. Figure 1B does not depict data from specific individual channels; rather, it displays the time-frequency representations averaged across all 128 channels in the ECoG array. We have corrected the figure legend to accurately describe this methodology and avoid further misunderstanding.

Changes to the manuscript:

We have revised the legend of Figure 1B (Page 26, Lines 941–943) to read as follows:

Original text:

"(B) Time-frequency representations of stimulus-evoked power changes. Spectrograms show the difference in 1/f-normalized power from the pre-stimulus baseline for representative ITC channels in Monkey L (left) and Monkey Q (right). …"

Revised text:

"(B) Time-frequency representations of stimulus-evoked power changes. Spectrograms show the difference in 1/f-normalized power from the pre-stimulus baseline averaged across all ECoG channels in Monkey L (left) and Monkey Q (right). …"

[1-8]

The values for the distance metrics in figure 1e&f are hard to interpret. The color scales in figure 1e and the vertical axes in figure 1f are not labeled with units. From the methods I think the units would be something like mV*Hz (measured in a 127-dimensional space), which are also hard to interpret. Some normalization would be an improvement (such as proportion of the average distance of some null distribution or similar).

Reply:

To address this concern and improve interpretability, we have normalized the distance metrics. Because the input features for our Representational Similarity Analysis (RSA) were 1/f-normalized relative power changes, the initial outputs were unitless Euclidean distances.

Following the reviewer's recommendation to implement a normalization strategy, we have scaled the Representational Dissimilarity Matrix (RDM) and linkage distances to a bounded range of [0, 1] by dividing by the maximum distance observed within each subject. This linear maximum-value normalization directly standardizes the axes for cross-subject comparison while perfectly preserving both the exact representational geometry (relative pairwise distances) and the hierarchical branching structure of the dendrograms.

Changes to the manuscript:

Figures 1E and 1F have been revised accordingly, and the corresponding figure legends have been updated to accurately state the normalization method. The corresponding figure legend have been also updated (Page 26, Lines 952–958).

Original text:

"(E) Representational dissimilarity matrices (RDMs). Matrices show the pairwise Euclidean distance between population response vectors (128 channels of gamma power) for each of the nine stimuli.

(F) Hierarchical clustering of the RDMs from (E). The dendrograms illustrate the similarity structure of the population responses to the nine stimuli."

Revised text:

"(E) Representational dissimilarity matrices (RDMs) for the nine stimulus conditions. Dissimilarity was quantified as the Euclidean distance between population response vectors (derived from the 128-channel 1/f-normalized gamma power) and subsequently max-normalized to a bounded range of [0, 1].

(F) Hierarchical clustering dendrograms illustrating the representational similarity structure of the nine stimuli. The trees were constructed by applying Ward's linkage method to the normalized distance matrices in (E)."

[1-9]

Around line 72, it's not clear at that point what "scale-invariant" means. Spatial scale? Temporal scale? Scale in some high-dimensional state space? Etc. Could be revised to be clearer.

Reply:

We completely agree with the reviewer that the term "scale-invariant" was ambiguous in this context and could cause confusion regarding physical (spatial or temporal) scales. As the reviewer correctly suspected, we intended to describe a geometric property within the high-dimensional state space—specifically, that the color-specific geometric structure of the neural trajectories (i.e., the relative topology distinguishing different colors) remains preserved even when the overall size (amplitude) of the trajectory shrinks over time.

To eliminate any ambiguity, we have revised the sentence to explicitly clarify that the invariance applies to the overall scale of the trajectory within the state space.

Changes to the manuscript:

We have revised the Introduction section (Page 2, Lines 70–72) to clarify the nature of this invariance:

Original text:

"While the absolute spatial separation of these trajectories decayed rapidly following an initial stimulus-locked transient, their scale-invariant geometric shape was conserved."

Revised text:

"While the absolute spatial separation of these trajectories decayed rapidly following an initial stimulus-locked transient, their color-specific geometric structure remained invariant to the overall scale of the trajectory within the state space."

[1-10]

Around line 74, I was a little confused about whether "shape" referred to stimulus shape or shape of a neural manifold. Could be revised to be clearer.

Reply:

We agree that using the word "shape" to refer to both the geometric properties of the neural manifold and the visual features of the stimulus could easily cause confusion. To eliminate this ambiguity, we have explicitly specified "stimulus shape" in the revised text. Please note that the surrounding text in this paragraph has also been updated to address a comment from Reviewer 2 regarding our experimental design.

Changes to the manuscript:

We have revised the Introduction section (Page 2, Lines 72–74) to clarify this term:

Original text:

"Notably, we observed a functional dissociation at this macroscopic scale: while local shape was decodable from gamma-band power, it lacked a stable oscillatory geometric structure."

Revised text:

"Furthermore, we confirmed that this geometric stability is feature-specific; while stimulus shape was decodable from gamma-band power, it lacked a stable oscillatory geometric structure "

Reviewer #2 (Remarks to the Author):

The authors record (128 channel ECoG) from the surface of the inferior temporal cortex of macaques and analyse the activity evoked by nine stimuli (3 shapes x 3 colours). Importantly, the colours were isoluminant to each other and also seemed to have been approximately isoluminant to the grey background. This is a bit unfortunate, because shapes defined by isoluminant colour contrast are not perceived and processed particularly well (e.g., Mullen & Beaudot, 2002, Vis Res; Beaudot & Mullen, 2005, Vis Res).

The results are analysed in several sophisticated and interesting ways. At the simplest level, trial-averaged gamma-power of many channels is found to distinguish stimulus colour and, to a much lesser extent, stimulus shape (Fig 1).

This finding is corroborated by a searchlight analysis -- which decodes colour/shape from 9 neighbouring channels -- and by a temporal generalisation analysis in which 128-channel decoders were trained on one 50 ms window and tested on another. The results consistently show far better representation of colour than of shape (Fig 2).

The more innovative analyses are based on demixed PCA, which divides (demixes) the high-dimensional signal (channels x time points x shapes x colours) such as to identify subspaces that are informative about 'colour', 'shape', or their 'interaction'. When applied to trial-averaged signals, this analysis confirms that more response variance distinguishes colour than shape (Fig. 3).

When applied to the time-varying signal, this analysis distinguishes between colour-dependent variance that is additive (i.e., time-independent) and that is multiplicative (i.e., colour- and time-dependent). The key finding is that multiplicative modulation explained far more variance (50%) than additive modulation (<1%). In the space of demixed PCs, particular stimulus colours elicited particular trajectories of activity (Fig. 4). The stability of these spatiotemporal trajectories was confirmed by cross-validated Procrustes similarity. In short, particular colours elicit stable and recognisable patterns of activity.

The separation (Mahalonobis distance) between trajectories increases and peaks shortly after stimulus presentation (i.e., during the first 200 ms), before returning to baseline (Fig. 5).

In contrast, stimulus shape is not encoded by stable trajectories (Fig. 6).

From my point of view, the really attractive aspects of this work are the colour-specific spatio-temporal trajectories across the inferotemporal cortex and the advanced computational methods used to extract and visualise them. Other recent work on the spatiotemporal structure of information-encoding responses in the inferior temporal cortex has not reached a comparable depth of analysis (e.g., Kakaei, Braun, 2024, Imaging Neurosci; Nigam, Schwiedrzik, 2024, Nat Comm; Nigam, Schwiedrzik, 2025, bioRxiv).

Accordingly, I would encourage the authors to focus their presentation on this aspect, rather than on the weak encoding of shape (which is owed to the stimulus set). Perhaps the authors might consider providing some more didactic visualisations of demixing PCA? I would also be fascinated to see a larger version of Fig. 4b, with trajectories depicted not as curves but as three-dimensional sausages, such as to indicate the variability from trial to trial.

This work reaches an important conclusion -- consistent spatiotemporal responses -- with highly convincing methods and deserves wide recognition. The computational methods and results could be made more accessible to general readers.

Reply:

We sincerely thank the reviewer for the highly positive evaluation of our computational approach and the extraction of color-specific spatiotemporal trajectories. To adequately address your constructive suggestions, we have broken down our responses into three specific points below.

[2-1]

Referee comment:

“Accordingly, I would encourage the authors to focus their presentation on this aspect, rather than on the weak encoding of shape (which is owed to the stimulus set).”

Reply:

As the reviewer correctly pointed out, shapes defined by isoluminant color contrast are known to be less optimally processed by the visual system (Mullen & Beaudot, 2002; Beaudot & Mullen, 2005). We fully agree that the absence of a distinct shape manifold in our dataset is primarily driven by these inherent properties of the visual stimuli, rather than an absolute inability of the ITC to encode shape dynamics.

Following your constructive recommendation, we have re-focused the manuscript. We have shifted the primary narrative away from the "functional dissociation between color and shape" and instead concentrated squarely on our core finding: the robust spatiotemporal dynamics of color representation within the gamma-band oscillatory manifold. Throughout the revised text, the shape condition is now explicitly positioned as a rigorous methodological control, demonstrating that stable geometric trajectories are not an obligatory byproduct of decodable signal power. Furthermore, in our Discussion, we have explicitly incorporated your insight regarding stimulus isoluminance as the primary explanation for the absence of a shape manifold, while repositioning our original anatomical hypothesis as a secondary, additional contributing factor.

Changes to the manuscript:

We have substantially revised the Abstract, Introduction, and Discussion to reflect this re-focusing.

Abstract (Page 1, Lines 21–24):

We have revised the text to tone down the "dissociation" and focus on the methodological contrast:

Original text:

“… Notably, although shape information was locally decodable from gamma power, it failed to form a stable geometric structure. This dissociation rules out simple signal-to-noise confounds, suggesting gamma oscillations serve as more than a generic temporal reference; they provide …”

Revised text:

"... Although stimulus shape was decodable from gamma-band power, it lacked the stable geometric structure observed for color. This contrast confirms that the oscillatory manifold is a specific representational format rather than a generic byproduct of neural activation. Consequently, gamma oscillations provide ..."

Introduction (Pages 2–3, Lines 83–91):

We have explicitly stated our focus on isoluminant color stimuli:

Original text:

"… Notably, we observed a functional dissociation at this macroscopic scale: while local shape was decodable from gamma-band power, it lacked a stable oscillatory geometric structure. This contrast indicates that the oscillatory manifold is not merely a byproduct of strong signal amplitude. …"

Revised text:

"... Furthermore, we confirmed that this geometric stability is feature-specific; while stimulus shape was decodable from gamma-band power, it lacked a stable oscillatory geometric structure. By focusing primarily on the dynamics of isoluminant color representations, we characterized the spatiotemporal evolution of this framework. …"

Discussion (Page 10, Lines 453–461):

We have integrated the reviewer's insight as the primary explanation, adding the suggested references:

Original text:

"The most parsimonious explanation for this dissociation lies in …"

Revised text:

"The most direct explanation for the absence of a distinct shape manifold in our dataset relates to the inherent properties of the visual stimuli. It is important to note that our stimulus set relied on shapes defined by isoluminant color contrast. Previous psychophysical and physiological studies have established that the visual system processes isoluminant shapes less efficiently than luminance-defined contours[38,39]. Therefore, the lack of topological stability for shape (Fig. 6) should be interpreted primarily as a consequence of these specific stimulus conditions, effectively serving as an internal methodological control.

Beyond these stimulus constraints, the most parsimonious anatomical explanation for this dissociation lies in ..." (The subsequent paragraph discusses the anatomical factor).

38. Mullen, K. T. & Beaudot, W. H. A. Comparison of color and luminance vision on a global shape discrimination task. Vis. Res. 42, 565–575 (2002).

39. Beaudot, W. H. A. & Mullen, K. T. Orientation selectivity in luminance and color vision assessed using 2-d band-pass filtered spatial noise. Vis. Res. 45, 687–696 (2005).

[2-2]

Referee comment:

“Perhaps the authors might consider providing some more didactic visualisations of demixing PCA?”

Reply:

Following the reviewer’s suggestion to include didactic visualisations of the demixing PCA framework, we have completely upgraded our initial conceptual schematic in the Main Text.

Specifically, we have replaced our original schematic with a newly designed, comprehensive methodological pipeline (new Figure 4A). This didactic visualization provides a step-by-step illustration of how the dPCA algorithm operates directly on continuous gamma-band oscillatory waveforms. It conceptually outlines the transition from multi-channel gamma waveforms (left) to the mathematical isolation of color-specific amplitude modulations from the shared temporal carrier (middle), ultimately resulting in the clear geometric segregation of 3D spatiotemporal trajectories (right). We believe this integration elevates the clarity and accessibility of our computational approach.

Changes to the manuscript:

We have completely replaced the original conceptual schematic with the new didactic pipeline schematic (Figure 4A) and updated the legend (Page 31, Lines 1148–1153).

Original text:

“(A) Conceptual framework for the geometric quantification of amplitude-modulated (AM) waveforms. Because oscillatory waveforms fluctuate around zero, stimulus information cannot be encoded via static DC offsets. Instead, it is conveyed through dynamic amplitude modulation, which dPCA isolates as the 'Interaction' component, tracing condition-specific trajectories in the state space.”

Revised text:

“(A) Methodological pipeline for the extraction of the oscillatory manifold. (Left) Gamma-band (35–80 Hz) oscillatory waveforms represented as overlapping trajectories within the multi-channel voltage space. (Middle) The dPCA algorithm decomposes these signals into condition-independent temporal dynamics (Time Mode) and color-specific modulations (Interaction Mode). (Right) The resulting condition-specific trajectories (Red, Green, Blue) projected onto the top three Interaction dPCs.”

The Results section has been also updated accordingly (Page 5, Lines 231–236).

Original text:

“Because oscillatory waveforms physically fluctuate around zero, stimulus information cannot be represented as static offsets, but must be dynamically encoded through amplitude modulations (AM) of the underlying oscillatory carrier (Fig. 4A).”

Revised text:

“Because oscillatory waveforms physically fluctuate around zero, stimulus information cannot be represented as static offsets, but must be dynamically encoded through amplitude modulations (AM) of the underlying oscillatory carrier. To explicitly isolate this condition-specific amplitude scaling from the shared high-frequency carrier, we established a methodological pipeline applying dPCA directly to the continuous gamma-band signals (Fig. 4A).”

[2-3]

Referee comments:

“I would also be fascinated to see a larger version of Fig. 4b, with trajectories depicted not as curves but as three-dimensional sausages, such as to indicate the variability from trial to trial.”

Reply:

Regarding the suggestion to visualize single-trial variability as "three-dimensional sausages" in the state space, we respectfully note a practical limitation inherent to high-dimensional, single-trial ECoG data.

Directly plotting single-trial dispersion bounds (sausages) for multiple conditions simultaneously within the 3D state space introduces severe visual occlusion due to background physiological noise. This occlusion obscures the intrinsic geometric topology and the relative spatial configuration of the condition-specific trajectories, which are the primary focus of the manifold visualization.

Instead of relying on qualitative visual bounds, our established analytical framework already mathematically integrates this trial-to-trial variability directly into our core quantitative metrics:

1. Mahalanobis Distance (Fig. 5): As detailed in the Results section, this metric explicitly scales the Euclidean separation between trajectories by the exact single-trial covariance matrix. It provides a rigorous, dynamic quantification of trajectory separability strictly relative to the single-trial variability you highlighted.

2. Cross-Validated Procrustes Analysis (Fig. 4D): This analysis confirms the structural stability of the trajectories across independent trial sets, demonstrating that the topological shape is conserved despite inter-trial noise.

Because this rigorous mathematical quantification of single-trial variance is already a central component of our pipeline (e.g., Fig. 5), we have opted to retain our current clear visualizations (solid curves) while relying on these precise statistical metrics to address trial-to-trial variability without the risk of visual occlusion.

Reviewer #3 (Remarks to the Author):

1. Brief summary of the manuscript.

This is a primate experiment carried out in higher-order inferior temporal visual areas where the authors apply a geometric data analysis to determine whether features of an oscillatory code provide a scaffold for visual information processing. The authors used amplitude modulations or raw voltage as oscillatory features, and they decoded shape and color information during a passive visual task. The authors find a positive answer during the stimulus-induced periods, where gamma amplitude modulations are robustly measurable for color processing but not for shape.

2. Overall impression of the work.

I find this paper compelling, timely, and highly relevant. The authors extend the manifold approach to oscillations, which is far less common relative to spiking data. In an effort toward transparency, the authors show the results of each monkey separately throughout the paper, which I applaud. The entire data analysis is very rigorous, combining basic signal processing with advanced geometric spatio-temporal analysis.

3. Specific comments, with recommendations for addressing each comment.

[3-1]

- Classic and recent literature regarding low dimensionality and neuronal oscillations.

Regarding the oscillatory manifold, previous attempts by other authors should be acknowledged. Here I list a few such examples within the recent literature, such as Churchland et al. (2012), Gallego-Carracedo et al. (2022), Moghaddam et al. (2025), and Polina et al. (2026). The authors should link their results with foundational work in the field of neuronal oscillations, such as the Wilson-Cowan (WC) 1972 seminal paper. The WC model links high-dimensional populations of excitatory and inhibitory neurons that can be represented in a 2-dimensional phase space. Although the authors use amplitude modulations to avoid the negative part, I think this seminal paper should be discussed because it indeed undertook such an endeavor in shaping the field.

Reply:

We highly appreciate the reviewer's insightful suggestion. We fully agree that contextualizing our findings within the broader, historical continuum of neural population dynamics significantly elevates the theoretical foundation of our manuscript.

The concept of the oscillation manifold is indeed theoretically rooted in the seminal Wilson-Cowan model, which established that macroscopic excitatory-inhibitory interactions can be formulated as dynamic trajectories within a phase space. Furthermore, we agree it is essential to explicitly position our high-gamma findings alongside traditional state-space frameworks derived from spiking activity and their recent applications to macroscopic field potentials.

As the reviewer points out, our reliance on amplitude modulations serves a specific geometric purpose. By extracting amplitude modulations (the high-gamma envelope), our approach bridges these domains; it maps the underlying phase-space population dynamics into a stable geometric format while circumventing the negative values inherent to raw voltage fluctuations. We have

expanded the Discussion section to explicitly integrate this theoretical lineage, demonstrating how this geometric formatting enables the dual nature of the manifold.

Changes to the manuscript:

We have integrated these theoretical connections into the Discussion section (Pages 8–9, Lines 380–397). The added text reads as follows:

Added text:

"The concept of the oscillatory manifold roots our findings within the broader theoretical context of neural population dynamics. Foundational work, such as the Wilson-Cowan model[20], established that macroscopic excitatory and inhibitory interactions can be formulated as dynamic trajectories within a phase space. Recent frameworks have extended this state-space perspective from spiking activity[21] to macroscopic field potentials[22–24]. By utilizing amplitude modulations rather than raw voltage, our approach bridges these domains. It maps the underlying oscillatory population dynamics into a stable geometric format, circumventing the negative values inherent to raw voltage fluctuations. This specific representational format reveals the dual nature of the manifold: it allows the network to maintain a scale-invariant topological shape for stable sensory coding, even as the absolute energy-dependent spatial separation fluctuates over time. …"

20. Wilson, H. R. & Cowan, J. D. Excitatory and inhibitory interactions in localized populations of model neurons. Biophys. J. 12, 1–24 (1972).

21. Churchland, M. M. et al. Neural population dynamics during reaching. Nature 487, 51–56 (2012).

22. Gallego-Carracedo, C., Perich, M. G., Chowdhury, R. H., Miller, L. E. & Gallego, J. Á. Local field potentials reflect cortical population dynamics in a region-specific and frequency-dependent manner. eLife 11, e73155 (2022).

23. Moghaddam, A. S., Mooziri, M., Raoufy, M. R. & Mirshekar, M. A. Olfactory bulb-medial prefrontal cortex circuit slow oscillations encode working memory representations. bioRxiv 2025.10.30.685257 (2025) doi:10.1101/2025.10.30.685257.

24. Pollina, L. et al. Decoupling simultaneous motor imagination and execution via orthogonal ECoG neural representations. Nat. Commun. 17, 4662 (2026).

[3-2]

- Role of dPCA and phantom oscillations.

There is recent work showing that oscillations can emerge after PCA decompositions, due to the orthogonality constraints implicit in dimensionality reduction and spectral analysis (sines, cosines, harmonics; see Shinn, 2023). Indeed, amplitude modulations can show peaks in the power spectrum (Nowak et al., 1992; Penttonen et al., 2003), and such amplitude modulations are, many times, coupled to the phase of slower oscillations (Tort et al., 2010; Canolty et al., 2016). It has been shown that jPCA (Churchland et al., 2012) can indeed confound traveling waves with low-dimensional rotational dynamics (Kuzmina et al., 2024). Can the authors determine whether dPCA suffers from similar limitations given the constraints described in Shinn (2023)? Note that gamma amplitude modulations could be periodic or not, and a power spectrum density of the gamma amplitude

modulations would be very informative. Both positive and negative findings would be valuable for the community, given ongoing debates about whether neuronal oscillations are epiphenomenal (van Bree et al., 2025).

Reply:

To determine whether the amplitude modulations (AM) derived from the dPCA of continuous oscillatory waveforms represent physiological dynamics or algorithmically imposed artifacts (phantom oscillations) resulting from orthogonality constraints, we performed a Power Spectral Density (PSD) analysis on the AM envelopes, as suggested.

We explicitly targeted the dynamic trajectories defining our oscillatory manifold (derived from the waveform-level dPCA presented in Fig. 4, to be clearly distinguished from the macroscopic power-level dPCA in Fig. 3). We extracted the instantaneous amplitude envelopes of the high-dimensional trajectories projected onto the dominant Interaction dPC using the Hilbert transform. After mean-centering the signals to remove DC offsets, we computed the PSD of these envelopes over the modulation frequency range (0–80 Hz) (Supplementary Fig. 6).

The PSD profiles do not exhibit sharp, narrow-band harmonic spikes (i.e., pure sines and cosines) that characterize algorithmically generated periodic interference. Instead, the AM power spectra show an aperiodic 1/f-like decay characterized by low-frequency elevations (< 20 Hz). This spectral signature reflects the transient nature of the amplitude envelope in the time domain—a stimulus-evoked burst followed by adaptation decay—rather than an artifactual periodicity imposed by dimensionality reduction constraints.

These findings rule out the mathematical caveats highlighted by Shinn (2023) and Kuzmina et al. (2024), demonstrating that the dynamic trajectories structuring the oscillatory manifold reflect genuine physiological reflections of stimulus processing. We agree that this empirical validation contributes meaningfully to ongoing debates regarding the functional reality of neuronal oscillations (van Bree et al., 2025). We have incorporated this validation into the Results section of the revised manuscript and added the requested PSD analysis as Supplementary Figure 6.

Changes to the manuscript:

We have added a new paragraph to immediately validate the physiological origin of the waveform-based neural trajectories and address theoretical caveats regarding dimensionality reduction.

Results (Page 6, Lines 250–262; Following the description of Figure 4B / Before Procrustes analysis):

Added text:

"We next examined whether these low-dimensional state-space trajectories, derived directly from continuous oscillatory waveforms, could be driven by mathematical artifacts of the dimensionality reduction pipeline. Recent theoretical work cautions that the orthogonality constraints implicit in algorithms like PCA or dPCA can project band-pass filtered noise into artifactual, purely sinusoidal "phantom oscillations"[14,15]. To rule out this possibility, we isolated the instantaneous amplitude modulation (AM) envelopes from the dominant Interaction trajectory (dPC1) using the Hilbert transform and evaluated their power spectral density (PSD) (Supplementary Fig. 6). If these dynamic

trajectories (Fig. 4) were algorithmically imposed phantom oscillations, their amplitude envelopes would manifest as periodic interference beats, resulting in sharp, narrow-band harmonic spikes in the modulation spectrum. Instead, our empirical data yielded spectra devoid of such harmonic artifacts, exhibiting an aperiodic 1/f-like decay with low-frequency elevations (< 20 Hz). This spectral signature establishes that the structuring of our oscillatory manifold reflects genuine, stimulus-evoked transient physiological dynamics rather than artificial algorithmic periodicity[16]."

14. Shinn, M. Phantom oscillations in principal component analysis. Proc. Natl. Acad. Sci. 120, e2311420120 (2023).

15. Kuzmina, E., Kriukov, D. & Lebedev, M. Neuronal travelling waves explain rotational dynamics in experimental datasets and modelling. Sci. Rep. 14, 3566 (2024).

16. van Bree, S., Levenstein, D., Krause, M. R., Voytek, B. & Gao, R. Processes and measurements: a framework for understanding neural oscillations in field potentials. Trends Cogn. Sci. 29, 448–466 (2025).

Supplementary Information (Supplementary Figure 6 Legend):

We have incorporated Supplementary Figure 6 and its corresponding legend into the Supplementary Information.

Added text:

"Supplementary Figure 6. Power spectral density of amplitude modulations.

(A and B) Amplitude modulation dynamics and corresponding spectral profiles for Monkey L (A) and Monkey Q (B). (Left panels) Instantaneous amplitude envelopes of the dominant Interaction dPC (dPC1) for the three color conditions (Red, Green, Blue) during the stimulus presentation window (0–500 ms). Envelopes were computed via the Hilbert transform of the projected oscillatory waveforms. (Right panels) Power spectral density (PSD) of the corresponding mean-centered amplitude envelopes, evaluated across the modulation frequency range (0–80 Hz). The vertical dashed line at 10 Hz is provided for visual reference of the low-frequency range."

[3-3]

- Low vs. high dimensionality reduction.

Figure 4B indicates that 13 or 20 dPCs are necessary to account for 85% of variance, but the dPCA analysis finds mainly 2 (Figure 3C). How many principal components are needed to find a low-dimensional manifold? I am not aware of an absolute threshold, and the only paper that discusses a similar issue is Humphries (2021). I encourage the authors to discuss this and to situate their work within the weak vs. strong principles of dimensionality reduction (Humphries, 2021), unless the authors find a better solution.

Reply:

The apparent discrepancy in the number of dPCs required between Figure 3C (mainly 2 components) and Figure 4B (13–20 components) stems from two methodological differences: the signal representations used and the parameters for variance partitioning.

1. Signal Representation: The two analyses operate on different representations of the ECoG signal. In Figure 3C, dPCA is applied to the gamma power envelope, extracted via the Hilbert transform. This representation reflects macroscopic amplitude dynamics, which are inherently lower-dimensional. In Figure 4B, dPCA is applied to the continuous 35–80 Hz oscillatory voltage waveforms. Capturing fast-fluctuating phase trajectories across 128 channels requires a larger set of orthogonal basis vectors to reconstruct the high-dimensional phase covariance.

2. Variance Partitioning: The analyses partitioned the signal variance differently. In Figure 4B, the marginalization included a condition-independent ‘Time’ component; thus, the 85% variance threshold incorporates the large, condition-independent visual evoked response. In Figure 3C, the dPCA explicitly targeted task-modulated components ('Color', 'Shape', and 'Interaction'). Within this condition-dependent subspace, the geometry is captured by fewer components

Changes to the manuscript:

We have added a new paragraph to the Discussion (Page 9, Lines 397–407) to situate our findings within the framework of Humphries (2021):

Added text:

“… Furthermore, analyzing this geometry across different mathematical representations addresses a critical theoretical question regarding dimensionality. The apparent dimensionality of a neural manifold is bounded by the signal representation and the variance partitioned[25]. Estimating the dimensionality of continuous oscillatory waveforms inherently requires a larger basis set to capture fast-fluctuating phase covariance compared to the lower-dimensional dynamics of macroscopic power envelopes. Despite this difference in mathematical basis, the topological segregation of color information remained conserved across both analyses. This consistency aligns with the “strong principle” of neural dimension reduction[25], where the low-dimensional manifold is not merely a convenient mathematical tool for data visualization, but rather reflects the actual latent variables utilized by neural circuits to operate and compute.”

25. Humphries, M. D. Strong and weak principles of neural dimension reduction. arXiv 5, (2020).

Furthermore, we have revised the legend for Figure 3C (Page 29, Lines 1127–1132) to accurately state the variance metrics plotted, removing ambiguity:

Original text:

“(C) Quantification of task-relevant variance by dPCA for the two monkeys. Bar plots show the percentage of variance accounted for (VAF) by the top 8 demixed principal components (dPCs). Each bar is colored according to its algorithm-assigned identity (Color-related: teal; Shape-related: gold; Interaction: red). Pie charts show the relative percentage of the total task-modulated variance

(i.e., excluding the large condition-independent component) attributable to all Color, Shape, or Interaction components combined.”

Revised text:

"(C) Quantification of task-relevant variance by dPCA for the two monkeys. Bar plots show the percentage of variance accounted for (VAF) by the top 8 demixed principal components (dPCs). Each bar is colored according to its marginalized identity (Color-related: teal; Shape-related: yellow; Interaction: red). Pie charts illustrate the relative percentage of the total task-modulated variance (i.e., isolating the condition-dependent signal by excluding the large condition-independent component) attributable to all Color, Shape, or Interaction components combined."

Response to the Reviewers' Comments

Reviewer #1 (Remarks to the Author):

My concerns have been addressed. Minor improvement to the revision: the font size for the value labels overlaid on the 3x3 similarity matrices in the figures could be made larger for readability

Reply:

We thank the reviewer for the positive evaluation and the constructive suggestion. In accordance with this recommendation, we have modified all relevant figures containing the 3x3 similarity matrices (including Figure 4D, Figure 5B, Figure 6B,C, Supplementary Figure 8) by increasing the font size of the overlaid numerical value labels to ensure optimal readability.

Changes to the manuscript:

The revised high-resolution panels with enlarged value labels have been incorporated into the final figures. No changes were required in the text or figure legends, as this modification is strictly graphical.

Reviewer #2 (Remarks to the Author):

The authors have adequately addressed all of my comments. They have added an explanatory figure panel (Fig. 4A) to illustrate their demixing PCA framework. The discussion now emphasizes oscillatory encoding of color information, rather than the weak encoding of shape information. They relate their findings to neural population dynamics and discuss the spatial resolution of their data. I do not see a need for further revisions.

Reply:

We sincerely appreciate the reviewer's time, clear guidance, and support throughout the evaluation process. We are pleased that the newly added framework schematic (Figure 4A) and the revised Discussion have successfully addressed all points to the reviewer's satisfaction.

Reviewer #3 (Remarks to the Author):

I thank the authors for their thorough and detailed point-by-point replies. They addressed all of the concerns raised. Congratulations on the great work.

Reply:

We are grateful to the reviewer for the encouraging summary and the constructive feedback provided during the revision process, which substantially strengthened the manuscript.
