# Supplementary material for: Gamma oscillations provide a stable geometric scaffold for color representation in primate inferior temporal cortex
Source: Commun Biol. 2026 Jul 11;9:1455. doi: 10.1038/s42003-026-10652-8 (PMC13737192; doi:10.1038/s42003-026-10652-8)
Supplement: Supplementary file 2 [file 42003_2026_10652_MOESM2_ESM.pdf]

Supplementary Materials for

Gamma oscillations provide a stable geometric scaffold for color representation in primate inferior temporal cortex

Chengpeng Li,[1,2,3,4] Isao Hasegawa,[5] and Hisashi Tanigawa[1,2,3,4,5,*]

[1]School of Brain Science and Brain Medicine, Zhejiang University, Hangzhou, China

[2]College of Biomedical Engineering and Instrument Science, Zhejiang University, Hangzhou, China

[3]MOE Frontier Science Center for Brain Science and Brain-Machine Integration, School of Brain Science and Brain Medicine, Zhejiang University, Hangzhou, China

[4]National Key Laboratory of Brain and Computer Intelligence, Zhejiang University, Hangzhou, China

[5]Department of Physiology, Niigata University School of Medicine, Niigata, Japan

*** Correspondence:**
Hisashi Tanigawa
hisashi.q@gmail.com

The PDF file includes: Supplementary Figures 1 to 8

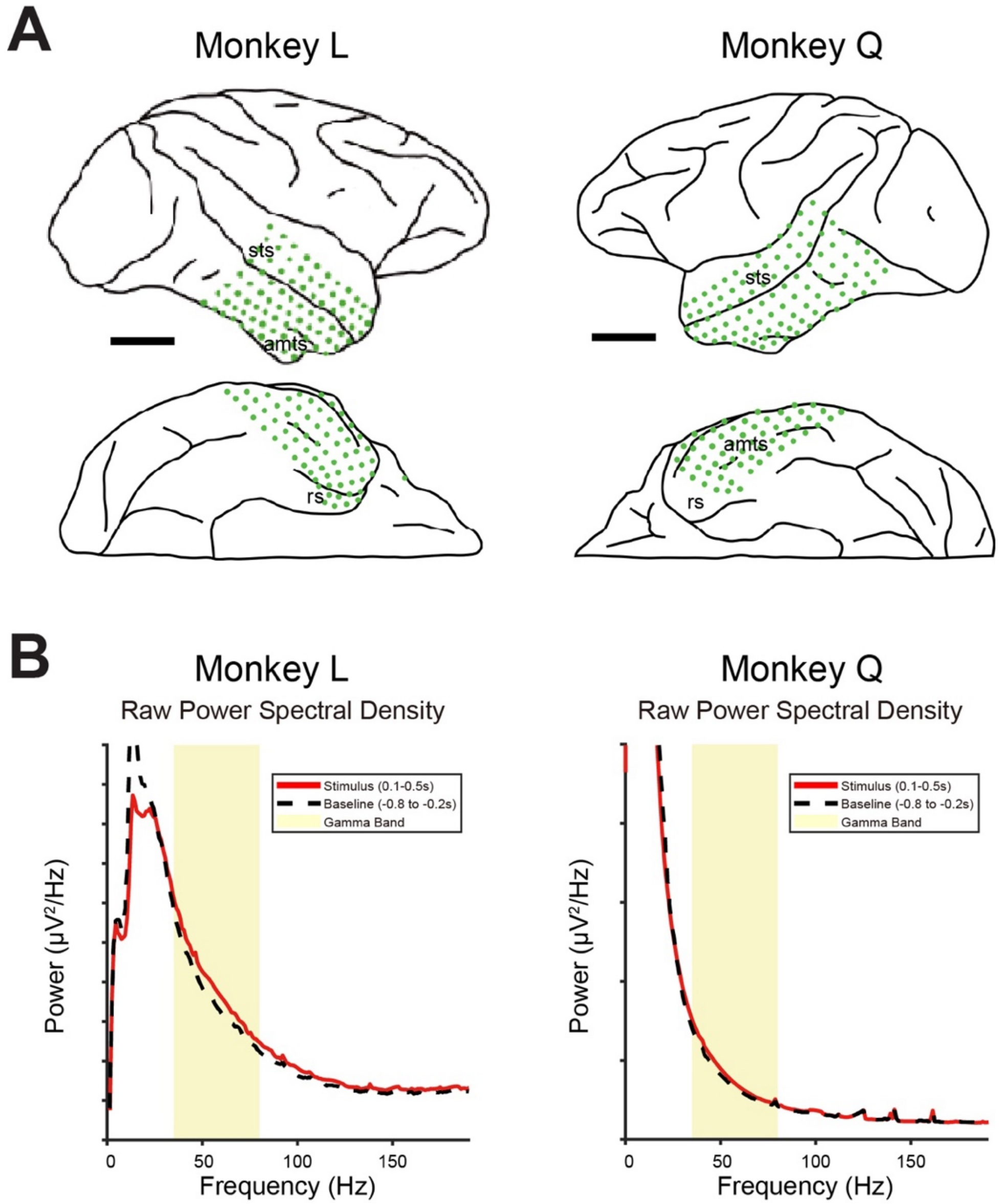

Supplementary Figure 1: Anatomical localization of ECoG arrays and raw power spectral density profiles.

(A) ECoG electrode localization. Lateral and ventral views illustrate the placement of the high-density 128-channel subdural ECoG arrays over the inferior temporal cortex (ITC) for Monkey L and Monkey Q. Green dots represent individual recording electrodes. Major sulci are labeled: STS (superior temporal sulcus), AMTS (anterior middle temporal sulcus), and RS (rhinal sulcus). Electrode positions were confirmed post-mortem. Scale bars, 1 cm.

(B) Raw Power Spectral Density (PSD) during stimulus and baseline periods. The plots show the absolute mean power spectrum during the stimulus presentation window (0.1–0.5 s, red solid line) and the pre-stimulus baseline window (–0.8 to –0.2 s, black dashed line) averaged across all ITC channels for Monkey L and Monkey Q, respectively. The yellow shaded region indicates the gamma band, where an elevation in absolute power is observed during the stimulus period relative to baseline.

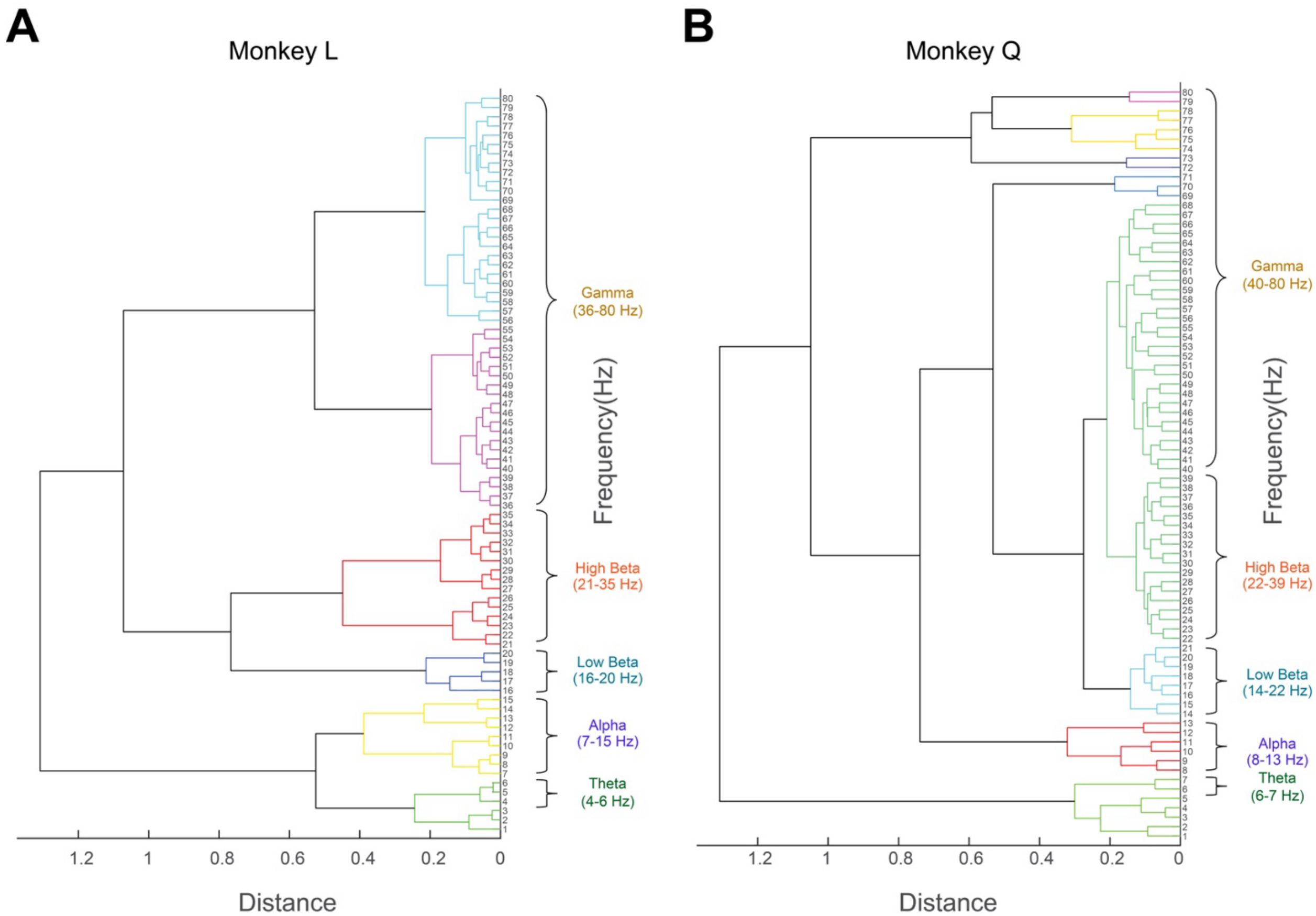

Supplementary Figure 2: Identification of functional frequency bands via hierarchical clustering.

(A and B) Dendrograms resulting from hierarchical clustering of representational similarity across frequencies (1–80 Hz) for Monkey L **(A)** and Monkey Q **(B)**. The y axis represents dissimilarity (linkage distance) between frequency power profiles. Colored brackets and text adjacent to the dendrogram indicate the specific frequency boundaries and ranges defined for each animal. Band labels (e.g., Theta, Gamma) correspond to conventional frequency ranges overlapping with the identified clusters.

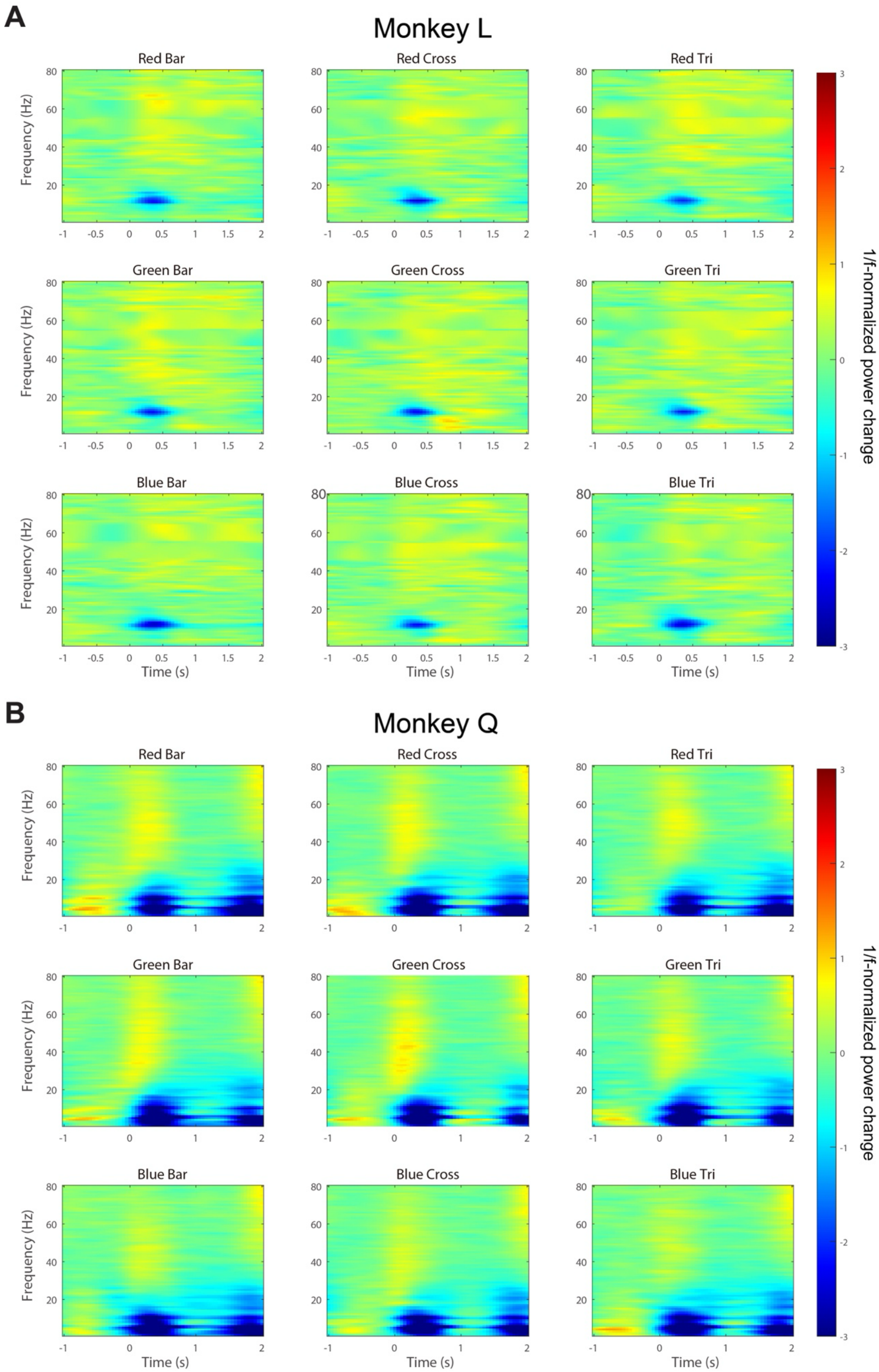
A
Monkey L
Red Bar
Red Cross
Red Tri
Green Bar
Green Cross
Green Tri
Blue Bar
Blue Cross
Blue Tri
Frequency (Hz)
Time (s)
1/f-normalized power change
B
Monkey Q
Red Bar
Red Cross
Red Tri
Green Bar
Green Cross
Green Tri
Blue Bar
Blue Cross
Blue Tri
Frequency (Hz)
Time (s)
1/f-normalized power change

Supplementary Figure 3: Identification of Narrow-band Gamma Oscillations in ITC.

(A and B) Time-frequency representations (TFRs) for nine unique stimulus exemplars in Monkey L (A) and Monkey Q (B). Each panel displays the difference in 1/f-normalized power from the pre-stimulus baseline averaged across the population (128 channels) for a specific color-shape combination (e.g., Red Bar, Green Cross). A sustained power elevation in the gamma range (~40–80 Hz) is consistently observed following stimulus onset (t = 0 s) across all conditions.

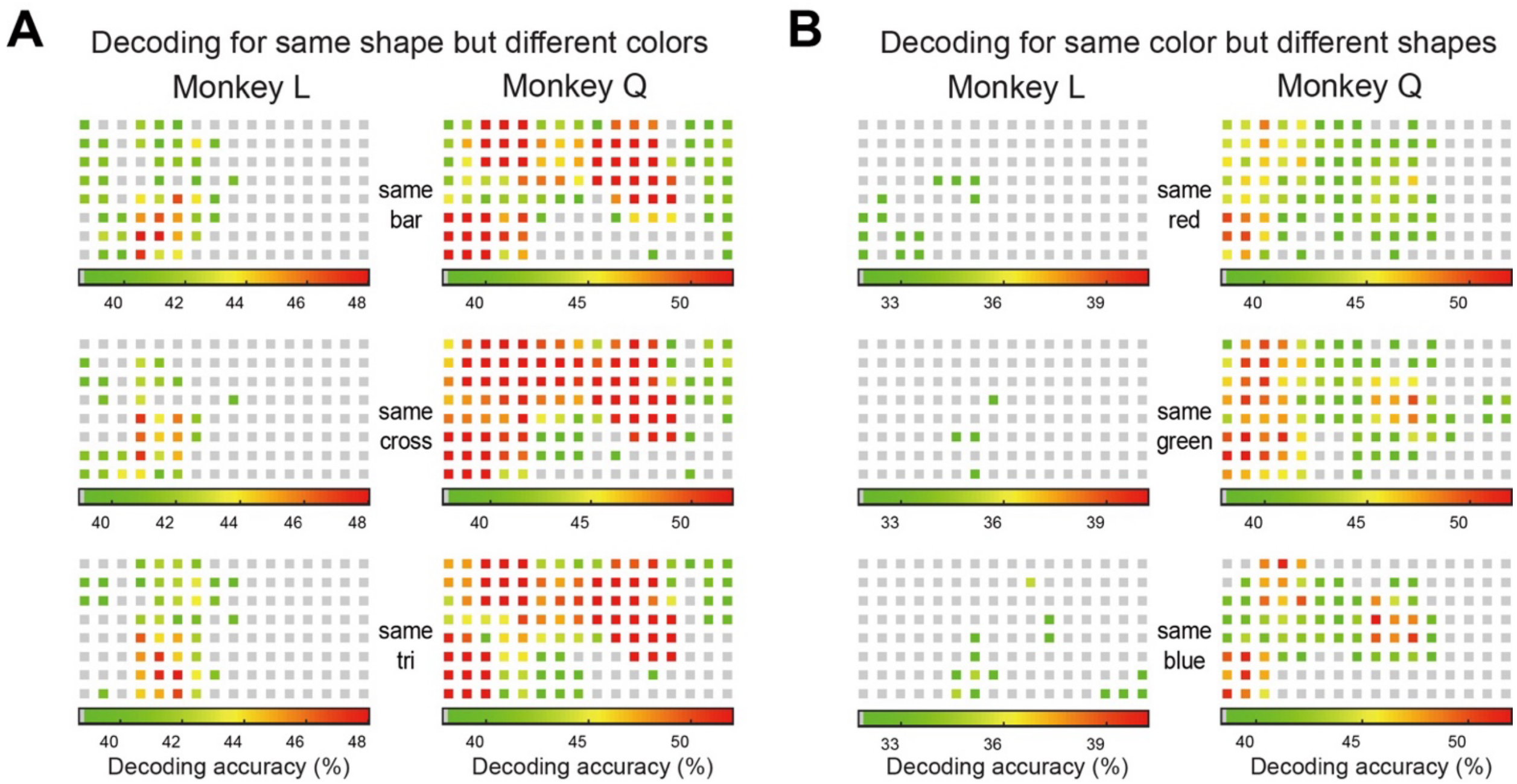

Supplementary Figure 4: Control analyses for feature decoding using stratified sampling.

(A) Spatial maps of color decoding accuracy derived from gamma-band power (0–500 ms) using a stratified searchlight analysis. Color identity was decoded exclusively using trials where the stimulus shape was identical. Same conventions as Fig. 2A.

(B) Spatial maps of shape decoding accuracy derived using trials where the stimulus color was identical. Shape identity was decoded. Same conventions as Fig. 2C.

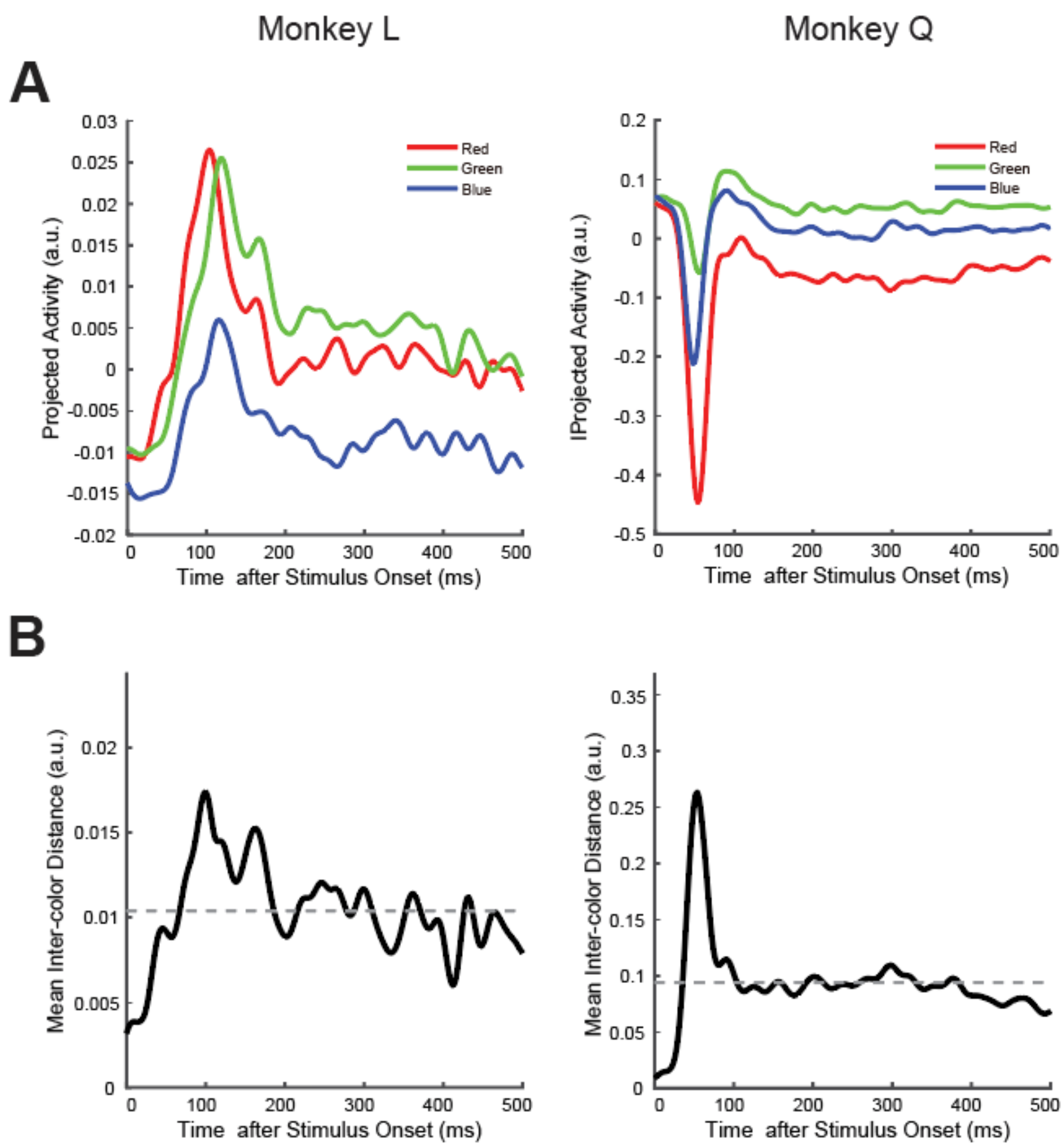

Supplementary Figure 5: Temporal dynamics of the Interaction subspace in the power envelope.

(A) Temporal evolution of Interaction dPC 1 ('Color × Time' component) for each color condition (averaged across shapes).

(B) Mean inter-color Euclidean distance computed within the dominant Color subspace over time. The gray dashed line indicates the time-averaged mean inter-color distance across the entire stimulus presentation window (0–500 ms).

Columns represent subjects (Monkey L and Monkey Q).

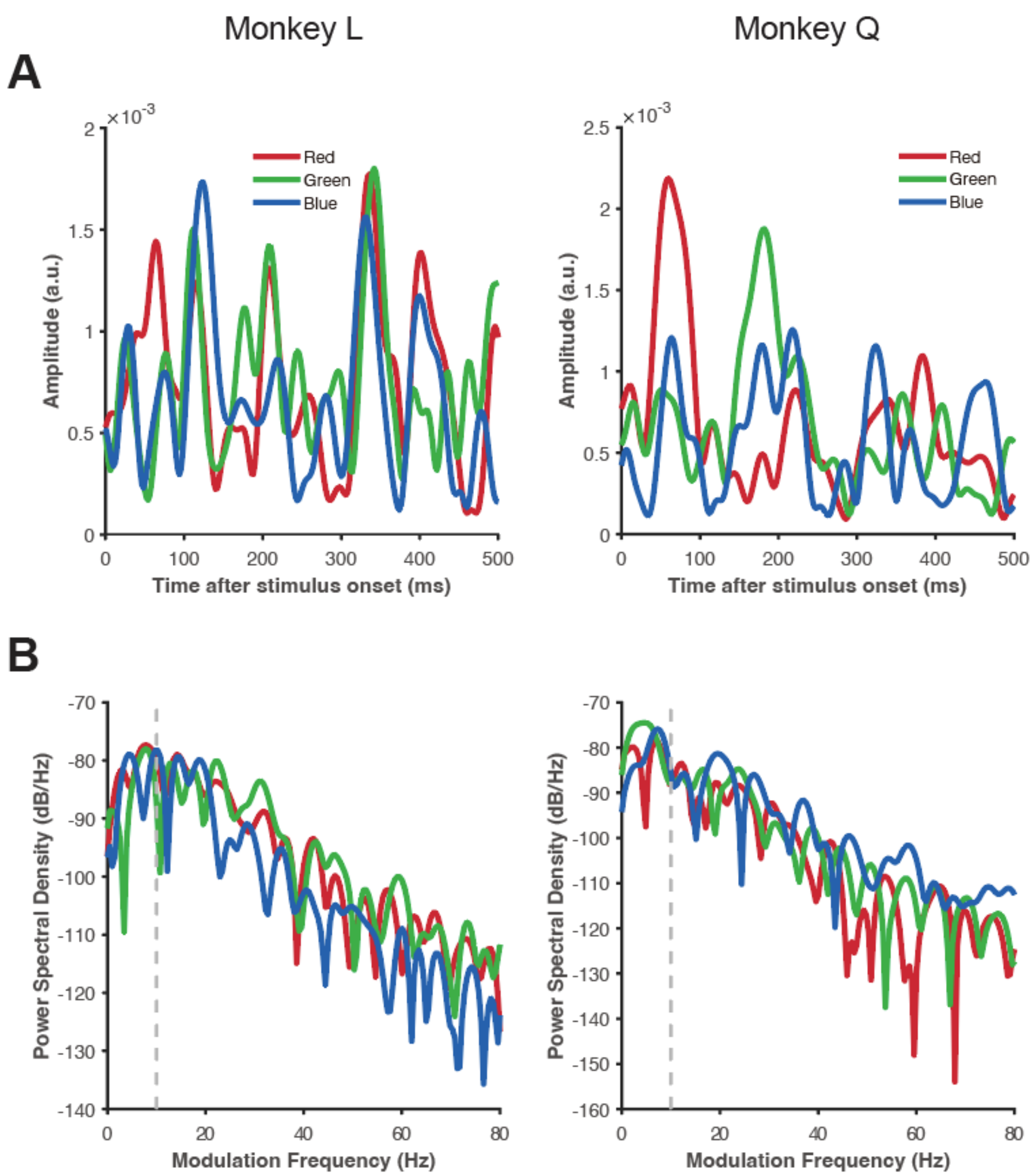

Supplementary Figure 6: Power spectral density of amplitude modulations.

(A) Instantaneous amplitude envelopes of the dominant Interaction dPC (dPC1) for the three color conditions (Red, Green, Blue) during the stimulus presentation window (0–500 ms). Envelopes were computed via the Hilbert transform of the projected oscillatory waveforms.

(B) Power spectral density (PSD) of the corresponding mean-centered amplitude envelopes, evaluated across the modulation frequency range (0–80 Hz). The vertical dashed line at 10 Hz is provided for visual reference of the low-frequency range.

Columns represent subjects (Monkey L and Monkey Q).

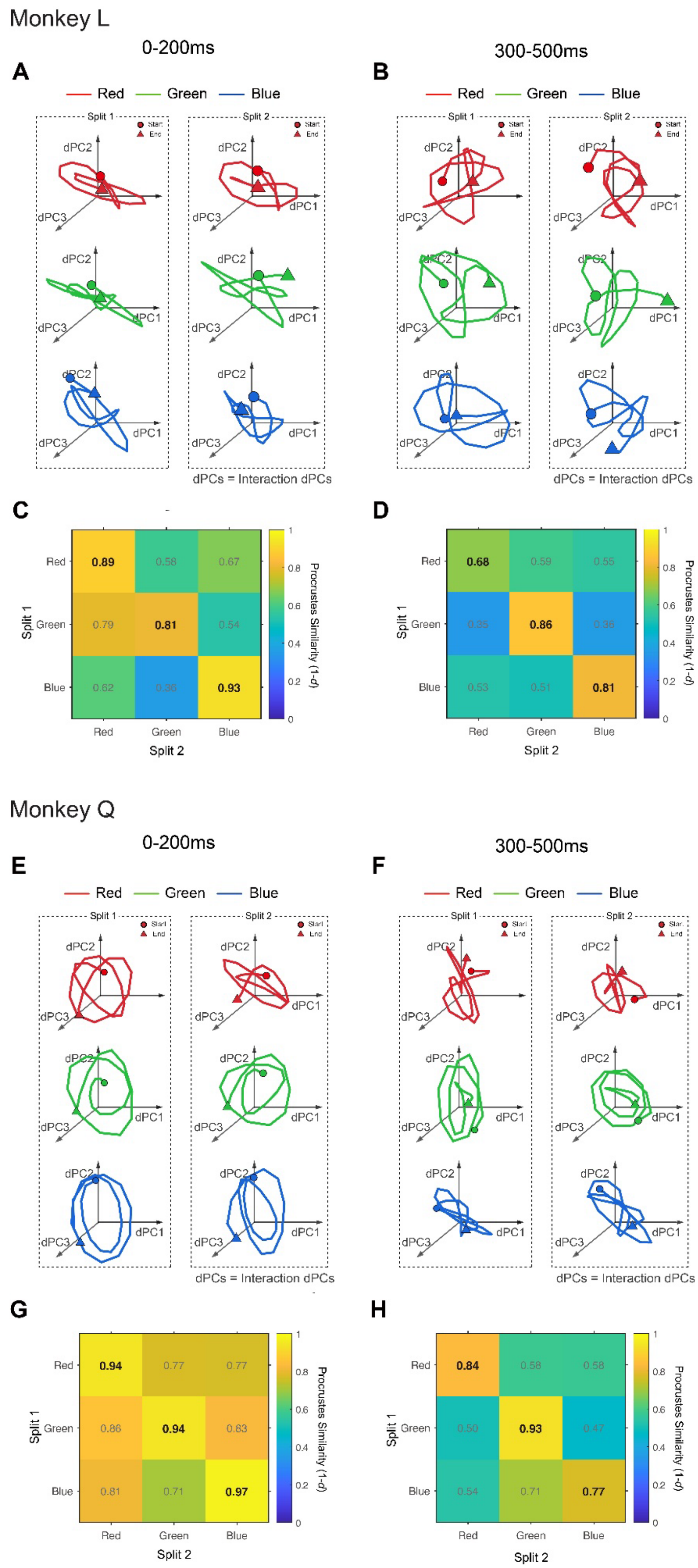
Monkey L
0-200ms
300-500ms
A
B
Red
Green
Blue
Split 1
Split 2
Start
End
dPC1
dPC2
dPC3
dPCs = Interaction dPCs
C
D
Red
Green
Blue
Split 1
Split 2
0.89
0.58
0.67
0.79
0.81
0.54
0.62
0.36
0.93
0.68
0.59
0.55
0.35
0.86
0.36
0.53
0.51
0.81
Procrustes Similarity (1-d)
0
0.2
0.4
0.6
0.8
1
Monkey Q
0-200ms
300-500ms
E
F
G
H
0.94
0.77
0.77
0.86
0.94
0.83
0.81
0.71
0.97
0.84
0.58
0.58
0.50
0.93
0.47
0.54
0.71
0.77

Supplementary Figure 7: Topological conservation of color-specific trajectories across temporal epochs and subjects.

(A and B) 3D oscillatory trajectories for Monkey L derived from a representative 100-ms window within the early transient phase (0–200 ms; A) and the sustained phase (300–500 ms; B). Same conventions as Figure 4C. For visualization, the axes of Split 2 are sign-flipped to resolve the inherent sign-ambiguity of dPCA eigenvectors.

(C and D) Cross-validated Procrustes similarity matrices for Monkey L during the early transient phase (0–200 ms; C) and the sustained phase (300–500 ms; D). Same conventions as Figure 4D.

(E and F) 3D oscillatory trajectories for Monkey Q derived from a representative 100-ms window within the early transient phase (0–200 ms; E) and the sustained phase (300–500 ms; F). Same conventions as (A and B).

(G and H) Cross-validated Procrustes similarity matrices for Monkey Q during the early transient phase (0–200 ms; G) and the sustained phase (300–500 ms; H). Same conventions as (C and D).

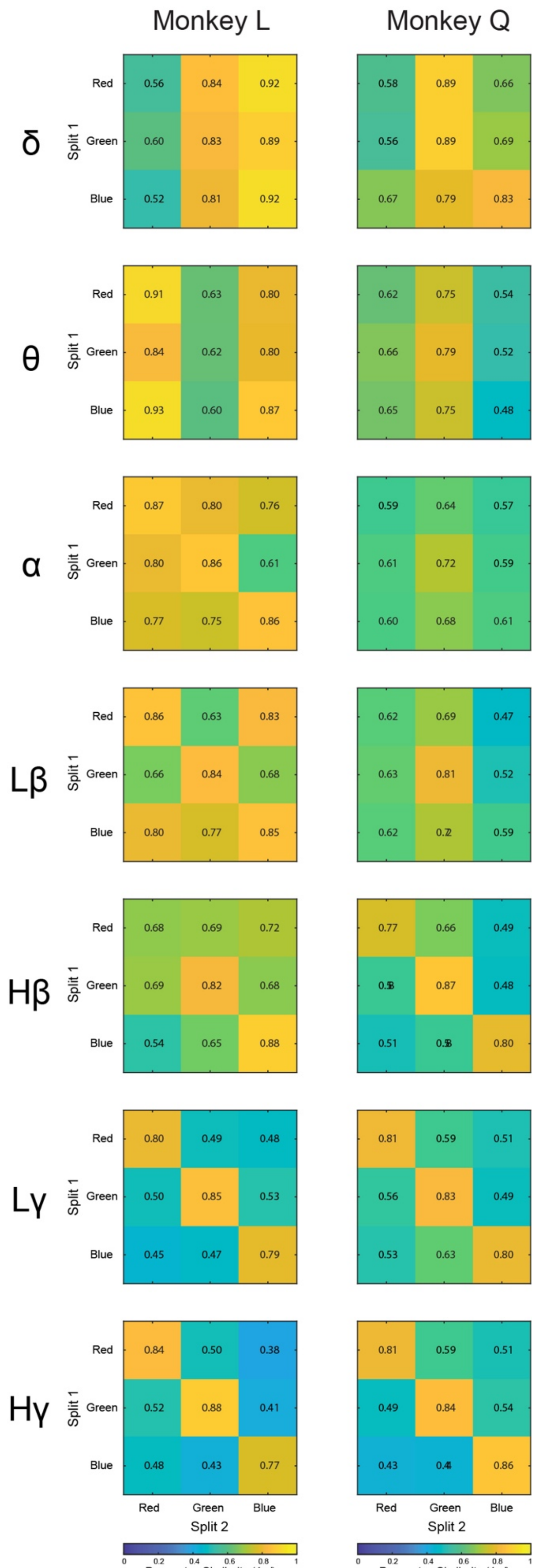

Monkey L
Monkey Q
δ
θ
α
Lβ
Hβ
Lγ
Hγ
Split 1
Red
Green
Blue
0.56 0.84 0.92
0.60 0.83 0.89
0.52 0.81 0.92
0.58 0.89 0.66
0.56 0.89 0.69
0.67 0.79 0.83
0.91 0.63 0.80
0.84 0.62 0.80
0.93 0.60 0.87
0.62 0.75 0.54
0.66 0.79 0.52
0.65 0.75 0.48
0.87 0.80 0.76
0.80 0.86 0.61
0.77 0.75 0.86
0.59 0.64 0.57
0.61 0.72 0.59
0.60 0.68 0.61
0.86 0.63 0.83
0.66 0.84 0.68
0.80 0.77 0.85
0.62 0.69 0.47
0.63 0.81 0.52
0.62 0.59
0.68 0.69 0.72
0.69 0.82 0.68
0.54 0.65 0.88
0.77 0.66 0.49
0.87 0.48
0.51 0.80
0.80 0.49 0.48
0.50 0.85 0.53
0.45 0.47 0.79
0.81 0.59 0.51
0.56 0.83 0.49
0.53 0.63 0.80
0.84 0.50 0.38
0.52 0.88 0.41
0.48 0.43 0.77
0.81 0.59 0.51
0.49 0.84 0.54
0.43 0.86
Split 2
0 0.2 0.4 0.6 0.8 1
Procrustes Similarity (1-d)

Supplementary Figure 8: Frequency specificity of geometric stability in neural trajectories.

Cross-validated Procrustes similarity matrices for neural trajectories derived from seven distinct frequency bands. Columns represent subjects (Monkey L and Monkey Q). Rows correspond to different frequency bands, ranging from lower frequencies (Delta to High Beta) to two narrower sub-bands within the Gamma range (Low-Gamma and High-Gamma). Same conventions as Figure 4D.
