# Supplementary material for: Gamma oscillations provide a stable geometric scaffold for color representation in primate inferior temporal cortex
Source: Commun Biol. 2026 Jul 11;9:1455. doi: 10.1038/s42003-026-10652-8 (PMC13737192; doi:10.1038/s42003-026-10652-8)
Supplement: Supplementary file 3 [file 42003_2026_10652_MOESM3_ESM.pdf]

Description of Additional Supplementary Files

File name: Supplementary Data 1

Description: The numerical source data underlying all graphs and charts in the main and supplementary figures are provided as Supplementary Data 1.
