# Supplementary material for: Gamma oscillations provide a stable geometric scaffold for color representation in primate inferior temporal cortex
Source: Commun Biol. 2026 Jul 11;9:1455. doi: 10.1038/s42003-026-10652-8 (PMC13737192; doi:10.1038/s42003-026-10652-8)
Supplement: Supplementary file 4 [file 42003_2026_10652_MOESM5_ESM.pdf]

nature portfolio

Corresponding author(s): Hisashi Tanigawa

Last updated by author(s): Jun 26, 2026

# Reporting Summary

Nature Portfolio wishes to improve the reproducibility of the work that we publish. This form provides structure for consistency and transparency in reporting. For further information on Nature Portfolio policies, see our Editorial Policies and the Editorial Policy Checklist.

## Statistics

For all statistical analyses, confirm that the following items are present in the figure legend, table legend, main text, or Methods section.

| n/a | Confirmed | |
|---|---|---|
| ☐ | ☒ | The exact sample size (*n*) for each experimental group/condition, given as a discrete number and unit of measurement |
| ☐ | ☒ | A statement on whether measurements were taken from distinct samples or whether the same sample was measured repeatedly |
| ☐ | ☒ | The statistical test(s) used AND whether they are one- or two-sided<br>*Only common tests should be described solely by name; describe more complex techniques in the Methods section.* |
| ☒ | ☐ | A description of all covariates tested |
| ☐ | ☒ | A description of any assumptions or corrections, such as tests of normality and adjustment for multiple comparisons |
| ☐ | ☒ | A full description of the statistical parameters including central tendency (e.g. means) or other basic estimates (e.g. regression coefficient) AND variation (e.g. standard deviation) or associated estimates of uncertainty (e.g. confidence intervals) |
| ☐ | ☒ | For null hypothesis testing, the test statistic (e.g. *F*, *t*, *r*) with confidence intervals, effect sizes, degrees of freedom and *P* value noted<br>*Give P values as exact values whenever suitable.* |
| ☒ | ☐ | For Bayesian analysis, information on the choice of priors and Markov chain Monte Carlo settings |
| ☒ | ☐ | For hierarchical and complex designs, identification of the appropriate level for tests and full reporting of outcomes |
| ☐ | ☒ | Estimates of effect sizes (e.g. Cohen's *d*, Pearson's *r*), indicating how they were calculated |

*Our web collection on statistics for biologists contains articles on many of the points above.*

## Software and code

Policy information about availability of computer code

| | |
|---|---|
| Data collection | Stimulus presentation, liquid reward delivery, and eye-tracking were fully automated using custom experimental control software. Electrophysiological ECoG signals were acquired, filtered, and digitized using a hardware-software integrated system from Plexon Inc. (Plexon PBX system). |
| Data analysis | All offline signal preprocessing, time-frequency decomposition, and statistical or geometric analyses were performed using MATLAB (MathWorks, MA). The specific software toolboxes and libraries utilized include:<br>FieldTrip Toolbox: Used for advanced analysis of electrophysiological data, including time-frequency wavelet transforms and statistical clustering.<br>LIBLINEAR Library: Used for training and testing the linear Support Vector Machine (SVM) classifiers in the searchlight and cross-temporal decoding analyses.<br>Custom dPCA and Procrustes Frameworks: Implemented in MATLAB based on published algorithms to construct, project, and align the multidimensional neural trajectories. Code Deposition: All custom scripts and pipelines developed for the main geometric analyses (e.g., dPCA, Procrustes trajectory alignment), multivariate decoding (MVPA), and time-frequency transformations have been made publicly available in open-access repositories via Zenodo under the following DOIs:<br>Main geometric and decoding analysis code: https://doi.org/10.5281/zenodo.20566247<br>Time-frequency transform and hierarchical clustering code: https://doi.org/10.5281/zenodo.15203774 |

For manuscripts utilizing custom algorithms or software that are central to the research but not yet described in published literature, software must be made available to editors and reviewers. We strongly encourage code deposition in a community repository (e.g. GitHub). See the Nature Portfolio guidelines for submitting code & software for further information.

## Data

Policy information about availability of data

All manuscripts must include a data availability statement. This statement should provide the following information, where applicable:

- Accession codes, unique identifiers, or web links for publicly available datasets
- A description of any restrictions on data availability
- For clinical datasets or third party data, please ensure that the statement adheres to our policy

Custom code and raw data (~16 GB) for the main geometric analyses (e.g., demixed Principal Component Analysis) and neural decoding analysis (Multivariate Pattern Analysis), which includes the raw electrophysiological data from one of the subjects, are publicly available at Zenodo (https://doi.org/10.5281/zenodo.20566247).

The code and sample datasets for the time-frequency transform process and hierarchical clustering of frequency bands are available at Zenodo (https://doi.org/10.5281/zenodo.15203774).

The numerical source data underlying all graphs and charts in the main and supplementary figures are provided with the manuscript as Supplementary Data 1.

The remaining full raw dataset (~33 GB) is available from the corresponding author upon reasonable request. No institutional or legal restrictions apply to data access. This study did not utilize clinical or third-party datasets.

## Research involving human participants, their data, or biological material

Policy information about studies with human participants or human data. See also policy information about sex, gender (identity/presentation), and sexual orientation and race, ethnicity and racism.

| | |
|---|---|
| Reporting on sex and gender | Not applicable. This study did not involve human participants, human data, or human biological material. |
| Reporting on race, ethnicity, or other socially relevant groupings | Not applicable. This study did not involve human participants or human data. |
| Population characteristics | Not applicable. This study did not involve human participants or human data. |
| Recruitment | Not applicable. This study did not involve human participants. |
| Ethics oversight | Not applicable. No human participants, data, or tissues were utilized. |

Note that full information on the approval of the study protocol must also be provided in the manuscript.

# Field-specific reporting

Please select the one below that is the best fit for your research. If you are not sure, read the appropriate sections before making your selection.

☒ Life sciences ☐ Behavioural & social sciences ☐ Ecological, evolutionary & environmental sciences

For a reference copy of the document with all sections, see nature.com/documents/nr-reporting-summary-flat.pdf

# Life sciences study design

All studies must disclose on these points even when the disclosure is negative.

| | |
|---|---|
| Sample size | Two adult Japanese monkeys (Macaca fuscata) were used in this study. The dataset for each monkey was compiled from three recording sessions, yielding a total of 1,836 successful trials for Monkey L and 2,106 successful trials for Monkey Q. This provided a minimum of 200 trials for each of the nine unique stimulus conditions. A sample size of two animals is standard for non-human primate electrophysiology studies, balancing the need to demonstrate reproducibility across subjects with ethical mandates for the reduction of animal use. |
| Data exclusions | No data were excluded from the analysis after the successful completion of the behavioral trials. The final dataset included all successful trials where the animals correctly maintained central fixation within the required window throughout the trial duration. |
| Replication | All main experimental findings, including feature selectivity maps, cross-temporal decoding profiles, and oscillatory manifold structures, were replicated across the two individual subjects (Monkey L and Monkey Q). To ensure the statistical reproducibility of the decoding accuracies and state-space trajectories, cross-validation and data-splitting strategies were implemented. Specifically, a 10-fold cross-validation procedure was used for the multivariate decoding analyses , and independent, non-overlapping data splits (Split 1 and Split 2) were utilized for the Procrustes trajectory alignment and time-resolved Mahalanobis distance analyses. |
| Randomization | The visual stimuli, consisting of a 3 x 3 factorial combination of color and shape, were presented in a pseudorandom order across trials during the passive viewing task. For the multivariate pattern analysis (MVPA) and demixed Principal Component Analysis (dPCA), the number of trials for each condition was balanced via random subsampling to eliminate classification and projection biases. |

| | |
|---|---|
| Blinding | Due to the within-subject experimental design, the experimenters were not blinded to the stimulus conditions during data collection. However, potential experimenter bias was minimized because stimulus presentation, reward delivery, and eye-tracking monitoring were fully automated. Furthermore, all subsequent signal preprocessing, artifact removal, and statistical analyses were executed using automated scripts to eliminate subjective bias. |

# Reporting for specific materials, systems and methods

We require information from authors about some types of materials, experimental systems and methods used in many studies. Here, indicate whether each material, system or method listed is relevant to your study. If you are not sure if a list item applies to your research, read the appropriate section before selecting a response.

## Materials & experimental systems

| n/a | Involved in the study | |
|---|---|---|
| ☒ | ☐ | Antibodies |
| ☒ | ☐ | Eukaryotic cell lines |
| ☒ | ☐ | Palaeontology and archaeology |
| ☐ | ☒ | Animals and other organisms |
| ☒ | ☐ | Clinical data |
| ☒ | ☐ | Dual use research of concern |
| ☒ | ☐ | Plants |

## Methods

| n/a | Involved in the study | |
|---|---|---|
| ☒ | ☐ | ChIP-seq |
| ☒ | ☐ | Flow cytometry |
| ☒ | ☐ | MRI-based neuroimaging |

## Animals and other research organisms

Policy information about studies involving animals; ARRIVE guidelines recommended for reporting animal research, and Sex and Gender in Research

| | |
|---|---|
| Laboratory animals | Two adult Japanese macaques (Macaca fuscata) were used in this study: one female (Monkey L, body weight 5.6 kg) and one male (Monkey Q, body weight 7.1 kg). At the time of the experiments, the animals were aged between 4 and 7 years. Both monkeys were purpose-bred for research and sourced from the National BioResource Project (NBRP) "Japanese Monkeys" of the Ministry of Education, Culture, Sports, Science and Technology (MEXT), Japan.<br><br>The animals were housed individually in standard primate cages within an air-conditioned room under a regulated 14:10-hour light-dark cycle. Environmental enrichment (e.g., toys and foraging devices) was continuously provided. The animals maintained a standard primate diet supplemented daily with fresh fruits and vegetables, with water available ad libitum. |
| Wild animals | The study did not involve wild animals. |
| Reporting on sex | Both a female (Monkey L) and a male (Monkey Q) macaque were included in this study to ensure that the neurophysiological findings were not restricted to a single sex. Because electrophysiological recordings from non-human primates require intensive, long-term intra-subject data collection across thousands of trials, data from each animal were analyzed and reported separately.<br><br>All core findings—including the low-dimensional structure of the oscillatory manifold, the functional dissociation between color and shape geometries, and the topological stability of representational trajectories—were successfully and independently replicated in both the female and male subjects, demonstrating that the observed neural mechanisms are consistent across sexes.Sex-based stratification of the pooled data was not performed due to the standard within-subject design optimized for individual-level cognitive electrophysiology. |
| Field-collected samples | The study did not involve field-collected samples. |
| Ethics oversight | All experimental and surgical procedures were reviewed and explicitly approved by the Institutional Animal Care and Use Committee of Niigata University (Permission number: 27-184-1). All animal care, housing, and experimental protocols strictly adhered to the Japanese Act on Welfare and Management of Animals, the Fundamental Guidelines for Proper Conduct of Animal Experiment and Related Activities in Academic Research Institutions under MEXT, Japan, and the National Institutes of Health (NIH) Guide for the Care and Use of Laboratory Animals. |

Note that full information on the approval of the study protocol must also be provided in the manuscript.

## Plants

| | |
|---|---|
| Seed stocks | Not applicable. This study did not involve or generate any novel plant genotypes. |
| Novel plant genotypes | Not applicable. No plant materials or seed stocks were utilized in this study. |
| Authentication | Not applicable. This study did not utilize any plant samples requiring taxonomic or genetic authentication. |
